# Protective intraoperative ventilation with higher versus lower levels of positive end-expiratory pressure in obese patients (PROBESE): study protocol for a randomized controlled trial

**DOI:** 10.1186/s13063-017-1929-0

**Published:** 2017-04-28

**Authors:** T. Bluth, R. Teichmann, T. Kiss, I. Bobek, J. Canet, G. Cinnella, L. De Baerdemaeker, C. Gregoretti, G. Hedenstierna, S. N. Hemmes, M. Hiesmayr, M. W. Hollmann, S. Jaber, J. G. Laffey, M. J. Licker, K. Markstaller, I. Matot, G. Müller, G. H. Mills, J. P. Mulier, C. Putensen, R. Rossaint, J. Schmitt, M. Senturk, A. Serpa Neto, P. Severgnini, J. Sprung, M. F. Vidal Melo, H. Wrigge, M. J. Schultz, P. Pelosi, M. Gama de Abreu, for the PROBESE investigators, Andreas Güldner, Robert Huhle, Christopher Uhlig, Luigi Vivona, Alice Bergamaschi, Rolf Rossaint, Ana Stevanovic, Tanja Treschan, Maximilian Schaefer, Peter Kienbaum, Rita Laufenberg-Feldmann, Lars Bergmann, Felix Ebner, Luisa Robitzky, Patrick Mölders, Matthias Unterberg, Cornelius Busch, Marc Achilles, Angelika Menzen, Harbert Freesemann, Christian Putensen, Humberto Machado, Carla Cavaleiro, Cristina Ferreira, Daniela Pinho, Marta Carvalho, Sílvia Pinho, Maria Soares, Diogo Sousa Castro, Fernando Abelha, Rui Rabico, Ellise Delphin, Juraj Sprung, Toby N. Weingarten, Todd A. Kellogg, Yvette N. Martin, Travis J. McKenzie, Sorin J. Brull, J. Ross Renew, Harish Ramakrishna, Ana Fernandez-Bustamante, Konstantin Balonov, Harris R. Baig, Aalok Kacha, Juan C. Pedemonte, Fernando Altermatt, Marcia A. Corvetto, Sebastian Paredes, Javiera Carmona, Augusto Rolle, Elke Bos, Charlotte Beurskens, B. Veering, Harry Zonneveldt, Christa Boer, Marc Godfried, Bram Thiel, Barbara Kabon, Christian Reiterer, Jaume Canet, Raquel Tolós, Mar Sendra, Miriam González, Noemí Gómez, Carlos Ferrando, Tania Socorro, Ana Izquierdo, Marina Soro, Manuel Granell Gil, María José Hernández Cádiz, Elena Biosca Pérez, Alejandro Suarez-de-la-Rica, Mercedes Lopez-Martinez, Iván Huercio, Emilio Maseda, Julio Yagüe, Alba Cebrian Moreno, Eva Rivas, Manuel Lopez-Baamonde, Hamed Elgendy, Mohamed Sayedalahl, Abdul Razak SIibai, Aysen Yavru, Nukhet Sivrikoz, Meltem Karadeniz, Pelin Corman Dincer, Hilmi Omer Ayanoglu, Gulbin Tore Altun, Ayse Duygu Kavas, Bora Dinc, Bahar Kuvaki, Sule Ozbilgin, Dilek Erdogan, Ceren Koksal, Suheyla Abitagaglu, Caterina Aurilio, Pasquale Sansone, Caterina Maria Pace, Valerio Donatiello, Silvana Mattera, Palange Nazareno, Salvatore Di Colandrea, Savino Spadaro, Carlo Alberto Volta, Riccardo Ragazzi, Stefano Ciardo, Luca Gobbi, Paolo Severgnini, Alessandro Bacuzzi, Elisa Brugnoni, Angelo Gratarola, Camilla Micalizzi, Francesca Simonassi, Patrizia Malerbi, Adrea Carboni, Marc-Joseph Licker, Alexander Dullenkopf, Nicolai Goettel, Visnja Nesek Adam, Maja Karaman Ilić, Vlasta Klaric, Bibiana Vitkovic, Morena Milic, Zupcic Miro, Luc De Baerdemaeker, Stefan De Hert, Bjorn Heyse, Jurgen Van Limmen, Yves Van Nieuwenhove, Els Mertens, Arne Neyrinck, Jan Mulier, David Kahn, Daniela Godoroja, Martin Martin-Loeches, Sergiy Vorotyntsev, Valentyna Fronchko, Idit Matot, Or Goren, Lilach Zac, Thomasz Gaszynski, Jon Laffey, Gary Mills, Pramod Nalwaya, Mark Mac Gregor, Jonathan Paddle, Packianathaswamy Balaji, Francesca Rubulotta, Afeez Adebesin, Mike Margarson, Simon Davies, Desikan Rangarajan, Christopher Newell, Mirjana Shosholcheva, Fotios Papaspyros, Vasiliki Skandalou, Paula Dzurňáková, and the PROtective VEntilation Network (PROVEnet), on behalf of the Clinical Trial Network of the European Society of Anaesthesiology (ESA)

**Affiliations:** 1 Pulmonary Engineering Group, Department of Anesthesiology and Intensive Care Medicine, University Hospital Carl Gustav Carus, Dresden, Germany; 2 Aneszteziológiai és Intenzív Terápiás Klinika, Semmelweis Egyetem, Budapest, Hungary; 3 Department of Anesthesiology, Hospital Universitari Germans Trias i Pujol, Badalona, Spain; 4 Department of Anesthesiology and Intensive Care Medicine, University of Foggia, Foggia, Italy; 5 Department of Anesthesiology, Ghent University Hospital, Ghent, Belgium; 6 Department of Biopathology and Medical Biotechnologies, Policlinico “P. Giaccone”, Palermo, Italy; 7 Section of Clinical Physiology, Department of Medical Sciences, University Hospital, Uppsala, Sweden; 8 Department of Anesthesiology, Academic Medical Center, University of Amsterdam, Amsterdam, The Netherlands; 9 Laboratory of Experimental Intensive Care and Anesthesiology (L·E·I·C·A), Academic Medical Center, University of Amsterdam, Amsterdam, The Netherlands; 10 Division of Cardiac Surgery, Medical University of Vienna, Vienna, Austria; 11 Division of Thoracic Surgery, Medical University of Vienna, Vienna, Austria; 12 Division of Vascular Surgery, Medical University of Vienna, Vienna, Austria; 13 Department of Anesthesia, Intensive Care and Pain Medicine, Medical University of Vienna, Vienna, Austria; 14 Department of Critical Care Medicine and Anesthesiology (SAR B), Saint Eloi University Hospital, Montpellier, France; 15 Critical Care Medicine Program, Department of Anesthesia, Saint Michael’s Hospital, Toronto, ON Canada; 16 Department of Anesthesia, University of Toronto, Toronto, ON Canada; 17 Department of Physiology, University of Toronto, Toronto, ON Canada; 18 Interdepartmental Division of Critical Care Medicine, University of Toronto, Toronto, ON Canada; 19 Department of Anesthesiology, Pharmacology and Intensive Care, University Hospital Geneva, Geneva, Switzerland; 20 Department of Anesthesia, Intensive Care and Pain Medicine, Medical University of Vienna, Vienna, Austria; 21 Department of Anesthesiology and Critical Care, Tel Aviv Medical Center, Sackler School of Medicine, Tel Aviv University, Tel Aviv, Israel; 22 Center for Evidence-based Healthcare, University Hospital and Medical Faculty Carl Gustav Carus, Technical University Dresden, Dresden, Germany; 23 Operating Services, Critical Care and Anaesthesia (OSCCA), Sheffield Teaching Hospitals and University of Sheffield, Sheffield, UK; 24 Department of Anesthesiology, AZ Sint Jan Brugge-Oostende AV, Brugge, Belgium; 25 Department of Anesthesiology and Intensive Care Medicine, University of Bonn, Bonn, Germany; 26 Department of Anesthesiology, University of Aachen, Aachen, Germany; 27 Department of Anesthesiology and Intensive Care Medicine, Istanbul Medical Faculty, University of Istanbul, Istanbul, Turkey; 28 Department of Critical Care Medicine, Hospital Israelita Albert Einstein, Faculdade de Medicina do ABC, São Paulo, Brazil; 29 Program of Post-Graduation, Research and Innovation, Faculdade de Medicina do ABC, São Paulo, Brazil; 30 Department of Biotechnology and Sciences of Life, University of Insubria, ASST dei Sette Laghi, Ospedale di Cricolo e Fondazione Macchi, Varese, Italy; 31 Department of Anesthesiology, Mayo Clinic, Rochester, MN USA; 32 Department of Anesthesia, Critical Care and Pain Medicine, Massachusetts General Hospital, Harvard Medical School, Boston, MA USA; 33 Department of Anesthesiology and Intensive Care Medicine, University of Leipzig, Leipzig, Germany; 34 Department of Intensive Care, Academic Medical Center, University of Amsterdam, Amsterdam, The Netherlands; 35 Department of Surgical Sciences and Integrated Diagnostics, IRCCS AOU San Martino – IST, University of Genoa, Genoa, Italy

**Keywords:** Mechanical ventilation, Positive end-expiratory pressure, Recruitment maneuver, Obesity, Postoperative pulmonary complication

## Abstract

**Background:**

Postoperative pulmonary complications (PPCs) increase the morbidity and mortality of surgery in obese patients. High levels of positive end-expiratory pressure (PEEP) with lung recruitment maneuvers may improve intraoperative respiratory function, but they can also compromise hemodynamics, and the effects on PPCs are uncertain. We hypothesized that intraoperative mechanical ventilation using high PEEP with periodic recruitment maneuvers, as compared with low PEEP without recruitment maneuvers, prevents PPCs in obese patients.

**Methods/design:**

The PRotective Ventilation with Higher versus Lower PEEP during General Anesthesia for Surgery in OBESE Patients (PROBESE) study is a multicenter, two-arm, international randomized controlled trial. In total, 2013 obese patients with body mass index ≥35 kg/m^2^ scheduled for at least 2 h of surgery under general anesthesia and at intermediate to high risk for PPCs will be included. Patients are ventilated intraoperatively with a low tidal volume of 7 ml/kg (predicted body weight) and randomly assigned to PEEP of 12 cmH_2_O with lung recruitment maneuvers (high PEEP) or PEEP of 4 cmH_2_O without recruitment maneuvers (low PEEP). The occurrence of PPCs will be recorded as collapsed composite of single adverse pulmonary events and represents the primary endpoint.

**Discussion:**

To our knowledge, the PROBESE trial is the first multicenter, international randomized controlled trial to compare the effects of two different levels of intraoperative PEEP during protective low tidal volume ventilation on PPCs in obese patients. The results of the PROBESE trial will support anesthesiologists in their decision to choose a certain PEEP level during general anesthesia for surgery in obese patients in an attempt to prevent PPCs.

**Trial registration:**

ClinicalTrials.gov identifier: NCT02148692. Registered on 23 May 2014; last updated 7 June 2016.

**Electronic supplementary material:**

The online version of this article (doi:10.1186/s13063-017-1929-0) contains supplementary material, which is available to authorized users.

## Background

It is well established that postoperative pulmonary complications (PPCs), especially postoperative respiratory failure, add greatly to perioperative morbidity and mortality, as well as to postoperative length of hospital stay [1–3]. Several independent risk factors for the development of PPCs have been identified, ranging from a patient’s health conditions to surgical approaches and anesthetic management [4]. Considering that more than 234 million surgical procedures are performed worldwide per year [5], a reduction of the rate of PPCs might have an important impact on global morbidity and mortality and reduce health system costs. Anesthetists could importantly contribute to preventing such respiratory complications, such as through intraoperative mechanical ventilation strategies expected to affect PPCs beyond preoperative patient status optimization and selection of operative methods to minimize surgical trauma. In fact, mechanical ventilation during general anesthesia has the potential to cause harm to previously noninjured lungs [4].

Authors of an individual patient data meta-analysis showed that intraoperative lung-protective mechanical ventilation using lower tidal volume (V_T_) in the range of 6 to 8 ml/kg of predicted body weight (PBW), with or without higher levels of positive end-expiratory pressure (PEEP) and with or without lung recruitment maneuvers (RMs), reduced the incidence of PPCs [2]. More recently, authors of another individual patient data meta-analysis identified the use of intraoperative low V_T_ as a protective measure against PPCs, whereas the role of PEEP was unclear [6]. In fact, in a randomized controlled trial with patients with body mass index (BMI) <40 kg/m^2^ undergoing open abdominal surgery, higher PEEP with RMs did not prevent PPCs compared with lower PEEP without RMs [7].

BMI is an important determinant of respiratory function before and during anesthesia in obese patients [8–10]. In these patients, lung function impairment can manifest as (1) reduced lung volume with increased atelectasis and/or small airway closure; (2) derangements in respiratory system, lung, and chest wall compliance as well as increased resistance; and (3) moderate to severe hypoxemia. These physiological alterations are more marked in obese patients with hypercapnia or obstructive sleep apnea (OSA). To reduce such complications, PEEP levels should theoretically be set higher in obese than in nonobese patients. However, there is as yet no clinical evidence supporting such an approach. An observational study conducted in 28 centers in France revealed that most patients undergoing general surgery, including obese patients, were ventilated with low PEEP (≤4 cmH_2_O) or even without PEEP, even though average PEEP was higher in obese than in nonobese patients [11]. In fact, current recommendations on the use of PEEP and RMs [4, 12] are derived from trials that included mainly patients with BMI <35 kg/m^2^ and therefore cannot be extrapolated to obese patients.

The aim of the PRotective Ventilation with Higher versus Lower PEEP during General Anesthesia for Surgery in OBESE Patients (PROBESE) trial is to compare the effects of two intraoperative mechanical ventilation strategies on PPCs, extrapulmonary postoperative complications (PEPCs), and length of hospital stay, as well as intraoperative lung function and hemodynamics, in surgical patients with BMI ≥35 kg/m^2^. We hypothesized that intraoperative mechanical ventilation using high PEEP with periodic RMs, as compared with low PEEP without RMs, prevents PPCs in obese patients.

## Methods/design

### Objectives and design

**Fig. 1:**
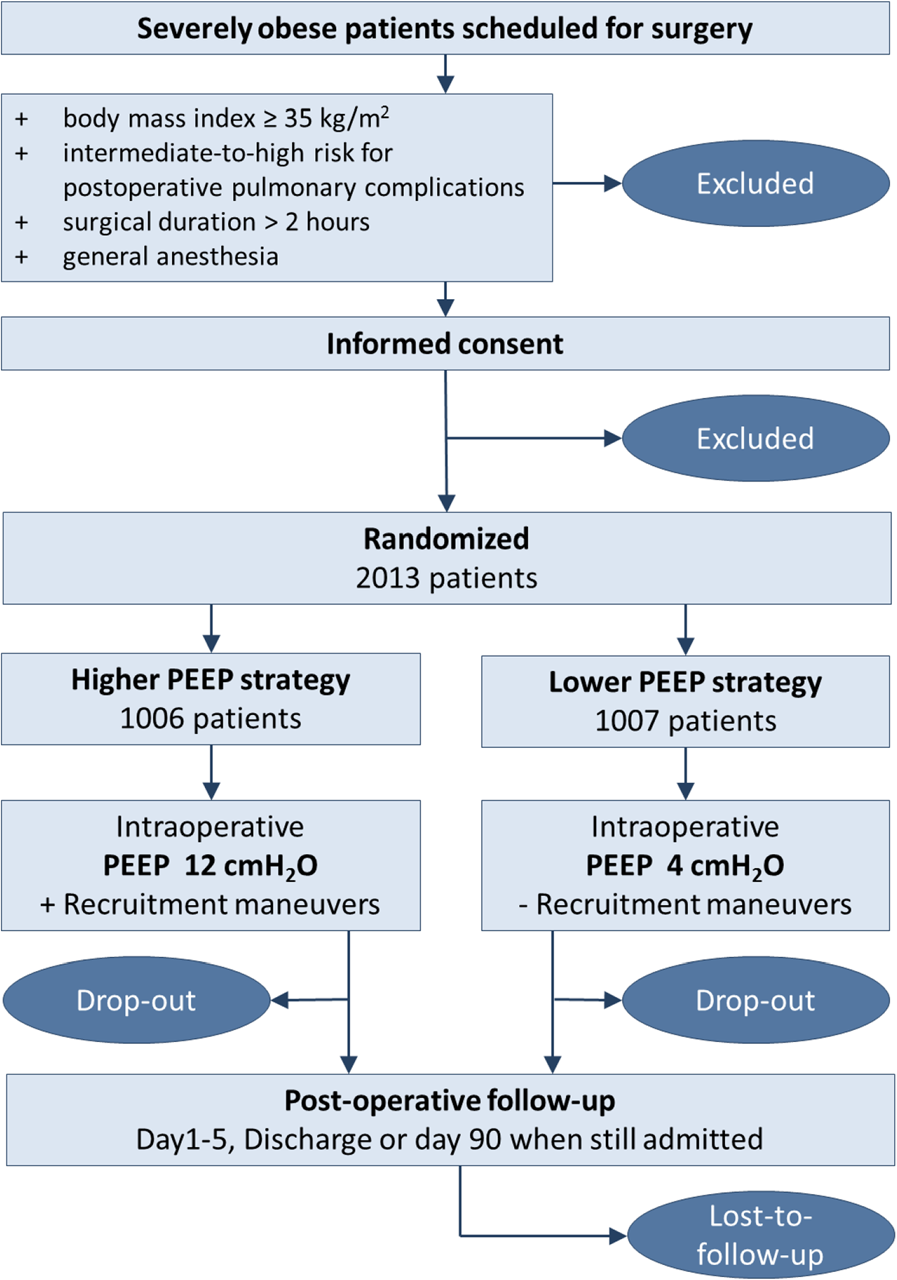
Consolidated Standards of Reporting Trials (CONSORT) diagram for the PRotective Ventilation with Higher versus Lower PEEP during General Anesthesia for Surgery in OBESE Patients (PROBESE) trial. *PEEP* Positive end-expiratory pressure

PROBESE is a prospective international multicenter, randomized, controlled, two-arm trial initiated by investigators of the PROtective VEntilation NETwork (www.provenet.eu). In total, 2013 patients will be randomly assigned to one of two different intraoperative mechanical ventilation strategies (*see* Consolidated Standards of Reporting Trials [CONSORT] diagram, Fig. 1).

The PROBESE trial will test the hypothesis that, during an intraoperative lung-protective mechanical ventilation strategy with low V_T_ s, higher levels of PEEP and RMs, as compared with ventilation with lower levels of PEEP without RMs, reduce PPCs in obese patients at intermediate to severe risk for PPCs. After starting the trial, recalculation of the sample size was conducted upon a recommendation of the Data and Safety Monitoring Board (DSMB) (*see* “Sample size calculations” section). There were changes in neither the study protocol (version 2.5; February 2016, Additional file 1) nor any of the endpoints. A complete checklist of recommended items to address in a clinical trial protocol and related documents according to the "Standard Protocol Items: Recommendations for Interventional Trials (SPIRIT) 2013 is provided in Additional file 2.

### Study population

Investigators screen for obese patients with BMI ≥35 kg/m^2^ scheduled for surgery under general anesthesia. Patients are eligible if the expected duration of surgery (from incision to closure) exceeds 2 h and if they are at intermediate to high risk for PPCs. The number of patients meeting these enrollment criteria will be recorded.

To identify patients at risk for PPCs, the Assess Respiratory Risk in Surgical Patients in Catalonia (ARISCAT) score is used [13]. This score predicts individual preoperative risk for PPCs using seven independent predictors, four of which are patient-related and three of which are surgery-related (Table 1). An ARISCAT risk score ≥26 is associated with an intermediate to high risk for PPCs.

**Table 1:** Assess Respiratory Risk in Surgical Patients in Catalonia scores

	Multivariate analysis, OR (95% CI), *n* = 1624	β Coefficient	Risk score^a^
Age, years			
≤50	1		
51–80	1.4 (0.6–3.3)	0.331	3
>80	5.1 (1.9–13.3)	1.619	16
Preoperative SpO_2_, %			
≥96	1		
91–95	2.2 (1.2–4.2)	0.802	8
≤90	10.7 (4.1–28.1)	2.375	24
Respiratory infection in the last month	5.5 (2.6–11.5)	1.698	17
Preoperative anemia (≤10 g/dl)	3.0 (1.4–6.5)	1.105	11
Surgical incision			
Peripheral	1		
Upper abdominal	4.4 (2.3–8.5)	1.480	15
Intrathoracic	11.4 (4.9–26.0)	2.431	24
Duration of surgery, h			
≤2	1		
>2–3	4.9 (2.4–10.1)	1.593	16
>3	9.7 (4.7–19.9)	2.268	23
Emergency procedure	2.2 (1.04–4.5)	0.768	8

*SpO*_*2*_ Peripheral oxyhemoglobin saturation measured by pulse oximetry breathing air in supine position

Independent predictors of risk for development of postoperative pulmonary complications as described by Canet et al. [13] (ARISCAT score). A risk score ≥26 predicts an intermediate to high risk for postoperative pulmonary complications)

^a^The simplified risk score is the sum of each logistic regression coefficient multiplied by 10, after rounding off its value

Patients are excluded from participation if they are aged <18 years, have undergone any kind of previous lung surgery, have been invasively mechanically ventilated for longer than 30 minutes within the last 30 days before surgery, or have received recent immunosuppressive medication (chemotherapy or radiation therapy up to 2 months prior to surgery). Further exclusion criteria comprise neurosurgical procedures and cardiac surgery, need for one-lung ventilation or planned reintubation following surgery, need for intraoperative prone or lateral decubitus position, enrollment in another interventional study, or refusal to give written informed consent. Additionally, patients showing at least one the following medical conditions are excluded: pregnancy (excluded by anamnesis and/or laboratory analysis), persistent hemodynamic instability or intractable shock (considered hemodynamically unsuitable for the study by the patient’s managing physician), history of severe chronic obstructive pulmonary disease (COPD; defined as noninvasive ventilation and/or oxygen therapy at home or repeated systemic corticosteroid therapy for acute exacerbations of COPD), severe cardiac disease (defined as New York Heart Association class III or IV, acute coronary syndrome, or persistent ventricular tachyarrhythmia), concurrent acute respiratory distress syndrome (ARDS) expected to require prolonged postoperative mechanical ventilation, severe pulmonary arterial hypertension (defined as systolic pulmonary arterial pressure >40 mmHg), intracranial injury, or tumor or neuromuscular disease.

### Intervention

Patients undergo intraoperative lung-protective mechanical ventilation with protective low V_T_ of 7 ml/kg (PBW), and they are randomly assigned to a PEEP level of 12 cmH_2_O with planned lung RMs performed after intubation, hourly thereafter and preceding extubation (“high PEEP”), or a level of PEEP of 4 cmH_2_O without planned RMs (“low PEEP”). PEEP levels are to be maintained throughout the whole period of intraoperative mechanical ventilation.

### Minimization of bias

The allocation sequence is computer-generated (nQuery 4; Statsols, Boston, MA, USA) using permuted blocks of different block sizes, with a maximum block size of 8. Allocation is stratified per center with an allocation ratio of 1:1 for each group. The process of sequence generation and storage is managed by an independent information technology expert not involved in patient care. Randomization is then performed patient-by-patient using a web interface as an integral part of the online case report form (CRF, Additional file 3; *see* “Handling of data” section).

At each study site, at least two investigators are involved with the study. One investigator is involved with the intraoperative mechanical ventilation strategy and performs the interventions defined in the protocol. A second investigator, who is blinded to randomization, performs postoperative visits and assessment of primary and secondary endpoints.

### Standard procedures

To avoid interference with the trial intervention, routine elements of perioperative anesthetic care (including general anesthesia, postoperative pain management, physiotherapeutic procedures, and fluid management) are performed according to each center’s specific expertise and clinical routine. The following approaches are suggested (not mandatory) for anesthetic management:

1. Use of inhalational isoflurane, desflurane, or sevoflurane; intravenous propofol, remifentanil, or sufentanil; and cisatracurium, atracurium, vecuronium, or rocuronium as required;
2. Use of a balanced solution of prostigmine, or neostigmine and atropine or glycopyrrolate, for reversal of muscle relaxation, guided by neuromuscular function monitoring;
3. Performing postoperative pain management to achieve a visual analogue scale (VAS) pain score <3, and regional or neuraxial analgesia should be used whenever indicated;
4. Use of physiotherapy by early mobilization, deep breathing exercises with and without incentive spirometry, and stimulation of cough in the postoperative period; (5) avoidance of hypo- and hypervolemia;
5. Use of invasive measurement of arterial blood pressure whenever indicated; and
6. Use of appropriate prophylactic antibiotic drugs whenever indicated.

Data on the procedures applied will be collected in detail and analyzed.

In addition, the study protocol stresses that routine intraoperative monitoring should include measurements of noninvasive blood pressure, pulse oximetry, end-tidal carbon dioxide fraction, and electrocardiography. Every patient should receive at least one peripheral venous line to allow adequate fluid resuscitation during the study period. Nasogastric tubes, urinary bladder catheters, and/or other intravenous catheters, as well as other, more invasive monitoring, may be used according to local practice and/or guidelines. Other procedures should follow the Safe Surgery Checklist of the World Health Organization (WHO) (www.who.int/patientsafety/safesurgery/en/index.html).

### Mechanical ventilation

Mechanical ventilation is performed with anesthesia ventilators in use at each individual center participating in the study. Patients undergo volume-controlled mechanical ventilation with the lowest possible fraction of inspired oxygen (FiO_2_; ≥0.4) to maintain a peripheral oxyhemoglobin saturation measured by pulse oximetry (SpO_2_) >92%, an inspiratory-to-expiratory ratio (I:E) of 1:2, and a respiratory rate adjusted to normocapnia (end-tidal carbon dioxide partial pressure between 35 and 45 mmHg). It is left to the discretion of the attending anesthesiologist whether to use a higher FiO_2_.

V_T_ is set to 7 ml/kg (PBW). The PBW is calculated according to a predefined formula: 50 + 0.91 × (centimeters of height − 152.4) for men and 45.5 + 0.91 × (centimeters of height − 152.4) for women [14, 15]. V_T_ throughout this protocol refers to the actual inspired V_T_ in the ventilator circuit. PEEP is set according to the randomized intervention to 4 vs. 12 cmH_2_O and is modified only as part of the rescue strategy (in case of desaturation; *see* below) or at the discretion of the treating physician.

### Planned and unplanned recruitment maneuvers

**Fig. 2:**
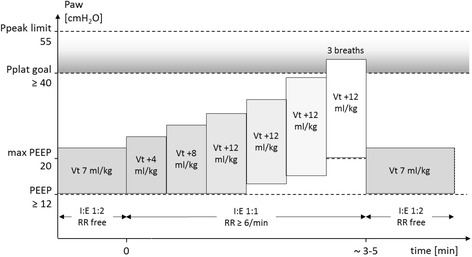
Lung recruitment maneuver protocol. *Ppeak* Peak airway pressure, *Pplat* Plateau airway pressure, *PEEP* Positive end-expiratory pressure, *Vt* Tidal volume normalized for predicted body weight, *RR* Respiratory rate, *I:E* Ratio between inspiratory and expiratory time

The RM, as part of the high-PEEP strategy, is performed directly after induction of anesthesia, after any disconnection from the mechanical ventilator, every 1 h during surgery, and before extubation, in a hemodynamically stable situation as judged by the anesthesiologist. RMs may also be performed as part of a rescue strategy in the low-PEEP group. To obtain standardization among centers, RMs will be performed in volume-controlled ventilation mode, as shown schematically in Fig. 2, and according to the following steps:

1. Set peak inspiratory pressure limit to 55 cmH_2_O.
2. Set V_T_ to 7 ml/kg PBW and respiratory rate to ≥6 breaths/minute while PEEP is 12 cmH_2_O (or higher if during rescue; *see* below).
3. Set I:E to 1:1.
4. Increase V_T_ in steps of 4 ml/kg PBW until plateau pressure reaches 40–50 cmH_2_O.
5. If the maximum V_T_ allowed by the anesthesia ventilator is achieved and the plateau pressure is <40 cmH_2_O, increase the PEEP as needed but only to a maximum of 20 cmH_2_O.
6. Allow the patient three breaths while maintaining plateau pressure of 40–50 cmH_2_O.
7. Set respiratory rate, I:E, inspiratory pause, and V_T_ back to prerecruitment values while maintaining PEEP at 12 cmH_2_O (or higher if during rescue).

### Rescue strategies for intraoperative hypoxemia

If SpO_2_ ≤92% develops, increase in airway resistance, presence of intrinsic PEEP, hemodynamic impairment, and ventilator malfunction must be excluded before group-specific stepwise rescue strategies can be applied (Table 2).

**Table 2:** Rescue strategies for intraoperative hypoxemia

	Lower PEEP			Higher PEEP		
Step	FiO_2_	PEEP (cmH_2_O)		FiO_2_	PEEP (cmH_2_O)	
1	0.5	4		0.4	14	(+RM)
2	0.6	4		0.4	16	(+RM)
3	0.7	4		0.4	18	(+RM)
4	0.8	4		0.5	18	
5	0.9	4		0.6	18	
6	1.0	4		0.7	18	
7	1.0	5		0.8	18	
8	1.0	6		0.9	18	
9	1.0	7	(+RM)	1.0	18	
10				1.0	20	(+RM)

*Abbreviations: FiO*_*2*_ Fraction of inspired oxygen, *PEEP* Positive end-expiratory pressure, *RM* Recruitment maneuver

If intraoperative hypoxemia, defined as oxygen saturation ≤92%, develops, sequences of interventions will be used according to group assignment

In patients receiving lower PEEP levels, rescue consists primarily of an increase in FiO_2_, whereas elevation of PEEP levels is restricted to more severe cases of hypoxemia. In the higher PEEP group, the rescue strategy consists primarily of increase of PEEP before FiO_2_ is to be increased. At any rescue step, the treating physician may consider reducing PEEP if SpO_2_ deteriorates further in an otherwise hemodynamically stable patient.

### Protocol deviation

Anesthesiologists may deviate from the ventilation protocol at any time if concerns about patient safety arise or upon the surgeon’s request. PEEP may be modified according to the anesthesiologist’s judgment in the presence of any of the following clinical situations:

1. Decrease in systolic arterial pressure <90 mmHg and unresponsive to fluids and/or vasoactive drugs
2. Need for a dosage of vasoactive drugs at the tolerance limit
3. New arrhythmias unresponsive to the treatment suggested by the Advanced Cardiac Life Support Guidelines [16]
4. Blood loss requiring massive transfusion (defined as replacement of >100% blood volume in 24 h or >50% of blood volume in 4 h to maintain hematocrit >21% [hemoglobin >7 mg/dl])
5. Any life-threatening surgical complication that might benefit from changes in PEEP

Details about any protocol deviation will be prospectively collected and analyzed.

### Study endpoints

The primary endpoint of PROBESE is a collapsed composite of all PPCs developing within the first 5 postoperative days. With this approach, each complication is weighted equally. Patients who develop a least one complication are considered as meeting the primary endpoint.

PPCs are defined as follows:

1. Mild respiratory failure (partial pressure of arterial oxygen [PaO_2_] <60 mmHg or SpO_2_ <90% breathing at least 10 minutes of room air but responding to supplemental oxygen of 2 L/minute, excluding hypoventilation);
2. Moderate respiratory failure (PaO_2_ <60 mmHg or SpO_2_ <90% breathing ≥10 minutes of room air but responding only to supplemental oxygen >2 L/minute, excluding hypoventilation);
3. Severe respiratory failure (need for noninvasive or invasive mechanical ventilation, excluding hypoventilation resulting from use of sedative agents);
4. ARDS (according to the Berlin Definition [17]);
5. Bronchospasm (newly detected expiratory wheezing treated with bronchodilators);
6. New pulmonary infiltrates (chest x-ray demonstrating new monolateral or bilateral infiltrate without other clinical signs);
7. Pulmonary infection (new or progressive radiographic infiltrate plus at least two of the following: antibiotic treatment, tympanic temperature >38 °C, leukocytosis or leukopenia [white blood cell count <4000 cells/mm^3^ or >12,000 cells/mm^3^], and/or purulent secretions);
8. Aspiration pneumonitis (respiratory failure after the inhalation of regurgitated gastric contents);
9. Pleural effusion (chest x-ray demonstrating blunting of the costophrenic angle, loss of the sharp silhouette of the ipsilateral hemidiaphragm in upright position, evidence of displacement of adjacent anatomical structures, or [in supine position] a hazy opacity in one hemithorax with preserved vascular shadows);
10. Atelectasis (lung opacification with shift of the mediastinum, hilum, or hemidiaphragm toward the affected area, as well as compensatory overinflation in the adjacent nonatelectatic lung);
11. Cardiopulmonary edema (clinical signs of congestion, including dyspnea, edema, rales, and jugular venous distention, with chest x-ray demonstrating increase in vascular markings and diffuse alveolar interstitial infiltrates); and
12. Pneumothorax (air in the pleural space with no vascular bed surrounding the visceral pleura).

Secondary clinical endpoints include the following:

1. Collapsed severe PPC composite, defined as any of the above-mentioned adverse pulmonary events, except mild respiratory failure;
2. Intraoperative adverse events (AEs), such as hypoxemia (defined as SpO_2_ ≤92%), hypotension (defined as systolic blood pressure <90 mmHg), and bradycardia (defined as heart rate <50 beats/minute);
3. Unexpected need for intensive care unit (ICU) admission or ICU readmission;
4. Hospital-free days at follow-up day 90;
5. Postoperative wound healing; and
6. Postoperative extrapulmonary complications (PEPCs).

PEPCs include systemic inflammatory response syndrome, sepsis, severe sepsis, and septic shock (all according to consensus criteria [18]); extrapulmonary infection (wound infection or any other infection); coma (Glasgow Coma Scale score <8 in the absence of therapeutic coma or sedation); acute myocardial infarction (according to universal definition of myocardial infarction [19]); acute renal failure (according to the risk, injury, failure, loss, end-stage kidney disease [RIFLE] classification system [20]); disseminated intravascular coagulation (DIC) (according to the International Society of Thrombosis and Hemostasis diagnostic scoring system for DIC [21]); gastrointestinal failure (GIF) (defined according to the GIF score [22]); and hepatic failure (defined as the ratio of total bilirubin on postoperative day 5 to postoperative day 1 >1.7 and ratio of international normalized ratio [INR] on postoperative day 5 to postoperative day 1 >1.0, or new presence of hepatic encephalopathy and coagulopathy [INR >1.5] within 8 weeks after initial signs of liver injury [e.g., jaundice] without evidence of chronic liver disease) (adapted from Du et al. [23] and Wlodzimirow et al. [24]).

At the discretion of participating centers, blood and urine samples are collected preoperatively as well as directly postoperatively and on postoperative day 5. Samples will be analyzed centrally for systemic markers of inflammation and coagulation (including but not limited to interleukins 6 and 8, thrombin-antithrombin, protein C, and plasminogen activator inhibitor-1) as well as systemic markers of injury to the lungs (including but not limited to plasma E-cadherin, soluble receptor for advanced glycation end products, and surfactant proteins A and D) and to distal organs, including renal injury (including but not limited to plasma/urine neutrophil gelatinase-associated lipocalin and cystatin C). The standard operating procedure for collecting and processing biomarkers in plasma and urine is available in the online supplement (Additional files 4 and 5, respectively).

### Study visits and data collection

**Fig. 3:**
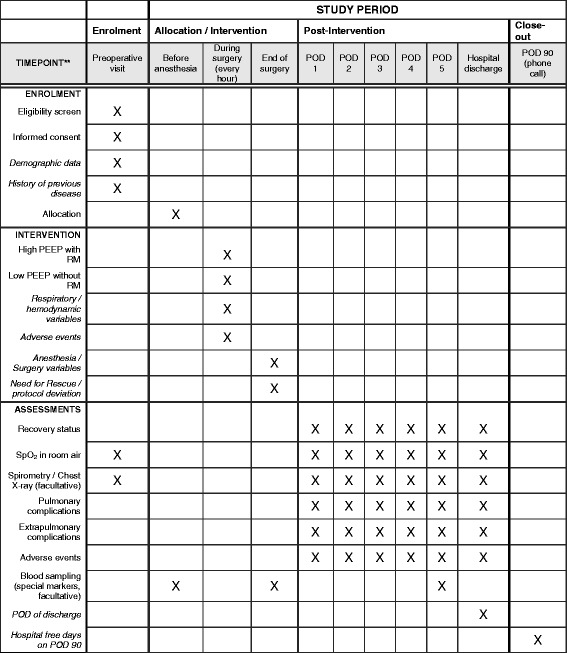
Schedule of enrollment, interventions, and assessments. *POD* Postoperative day, *PEEP* Positive end-expiratory airway pressure, *RM* Recruitment maneuver, *SpO*_*2*_ Peripheral oxyhemoglobin saturation measured by pulse oximetry

Patients are visited preoperatively, intraoperatively, daily between postoperative days 1 and 5, and at discharge. On postoperative day 90, patients are contacted by phone (Fig. 3). A complete participant time line, including all variables as well as interventions, is available in Additional files 1 and 2.

During the preoperative visit, eligible patients meeting none of the exclusion criteria are asked by physicians to provide written informed consent. (Model consent form and information to study patients). Baseline variables are collected, including sex; age; height; weight; BMI; waist/hip ratio according to WHO guidelines; physical status according to the American Society of Anesthesiologists; functional status according to Cumulated Ambulation Score [25]; cardiovascular status (heart failure according to the New York Heart Association classification, coronary heart disease according to Canadian Cardiovascular Society, atrial flutter/fibrillation, arterial hypertension); pulmonary status (COPD, including steroids and/or inhalation therapy use, respiratory infection within the last month, use of noninvasive ventilatory support); history of OSA, including apnea-hypopnea index or STOP-Bang score (snoring, tired, observed stopped breathing or choking/gasping, pressure, body mass index >35 kg/m^2^, age >50 years, large neck size, and male sex) [26] in patients without diagnosis of OSA); metabolic status (diabetes mellitus, including data on treatment); history of active cancer; smoking status; alcohol status; gastroesophageal reflux; oral medication (e.g., use of antibiotics, statins, aspirin); preoperative organ function (SpO_2_ in beach chair position breathing room air; if possible, SpO_2_ in supine position breathing room air; if possible, so-called oxygen stress test, with this measurement left to the discretion of each center); respiratory rate; heart rate; mean arterial pressure; body temperature; airway secretion, including data on purulence, VAS score (1–10) for dyspnea, chest pain, and abdominal rest; and incident pain. Preoperative nonmandatory measurements include spirometry (forced ventilatory capacity, forced expiratory volume in 1 second), chest x-ray (assessed for infiltrates, pleural effusion, atelectasis, pneumothorax, and cardiopulmonary edema), and routine laboratory tests (including hemoglobin, white blood cell count, platelet count, prothrombin time, partial thromboplastin time, creatinine, blood urea nitrogen, alanine aminotransferase, aspartate amino transferase, bilirubin).

During the intraoperative visit, both surgery- and anesthesia-related data are recorded, including duration of surgery (from incision to closure), transfusion of blood products within 6 h before surgery, priority and type of surgery, wound classification, patient positioning during operation, duration of anesthesia (from intubation to extubation or exit from operating room if on mechanical ventilation), anesthetic procedure details, drugs and fluids administered during anesthesia (e.g., anesthetics, vasoactive drugs, transfusion). Ventilator settings, hemodynamics, need for rescue strategy, and AEs are recorded at anesthesia induction and hourly thereafter (in the higher PEEP group, before performing the RM) as well as during the plateau phase of the RM.

Clinical data, including actual organ function and the presence of PPCs and PEPCs, are scored during postoperative visits on a daily basis. Nonmandatory measures include chest x-ray, spirometry, and routine laboratory tests. Patients will be visited until discharge. On postoperative day 90, the sum of hospital-free days is recorded. Day 90 is defined as the last day of follow-up; accordingly, patients still admitted to the hospital will be last visited on that day.

### Study dropouts

Because participation in the trial is voluntary, a subject has the right to withdraw consent to participate in the study at any time for any reason without any consequences for further medical treatment. Furthermore, investigators have the right to terminate participation of any subject at any time if the investigator deems it in the participant’s best interest. The reasons and circumstances for study discontinuation will be documented in the CRF. Primarily, all data will be analyzed according to the intention-to-treat (ITT) principle. Secondarily, data will be analyzed as per protocol.

### Handling of data

Patient data are collected in pseudonymous form using a patient identification number of six digits. The first three digits correspond to the site identifier, and the remaining three digits correspond to the patient inclusion number at the respective site. Study data are collected and managed using Research Electronic Data Capture (REDCap™; a web-based system used during PROBESE as electronic case report form) electronic data capture tools hosted at the Coordinating Center for Clinical Trials of the University of Dresden, Germany [27]. REDCap is a Secure Sockets Layer (SSL)-encrypted, password-protected, web-based application designed to support data capture for research studies. Full access to the final trial dataset will be granted to selected investigators only (MGdA, TB, and JSc). If a substudy is approved by the steering committee, access to data related only to the substudy will be granted to the respective principal investigator.

### Sample size calculations

Sample size calculation was based on our primary hypothesis that, in obese patients ventilated intraoperatively with protective low V_T_, high PEEP leads to lower incidence of PPCs than lower PEEP. Effect sizes were derived from data collected during the ARISCAT study [13] and a single-center, relatively small study in which researchers reported the effects of intraoperative higher PEEP and RMs on the incidence of postoperative desaturation, chest infection, and bronchospasm in obese patients who underwent laparoscopic bariatric surgery [28].

**Fig. 4:**
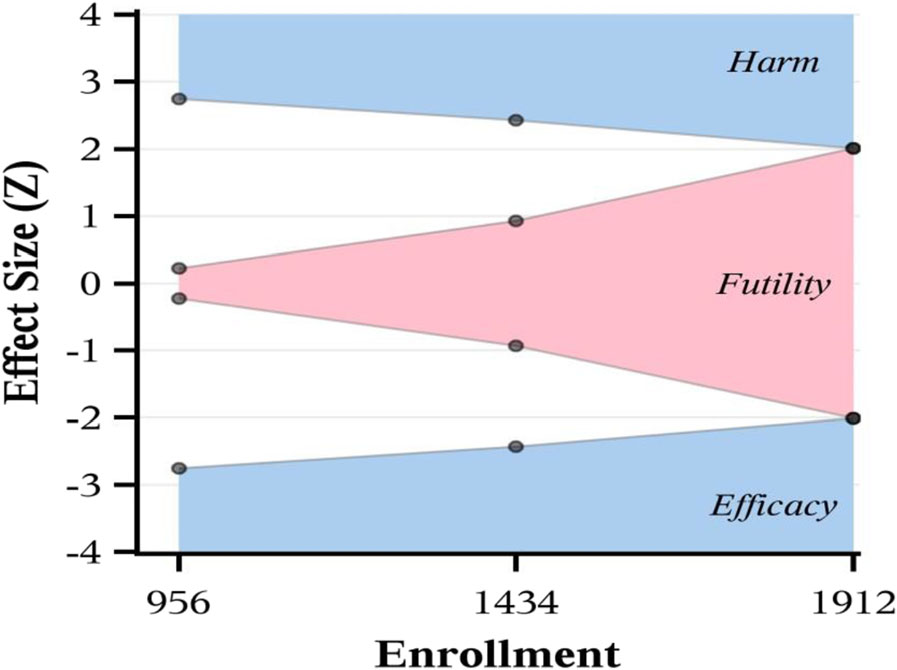
Effect size (Z) according to enrollment of patients in the PRotective Ventilation with Higher versus Lower PEEP during General Anesthesia for Surgery in OBESE Patients (PROBESE) trial

Prior to the start of the study, these calculations had indicated that 356 patients would be required per group, assuming a two-sided significance level of 0.05 (α) and a power of 80%, to detect the expected difference in PPCs between the higher-PEEP group of 30% and the lower-PEEP group of 40% (risk ratio of 0.75). However, the sample size was reestimated after data of the first 618 patients revealed that the overall incidence of the collapsed composite outcome was 20% and, thus, lower than initially expected. Also, the adjustment of the sample size took into account the need for interim analyses for efficacy and futility at 50%, 75%, and 100% of the total number of patients, for which a nonbinding group sequential design with γ spending functions (γ = −4 for each of α and β) was used. In total, 1912 patients will be included in the analysis. Assuming a dropout rate of 5%, 2013 patients will be enrolled. Table 3 shows the α and β spent over the trial, z-statistic boundaries for efficacy and futility, and boundary-crossing probabilities under the alternative hypothesis (H1). The corresponding *P* value boundaries for efficacy and futility at the first, second, and final looks, respectively, are *P* ≤ 0.006, *P* ≤ 0.015, and *P* ≤ 0.044, as well as *P* > 0.82, *P* > 0.35, and *P* > 0.044, respectively. Figure 4 displays the z-statistic boundaries for efficacy/harm and futility as a function of accrued sample size. East 6.0 interim monitoring software (Cytel Inc., Cambridge, MA, USA) was used for sample size calculations.

**Table 3:** Z-statistic boundaries and boundary-crossing probabilities

Look	Information fraction	*N*	Cumulative α spent	Cumulative β spent	Z-efficacy	Z-futility	Boundary-crossing probabilities under H1	
							Efficacy	Futility
1	0.5	956	0.006	0.024	≥2.75	<0.225	0.234	0.024
2	0.75	1434	0.018	0.071	≥2.432	<0.929	0.296	0.047
3	1	1912	0.05	0.2	≥2.012	<2.012	0.271	0.129

*Look* Interim analysis, *N* Number of patients, *H1* Hypothesis 1 (group difference exists)

Values were calculated using power = 0.80, α = 0.05, γ spending functions (γ = −4), and expected incidence of postoperative pulmonary complications of 20% (p1) and 15% (p2) in the lower and higher positive end-expiratory pressure groups, respectively

To foster the study and increase the interest of practicing physicians, the steering committee will apply for endorsement of national and international professional societies. The following societies have already given endorsement to the trial: the European Society of Anaesthesiology, the European Society for Perioperative Care of the Obese Patient, the German Society of Anesthesiology and Intensive Care Medicine, and the Italian Society of Anesthesiology and Reanimation.

### Statistical analysis

Exploratory analysis will include mean and SD for normally distributed variables. Non-normally distributed variables will be expressed by their medians and IQRs, and categorical variables will be expressed as count (percent). Parametric or nonparametric tests will be used as appropriate. Categorical variables will be compared with chi-square tests, Fisher’s exact test, or as relative risk, if appropriate. Statistical uncertainty will be expressed by 95% CIs.

Preoperative (baseline) data will be tested for any imbalance. If imbalances are detected (despite 1:1 randomization of a relatively large cohort), those factors will be corrected for using a multiple logistic regression model.

The primary endpoint, namely occurrence of any PPC (collapsed composite endpoint) within the first 5 postoperative days, will be presented as total percentage per group and analyzed as continuous data. Primary and secondary outcome variables describing time to event will be analyzed using a proportional hazards model adjusted for possible baseline imbalances. A linear mixed model with two factors (study group and time) will be used to analyze variables repetitively measured over time.

In case of loss to follow-up or study dropout, those cases will be reported, and ITT as well as per-protocol analyses will be performed. For ITT analysis, data will be processed for all patients in the groups to which they were randomized. The per-protocol analysis will be conducted to assess the primary outcome in cases where there is a considerable proportion of patients who do not receive their randomized intervention or are lost to follow-up. Patients discharged earlier than postoperative day 5 are considered as not experiencing any PPC or PEPC during the out-of-hospital days. In this regard, missing data will be handled by means of the last observation carried forward method.

Given that laparoscopic surgery is common in obese patients, we anticipate that a subgroup analysis of primary and secondary endpoints will be conducted for this type of surgery as well. A further subgroup analysis of patients with obesity class III according to the WHO definition (i.e., BMI ≥40 kg/m^2^) will be performed. Given the importance of driving pressure to determine PPCs [29, 30], a subgroup analysis taking into account cutoff values and changes in PEEP will be conducted.

### Possibility and policy for substudies

Participating centers are allowed to conduct substudies, provided that (1) no interference with the primary protocol occurs; (2) approval by the local institutional review board is obtained; and (3) the steering committee accepts the proposal according to its originality, feasibility, and importance. Currently, substudies with electrical impedance tomography, spirometry, respiratory system mechanics, and preoperative oxygen stress test are under evaluation. Publication of substudies, in any form, is strictly forbidden until the results of the primary study have been published.

### Trial organization

The trial is managed by a team consisting of the chief investigator (MGdA), the trial coordinator (TB), the statisticians (JSc, GM), the informatics technician responsible for the web-based electronic data capture system (Marko Kaeppler), and the monitors (Luigi Vivona, Alice Bergamaschi). A steering committee contributed to the design and revision of the study and will be responsible for interpretation of data and compilation of a resulting manuscript.

Patient data and safety are closely monitored by a DSMB, which is composed of a chairperson (Daniel Sessler) and four other members (Jennifer Hunter, Jeanine Wiener-Kronish, Jean-Louis Vincent, and Andreas Hoeft). All AEs entered into the electronic CRF within prespecified time frames, including severe AEs and suspected unexpected severe adverse reactions, are monitored by an international AE manager (ASN), who provides the DSMB with reports for review. The DSMB further monitors the overall status of the trial (e.g., progress of patient enrollment, general adherence to protocol, and completeness of data entry). Monitoring visits will be conducted as deemed necessary by the DSMB. National coordinators are responsible for administration and communication with local principal investigators, as well as for assistance during trial management and data collection. When submitting the report on the results of the trial for possible publication, sites will be eligible for one collaborative coauthorship plus a further coauthorship for every 12 treated patients with complete datasets.

## Discussion

Despite being lifesaving, mechanical ventilation has the potential to aggravate or even initiate lung injury. In patients with previously injured lungs, especially those with ARDS, mechanical ventilation with low V_T_ [31] and driving pressures [32] has been shown to decrease mortality. Furthermore, in more severe ARDS, high PEEP improves survival [33]. Such effects have been attributed to avoidance of tidal overdistention as well as cyclic collapse and reopening of lung units, which may trigger the inflammatory lung response [34]. Interestingly, low V_T_ with low to moderate PEEP levels has been also reported to facilitate weaning [35] and reduce pulmonary complications [36] in critically ill patients without lung injury. However, the value of protective ventilation in the absence of lung injury has been challenged. In pigs with noninjured lungs, V_T_ as high as 27 ml/kg was not associated with relevant degrees of lung damage [37]. Such an observation might be explained by the fact that, within a certain range, mechanical ventilation does not injure lungs if a previous insult (first hit), such as inflammation, ischemia-reperfusion injury, or factors impairing the homogeneity of ventilation [38], are not present. Surgery itself can trigger a systemic inflammatory response [39], which may prime the lungs for possible harmful effects of mechanical ventilation. In fact, protective intraoperative mechanical ventilation with low V_T_ and PEEP is able to prevent postoperative respiratory failure in patients undergoing abdominal or thoracic surgery, and those who develop this adverse pulmonary event not only have longer stays in the hospital but also a significantly higher risk of death [2]. However, though the role of intraoperative low V_T_ [6] and low driving pressure [29, 30] to decrease the risk of PPCs has been defined, the role of PEEP is more controversial [4, 7, 40, 41].

The decision to address the obese patient population undergoing surgery is based on several aspects. The proportion of obese patients undergoing surgery is higher than in the general population [42]. Treatment of these patients is usually challenging because their needs in terms of perioperative care differ from those of nonobese patients and are often unmet. For example, the deleterious effects of anesthesia on the respiratory system of obese patients are exacerbated when compared with nonobese patients. The known decrease in end-expiratory lung volume following induction of general anesthesia is striking in obese patients, mainly because of formation of lung atelectasis, which may impair gas exchange [43]. Previous studies addressed the effects of intraoperative mechanical ventilation strategies aimed at reverting the formation of atelectasis during general anesthesia in obese patients. Respiratory strategies that increase the pressure of the airways during induction of anesthesia, such as the application of noninvasive ventilation [44], use of PEEP with [45] or without RMs [46], or a combination of all of these [47], seem to be useful for improving the respiratory function of obese patients in the pre- and intraoperative periods [48]. Nevertheless, their effects seem to be short-lived in the postoperative period [49, 50]. To our knowledge, the impact of such strategies on clinically relevant outcome endpoints, such as adverse pulmonary events, has not previously been addressed. Therefore, we believe PROBESE is the first large, international, multicenter randomized controlled trial addressing the effects of PEEP during protective low V_T_ on postoperative outcome.

We opted for testing the impact of two ventilation strategies at the same low V_T_ but mainly differing in the level of PEEP. The decision to use a PEEP value of 4 cmH_2_O in the low-PEEP group derives mainly from reports on the practice of intraoperative mechanical ventilation in obese patients [11, 51], but it also takes into account aspects regarding patient safety. In fact, use of PEEP of 0 cmH_2_O during anesthesia in morbidly obese patients is still common practice and is usually compensated for by means of proportionally high V_T_ values [52]. The level of PEEP in the high-PEEP group has been intensely debated by the steering committee of the trial. The decision to use a PEEP of 12 cmH_2_O was based on reports of several small clinical studies showing that such a level of PEEP is able to preserve the end-expiratory lung volume after induction of anesthesia [46, 53] and to avoid development of significant atelectasis when preceded by RMs [45]. Theoretically, PEEP values >12 cmH_2_O might be even more effective to avoid progressive derecruitment of lungs, but they could also result in more severe impairment of hemodynamics [54, 55]. In fact, a previous trial by our group with nonobese patients undergoing open abdominal surgery showed that high PEEP levels are more frequently associated with intraoperative hypotension that in turn requires more fluids and support with vasoactive drugs [7]. We also decided not to use an individual titration of PEEP for two reasons. First, even a PEEP titrated to a respiratory mechanical target, such as the elastance of the respiratory system, represents a compromise in terms of regional overdistention and collapsing-reopening of lung units and does not fully prevent atelectasis formation. Second, the setting of PEEP shall be pragmatic (i.e., practicable for anesthetists worldwide) while keeping the physiological rationale. The RM is based on a stepwise increase of V_T_ and PEEP, which allows opening of lung units without interruption of mechanical ventilation [40] and ensures standardization across different centers [7]. Furthermore, because the maneuver was designed for volume-controlled ventilation, it can be performed with practically all anesthesia ventilators. During RMs, the target airway pressure in the range of 40–50 cmH_2_O was based on previous functional studies in obese patients. Also, the inspiratory time of approximately 5 seconds was chosen to allow enough pressure versus time product to open atelectatic lung units [56]. We opted for recruiting lungs not only after intubation but also every hour thereafter in order to revert possible progressive derecruitment at PEEP of 12 cmH_2_O. For both the lower- and higher-PEEP groups, rescue protocols for the progression of intraoperative hypoxemia were defined to protect patients while allowing a standardized approach that minimizes interference with the respective interventions. Importantly, deviations from the protocol, even rescue because of hypoxemia, are explicitly allowed, provided this is in the best interest of the patient.

It is worth noting that recommendations have been made also with regard to different phases and aspects of the anesthetic procedure, including monitoring, choice of anesthetic agents, muscle paralysis and its reversal, intravascular volume loading and maintenance, postoperative analgesia target, and respiratory management at induction and emergence of anesthesia (e.g., use of continuous or noninvasive positive pressure and positioning). However, PROBESE is a pragmatic study, and influence on local practice of respective sites is being kept to a minimum, focusing on factors that are more directly related to the hypothesis being investigated.

Besides postoperative respiratory failure, several other adverse pulmonary events seem to add to the odds of mortality in the surgical population. In-hospital length of stay and mortality increase with the number of single pulmonary AEs in the postoperative period [1]. For this reason, in the PROBESE trial, we opted for a binary collapsed composite of single adverse pulmonary events as a primary endpoint, despite the fact that single events may differ in terms of severity. Therefore, the use of PPCs as a primary endpoint in the PROBESE trial not only has clinical relevance for the practicing anesthetist but also increases the study power because of summation of the incidence of single AEs. In spite of this, the study analysis will address not only the composite itself but also the incidence of each element separately, as well as a secondary composite that excludes mild respiratory failure. Furthermore, given the importance of minimally invasive surgical techniques in the obese population, we will conduct a separate analysis of the primary endpoint in patients undergoing laparoscopic surgery, as well as of patients with obesity class III of the WHO (BMI ≥40 kg/m^2^).

Not only the respiratory system but also other organ systems may be impaired in the postoperative period in obese patients. Thus, the analysis will also address the impact of intraoperative mechanical ventilation on single organs and a collapsed composite of nonpulmonary AEs, namely the PEPCs. In addition, further relevant outcome measures that might be related to PPCs and PEPCs, especially the length of hospital stay, will be addressed. This outcome variable not only is a measure of morbidity but has also direct impact on related health costs. Because we anticipate that, during surgery, both the lower- and the higher-PEEP groups will experience impacts on intraoperative oxygenation, respiratory system mechanics, and arterial blood pressure, intraoperative respiratory function and hemodynamic variables will also be evaluated.

Much attention has been paid to safety in the PROBESE trial. Accordingly, data and patient safety during the PROBESE trial is closely monitored by a DSMB whose members have been chosen for their expertise in clinical research, as well as by a serious AE/AE manager. The web-based approach for research electronic data capture (REDCap™) will be used for building the database within a secure system and allowing access to the electronic CRF as well as randomization of patients into groups within one single platform from all participating sites across the world.

In summary, PROBESE is the first multicenter, international, adequately powered randomized controlled trial that compares the effects of two different levels of intraoperative PEEP during protective low V_T_ on PPCs in obese patients. The results of the PROBESE trial will support anesthesiologists in their decision to set PEEP during general anesthesia for surgery in obese patients.

### Trial status

The PROBESE trial is currently recruiting patients.

### Additional files

Additional file 1: PROBESE Study protocol version 2.5. This PDF file includes the most recent version of the PROBESE Study protocol with changes highlighted. (PDF 870 kb)

Additional file 2: Standard Protocol Items: Recommendations for Interventional Trials (SPIRIT) 2013 checklist: recommended items to address in a clinical trial protocol and related documents. (PDF 122 kb).

Additional file 3: PROBESE case report form version 1.2.2. This file corresponds to the paper version of the case report form. (DOC 1610 kb).

Additional file 4: Standard operating procedures (SOP) for plasma Sampling. (PDF 115 kb)

Additional file 5: Standard operating procedures (SOP) for plasma Sampling. (PDF 110 kb)

## Appendix 1

**Table 4:** PROBESE investigators

Site ID	Site name	Collaborator(s)	Email address(es)
001	Department of Anesthesiology and Intensive Care Medicine, Pulmonary Engineering Group, University Hospital Dresden, Germany	Andreas Güldner Robert Huhle Christopher Uhlig Luigi Vivona Alice Bergamaschi	andreas.güldner@uniklinikum-dresden.de robert.huhle@tu-dresden.de christopher.uhlig@uniklinikum-dresden.de vivonag@tiscali.it alice_bergamaschi@libero.it
002	Department of Anesthesiology, University of Aachen, Germany	Rossaint, Rolf Stevanovic, Ana	rrossaint@ukaachen.de astevanovic@ukaachen.de
003	Department of Anesthesiology CLIPS Clinical Trials – Patient-centered Studies, University Hospital Düsseldorf, Germany	Treschan, Tanja Maximilian Schaefer Kienbaum, Peter	tanja.treschan@med.uni-duesseldorf.de maximilian.schaefer@med.uni-duesseldorf.de peter.kienbaum@med.uni-duesseldorf.de
004	Department of Anesthesiology, University Medical Center Mainz, Germany	Laufenberg-Feldmann, Rita	rita.laufenberg@unimedizin-mainz.de
005	Department of Anesthesiology, Intensive Care and Pain Medicine, University Clinic Knappschaftskrankenhaus Bochum, Germany	Bergmann, Lars Ebner, Felix Robitzky, Luisa Mölders, Patrick Unterberg, Matthias	lars.bergmann@kk-bochum.de felix.ebner@kk-bochum.de luisa.robitzky@gmail.com patrick.moelders@kk-bochum.de matthias.unterberg@kk-bochum.de
006	Department of Anesthesiology, University Hospital Heidelberg, Germany	Busch, Cornelius	cornelius.busch@med.uni-heidelberg.de
007	Department of Anesthesiology, Marienhospital Wesel, Germany	Achilles, Marc	marc.achilles@prohomine.de
008	Department of Anesthesiology and Intensive Care Medicine, Marienstift Friesoythe, Germany	Menzen, Angelika Freesemann, Harbert	dr.menzen@marienstift-friesoythe.de dr.freesemann@smhf.de
009	Department of Anesthesiology and Intensive Care Medicine, University Hospital Bonn, Germany	Putensen, Christian	putensen@uni-bonn.de
011	Serviço de Anestesiologia, Centro Hospitalar do Porto, Portugal	Machado, Humberto Cavaleiro, Carla Ferreira, Cristina Pinho, Daniela Carvalho, Marta Pinho, Sílvia Soares, Maria	director.anestesia@hgsa.min-saude.pt cscavaleiro@gmail.com crispintoferreira@gamil.com daniepinho@gmail.com marta.monteiro.carvalho@gmail.com silviaabpinho@gmail.com mariapereirasoares@gmail.com
012	Serviço de Anestesiologia, Centro Hospitalar Entre o Douro e Vouga, Santa Maria da Feira, Portugal	Castro, Diogo Sousa	diogosousacastro@hotmail.com
013	Serviço de Anestesiologia, Centro Hospitalar São João, Porto, Portugal	Abelha, Fernando	fernando.abelha@gmail.com
014	Serviço de Anestesiologia, Hospital Pedro Hispano, Matosinhos, Portugal	Rabico, Rui	rrabico@gmail.com
021	Department of Anesthesiology, Montefiore Medical Center Bronx, New York, NY, USA	Delphin, Ellise	edelphin@montefiore.org
022	Department of Anesthesiology, Mayo Clinic, Rochester, MN, USA	Sprung, Juraj Weingarten, Toby N. Kellogg, Todd A. Martin, Yvette N. McKenzie, Travis J	sprung.juraj@mayo.edu weingarten.toby@mayo.edu kellogg.todd@mayo.edu martin.yvette@mayo.edu mckenzie.travis@mayo.edu
023	Department of Anesthesiology, Mayo Clinic, Jacksonville, FL, USA	Brull, Sorin J. Renew, J. Ross	brull.sorin@mayo.edu renew.j@mayo.edu
024	Department of Anesthesiology, Mayo Clinic, Phoenix, AZ, USA	Ramakrishna, Harish	ramakrishna.harish@mayo.edu
025	Department of Anesthesiology, University of Colorado SOM, Aurora, CO, USA	Fernandez-Bustamante, Ana	ana.fernandez-bustamante@ucdenver.edu
026	Department of Anesthesiology, Tufts Medical Center, Boston, MA, USA	Balonov, Konstantin	kbalonov@tuftsmedicalcenter.org
027	Department of Anesthesiology, University of Mississippi, Jackson, MS, USA	Baig, Harris R.	hbaig@umc.edu
028	Department of Anesthesiology, University of Chicago, Chicago, IL, USA	Kacha, Aalok	akacha@dacc.uchicago.edu
031	Department of Anesthesiology, Pontificia Universidad Católica de Chile, Santiago de Chile, Chile	Pedemonte, Juan C. Altermatt, Fernando Corvetto, Marcia A. Paredes, Sebastian Carmona, Javiera Rolle, Augusto	jcpedemo@gmail.com falterma@med.puc.cl marciacorvetto@gmail.com sparedese@gmail.com javiera.carmona.b@gmail.com arollep@gmail.com
041	Department of Intensive Care, Academic Medical Center, University of Amsterdam, The Netherlands	Bos, Elke Beurskens, Charlotte	e.m.bos@amc.uva.nl c.j.beurskens@amc.uva.nl
042	Department of Anesthesiology, Leiden University Medical Center, Leiden, The Netherlands	Veering, B	b.t.h.veering@lumc.nl
043	Department of Anesthesiology, Westfriesgasthuis, Hoorn, The Netherlands	Zonneveldt, Harry	h.zonneveldt@westfriesgasthuis.nl
044	Department of Anesthesiology, VU Medical Center, Amsterdam, The Netherlands	Boer, Christa	m.w.hollmann@amc.uva.nl
045	Department of Anesthesiology, Onze Lieve Vrouwen Gasthuis (OLVG), Amsterdam, The Netherlands	Godfried, Marc Thiel, Bram	m.b.godfried@olvg.nl b.thiel@olvg.nl
051	Department of Anesthesiology and General Intensive Care, Medical University of Vienna, Austria	Kabon, Barbara Reiterer, Christian	barbara.kabon@meduniwien.ac.at christian.reiterer@meduniwien.ac.at
061	Department of Anesthesiology, Hospital Universitari Germans Trias i Pujol, Badalona, Spain	Canet, Jaume Tolós, Raquel Sendra, Mar González, Miriam Gómez, Noemí	jcanet.germanstrias@gencat.cat rakeltp@yahoo.es marsendra@gmail.com miriamgn@hotmail.com noe6gl@hotmail.com
062	Department of Anesthesiology and Critical Care, Hospital Clinico Universitario de Valencia, Spain	Ferrando, Carlos Tania Socorro Ana Izquierdo Marina Soro	cafeoranestesia@gmail.com austania@gmail.com anaizpa@gmail.com soromarina@gmail.com
063	Department of Anesthesiology, Critical Care and Pain Relief, Consorcio Hospital General Universitario de Valencia, Spain	Granell Gil, Manuel Hernández Cádiz, María José Biosca Pérez, Elena	mgranellg@hotmail.com mj.herca@hotmail.com ebipe@hotmail.com
064	Department of Anesthesiology and Surgical Critical Care, Hospital Universitario La Paz, Madrid, Spain	Suarez-de-la-Rica, Alejandro Lopez-Martinez, Mercedes Huercio, Iván Maseda, Emilio Yagüe, Julio	alejandro.suarez.delarica@gmail.com mercelopezmartinez@yahoo.es ivanhuercio@gmail.com emilio.maseda@gmail.com juyaru@gmail.com
065	Department of Anesthesiology, Hospital Universitari Mútua de Terrassa, Spain	Cebrian Moreno, Alba	a.cebrian.moreno@gmail.com
066	Department of Anesthesiology, Hospital Clinic de Barcelona, Spain	Rivas, Eva Lopez-Baamonde, Manuel	erivas@clinic.ub.es lopez10@clinic.cat
071	Depts. of Anesthesiology, ^1^King Abdullah Medical City, Makkah, Saudi Arabia & ^2^Assiut University- EGYPT & ^3^Mansoura University- EGYPT.	^1,2^ Elgendy, Hamed ^1,3^Sayedalahl,Mohamed	helgendy70@gmail.com al-ahl.m@kamc.med.sa
072	Department of Anesthesiology, King Abdulaziz National Guard Medical City, Riyadh, Saudi Arabia	Sibai, Abdul Razak	sibaiab@ngha.med.sa
081	Department of Anaesthesiology, Istanbul Medical Faculty, Istanbul University, Istanbul, Turkey	Yavru, Aysen Sivrikoz, Nukhet Karadeniz, Meltem	aysenyg@gmail.com ntsz06@gmail.com mskaradeniz@gmail.com
082	Department of Anesthesiology and Reanimation, Marmara University School of Medicine, Istanbul, Turkey	Corman Dincer, Pelin Ayanoglu, Hilmi Omer Tore Altun, Gulbin Kavas, Ayse Duygu	pelincorman@yahoo.com ayanoglu@mamara.edu.tr dr_gulbin@yahoo.com adkavas@hotmail.com
083	Department of Anesthesiology and Reanimation, Akdeniz University Hospital, Antalya, Turkey	Dinc, Bora	drboradinc@gmail.com
084	Department of Anesthesiology and Reanimation, Dokuz Eylül University of Medicine, Izmir, Turkey	Kuvaki, Bahar Ozbilgin, Sule	bkuvaki@deu.edu.tr ozbilginsule@gmail.com
085	Department of Anesthesiology and Reanimation, Fatih Sultan Mehmet Educational and Research Hospital, Istanbul, Turkey	Erdogan, Dilek Koksal, Ceren Abitagaglu, Suheyla	dilekerdoganari@gmail.com ceren_hazer@yahoo.com suheylaatay81@gmail.com
091	Department of Anaesthesiological, Surgical and Emergency Sciences, Second University of Naples, Italy	Aurilio, Caterina Sansone, Pasquale Pace, Caterina Maria Donatiello, Valerio Mattera, Silvana Palange, Nazareno Di Colandrea, Salvatore	caterina.aurilio@unina2.it pasquale.sansone@unina2.it caterina.pace@libero.it valerio.donatiello@gmail.com silvanamattera@gmail.com nazareno.palange@gmail.com di.colandrea@libero.it
092	Department of Morphology, Surgery and Experimental Medicine, Ospedale Sant’ Anna, Ferrara, Italy	Spadaro, Savino Volta, Carlo Alberto Ragazzi, Riccardo Ciardo, Stefano Gobbi, Luca	savinospadaro@gmail.com vlc@unife.it rgc@unife.it stefano.ciardo@student.unife.it lucagobbi1988@gmail.com
093	Department of Environment, Health and Safety, University of Insubria, Varese, Italy	Severgnini, Paolo Bacuzzi, Alessandro Brugnoni, Elisa	paolo.severgnini@uninsubria.it alessandro.bacuzzi@gmail.com elisa.brugnoni@gmail.com
094	Dept. of Surgical Sciences and Integrated Diagnostics, IRCCS AOU San Martino, IST, University of Genoa, Italy	Gratarola, Angelo Micalizzi, Camilla Simonassi, Francesca Malerbi, Patrizia	a.gratarola@gmail.com camilla.micalizzi@gmail.com fra.simonassi@libero.it patrizia.malerbi@hsanmartino.it
095	Department of Anesthesiology and Reanimation, Ospedale U. Parini, AUSL della Valle d’ Aosta, Italy	Carboni, Adrea	acarboni@ausl.vda.it
101	Department of Anesthesiology, Pharmacology & Intensive Care, University Hospital Geneva, Switzerland	Licker, Marc-Joseph	marc-joseph.licker@hcuge.ch
102	Institute for Anesthesia and Intensive Care Medicine, Kantonsspital Frauenfeld, Switzerland	Dullenkopf, Alexander	alexander.dullenkopf@stgag.ch
103	Department of Anesthesia, Surgical Intensive Care, Prehospital Emergency Medicine and Pain Therapy, University Hospital Basel, Switzerland	Goettel, Nicolai	nicolai.goettel@usb.ch
111	University Department of Anesthesiology, Resuscitation and Intensive Care, University Hospital Sveti Duh, Zagreb, Croatia	Nesek Adam, Visnja Karaman Ilić, Maja	visnja.nesek@hotmail.com majakilic@gmail.com
112	Department of Anesthesiology, Resuscitation and Intensive Care, Clinical Hospital Dubrava, Zagreb, Croatia	Klaric, Vlasta Vitkovic, Bibiana Milic, Morena Miro, Zupcic	vllastica@gmail.com bibianavit@hotmail.com morena.milic@gmail.com miro_zupcic@yahoo.com
121	Department of Anesthesiology, Ghent University Hospital, Belgium	De Baerdemaeker, Luc De Hert, Stefan Heyse, Bjorn Van Limmen, Jurgen Van Nieuwenhove, Yves	luc.debaerdemaeker@ugent.be stefan.dehert@ugent.be bjorn.heyse@ugent.be jurgen.vanlimmen@ugent.be yves.vannieuwenhove@ugent.be
123	Department of Anesthesiology, UH Antwerpen, Belgium	Mertens, Els	els.mertens@uza.be
124	Department of Anesthesiology, UZ Leuven, Belgium	Neyrinck, Arne	arne.neyrinck@uzleuven.be
125	Department of Anaesthesiology, Intensive care and Emergency, AZ Sint Jan Brugge, Bruges, Belgium	Mulier, Jan	jan.mulier@azsintjan.be
126	Department of Anesthesiology, Cliniques Universitaires St Luc, Belgium	Kahn, David	david.kahn@uclouvain.be
131	Department of Anesthesiology, Ponderas Hospital, Bucharest, Romania	Godoroja, Daniela	danielagodoroja@yahoo.com
141	Department of Intensive Care, St James’s University Hospital, Dublin, Ireland	Martin-Loeches, Martin	drmartinloeches@gmail.com
151	Department of Anaesthesiology and Intensive Care, Zaporozhye State Medical University, Zaporozhye, Ukraine	Vorotyntsev, Sergiy	vorotyntsev_s@ukr.net
152	Department of Anaesthesiology and Intensive Care, Lutsk Clinical Hospital, Lutsk, Ukraine	Fronchko, Valentyna	fron@ua.fm
161	Division of Anesthesiology, Critical Care and Pain Medicine, Tel Aviv Medical Center, Sackler School of Medicine, Tel Aviv, Israel	Matot, Idit Goren, Or Zac, Lilach	iditm@tlvmc.gov.il goren.orr@gmail.com isanaesfile@gmail.com
171	Department of Anaesthesiology and Intensive Therapy, Barlicki University Hospital, Medical University of Lodz, Poland	Gaszynski, Thomasz	tomasz.gaszynski@umed.lodz.pl
181	Department of Anesthesiology and Critical Care, Saint Michael’s Hospital, University of Toronto, Canada	Laffey, Jon	laffeyj@smh.ca
191	Operating Services, Critical Care and Anaesthesia (OSCCA), Sheffield Teaching Hospitals, United Kingdom	Mills, Gary	g.h.mills@sheffield.ac.uk
192	Department of Anaesthesia and Critical Care Medicine, University Hospital of North Midlands, Stoke-on-Trent, United Kingdom	Nalwaya, Pramod	pramod.nalwaya@uhns.nhs.uk
193	Research Divisional Lead – Surgery and Oncology, Research and Development Department, Ashford and St Peter Hospitals NHS Trust, Chertsey, United Kingdom	Mac Gregor, Mark	mark.macgregor@asph.nhs.uk
194	Department of Anaesthesia, Royal Cornwall Hospital, Truro, United Kingdom	Paddle, Jonathan	jonathan.paddle@nhs.net
195	Department of Anaesthesia, Hull and East Yorkshire NHS Trust, Hull, United Kingdom	Balaji, Packianathaswamy	indbalaji@gmail.com
196	Department of Critical Care Medicine, Imperial College Healthcare NHS Trust, London, United Kingdom	Rubulotta, Francesca Adebesin, Afeez	frubulotta@hotmail.com afeez.adebesin@imperial.nhs.uk
197	Department of Anaesthesia, Western Sussex Hospitals NHS Foundation Trust, Chichester, United Kingdom	Margarson, Mike	michael.margarson@wsht.nhs.uk
198	Department of Anaesthesia, York Teaching Hospitals, York, United Kingdom	Davies, Simon	drsimondavies@googlemail.com
199	Department of Anaesthesia, Homerton University Hospitals NHS Foundation Trust, Homerton, United Kingdom	Rangarajan, Desikan	desikan.rangarajan@homerton.nhs.uk
190	Department of Anesthesia, Southmead Hospital, Bristol, United Kingdom	Newell, Christopher	christopher.newell@doctors.org.uk
201	Department of Anaesthesia and Critical Care Medicine, University Clinic of Surgery St. Naum Ohridski, Skopje, Macedonia	Shosholcheva, Mirjana	sosolceva@hotmail.com
211	Department of Anaesthesia, Polyclinique Montier La Celle, St. Andre les Vergers, France	Papaspyros, Fotios	papaspyrosf@outlook.com
221	Department of Anaesthesia, Alexandra General Hospital, Athens, Greece	Skandalou, Vasiliki	vps_71@hotmail.com
	Department of Anaesthesiology and Intensive Medicine, University Hospital of Ostrava, Ostrava-Poruba, Czech Republic	Dzurňáková, Paula	paula.dzurnakova@fno.cz

## Appendix 2

**Table 5:** Ethics committees that approved the study

001	Dresden	Department of Anesthesiology and Intensive Care Medicine, Pulmonary Engineering Group, University Hospital Dresden, Germany	Ethics Committee of the Medical Faculty of the Technical University Dresden ID: EK 430112013
002	Aachen	Department of Anesthesiology, University of Aachen, Germany	Ethics Committee of the Medical Faculty of RWTH Aachen ID: EK 181/14
003	Düsseldorf	Department of Anesthesiology CLIPS Clinical Trials – Patient-centered Studies, University Hospital Düsseldorf, Germany	Ethics Committee of the Medical Faculty of HHU Düsseldorf ID: 2014072709
004	Mainz	Department of Anaesthesiology, University Medical Center Mainz, Germany	Ethics Committee of the Medical Faculty of UMC Mainz ID: 837.307.14 (9546)
005	Bochum	Clinic of Anesthesiology, Intensive Care and Pain Medicine, University Clinic Knappschaftskrankenhaus Bochum, Germany	Ethics Committee of the Medical Faculty, Ruhr University Bochum ID: 5110-14
006	Heidelberg	Department of Anesthesiology, University Hospital Heidelberg, Germany	Ethics Committee of the Medical Faculty, University of Heidelberg ID: S-442/2014
007	Wesel	Department of Anesthesiology, Marienhospital Wesel, Germany	Ethics Committee of the Medical Council Nordrhein, Düsseldorf ID: 2014497
008	Friesoythe	Department of Anesthesiology and Intensive Care Medicine, Marienstift Friesoythe, Germany	Ethics Committee of the Medical Council Niedersachsen, Hannover ID: Grae/045/2015
009	Bonn	Department of Anesthesiology and Intensive Care Medicine, University Hospital Bonn, Germany	Ethics Committee of the Medical Faculty of the University of Bonn ID: 288/15
011	Porto	Serviço de Anestesiologia, Centro Hospitalar do Porto, Portugal	Ethics and Health Committee of Centro Hospitalar do Porto ID: 2014.144 (100-DEFI/130-CES)
012	Santa Maria da Feira	Serviço de Anestesiologia, Centro Hospitalar Entre o Douro e Vouga, Santa Maria da Feira, Portugal	Ethics and Health Committee of Centro Hospitalar Entre o Douro e Vouga, Santa Maria da Feira ID: 07/ANES/2014
013	Porto	Serviço de Anestesiologia, Centro Hospitalar São João, Porto, Portugal	Ethics and Health Committee of Centro Hospitalar São João, Porto ID: CES 167-14
014	Matosinhos	Serviço de Anestesiologia, Hospital Pedro Hispano, Matosinhos, Portugal	Ethics and Health Committee of Hospital Pedro Hispano, Matosinhos ID: 076/CE/JAS
021	New York	Department of Anesthesiology, Montefiore Medical Center Bronx, New York, NY, USA	Institutional Review Board at Montefiore Medical Center Bronx, New York ID: 2014-3761
022	Rochester, Minnesota	Department of Anesthesiology, Mayo Clinic, Rochester, MN, USA	Institutional Review Board Mayo Clinic ID: 14-005465
023	Jacksonville, Florida	Department of Anesthesiology, Mayo Clinic, Jacksonville, FL, USA	Institutional Review Board Mayo Clinic ID: 14-005465
024	Phoenix, Arizona	Department of Anesthesiology, Mayo Clinic, Phoenix, AZ, USA	Institutional Review Board Mayo Clinic ID: 14-005465
025	Aurora, Colorado	Department of Anesthesiology, University of Colorado SOM, Aurora, CO, USA	ŒColorado Multiple Institutional Review Board (COMIRB) ID: 14-1495
026	Boston	Department of Anesthesiology, Tufts Medical Center, Boston, MA, USA	Institutional Review Board at Tufts Medical Center, Boston ID: 11808
027	Jackson	Department of Anesthesiology, University of Mississippi, Jackson, MS, USA	Institutional Review Board at University of Mississippi, Jackson ID: 2015-0080
028	Chicago	Department of Anesthesiology, University of Chicago, Chicago, IL, USA	Institutional Review Board at University of Chicago, Chicago ID: 14-1044
031	Santiago de Chile	Department of Anesthesiology, Pontificia Universidad Católica de Chile, Santiago de Chile, Chile	Comite de Etica en Investigacion Escuela de Medicina Pontificia Universidad Catolica de Chile ID: 14-462
041	Amsterdam	Department of Intensive Care, Academic Medical Center, University of Amsterdam, The Netherlands	Medisch Ethische Toetsingscommissie (METC) of the Academic Medical Center (AMC), Amsterdam ID: 2014_261
042	Leiden	Department of Anesthesiology, Leiden University Medical Center, Leiden, The Netherlands	Ethics Committee of the Leiden University Medical Center (LUMC), Leiden ID: P15.038
043	Hoorn	Department of Anesthesiology, Westfriesgasthuis, Hoorn, The Netherlands	Ethics Committee of Westfriesgasthuis, Hoorn ID: WB 486
044	Amsterdam	Department of Anesthesiology, VU Medical Center, Amsterdam, The Netherlands	Ethics Committee of VU Medical Center (VUMC), Amsterdam ID: 2014.561
045	Amsterdam	Department of Anesthesiology, Onze Lieve Vrouwen Gasthuis (OLVG), Amsterdam, The Netherlands	Ethics Committee of the Onze Lieve Vrouwen Gasthuis (OLVG), Amsterdam ID: WO 14.133
051	Vienna	Department of Anesthesiology and General Intensive Care, Medical University of Vienna, Austria	Ethics committee of the Medical University of Vienna ID: 1702/2014
061	Badalona	Department of Anesthesiology, Hospital Universitari Germans Trias i Pujol, Badalona, Spain	Comité d’Ètica de la Investigació. Hospital Universitari Germans Trias i Pujol, Badalona ID: AC-14-095
062	Valencia	Department of Anesthesiology and Critical Care, Hospital Clinico Universitario de Valencia, Spain	Ethics Committee of the Hospital Clinico Universitario de Valencia ID: F-CE-GEva-15, approved 27/11/2014
063	Valencia	Department of Anesthesiology, Critical Care and Pain Relief, Consorcio Hospital General Universitario de Valencia, Spain	Ethics Committee of the Consorcio Hospital General Universitario of Valencia ID: 223/2004
064	Madrid	Department of Anesthesiology and Surgical Critical Care, Hospital Universitario La Paz, Madrid, Spain	Ethics Committee of the Hospital Universitario La Paz, Madrid ID: 4465 12/2015
065	Terrassa	Department of Anesthesiology, Hospital Universitari Mútua de Terrassa, Spain	Ethics Committee of the Hospital Universitari Mútua de Terrassa, Terassa ID: 04/15, approved 29/04/2015
066	Barcelona	Department of Anesthesiology, Hospital Clinic de Barcelona, Spain	Research Ethics Committee of the Hospital Clínic of Barcelona ID: HCB/2015/0963
071	Makkah	Department of Anesthesiology, King Abdullah Medical City, Makkah, Saudi Arabia	King Abdullah Medical City (KAMC) Local IRB registered at the National BioMedical Ethics Committee, King Abdulaziz City for Science and Technology ID: 14-144
072	Riyadh	Department of Anesthesiology, King Abdulaziz National Guard Medical City, Riyadh, Saudi Arabia	Ethics Committee of the King Abdulaziz National Guard Medical City, Riyadh ID: IRBC/215/16
081	Istanbul	Department of Anaesthesiology, Istanbul Medical Faculty, Istanbul University, Istanbul, Turkey	Clinical Research Ethics Committee of the Istanbul Faculty of Medicine, Istanbul University, Istanbul IB: 1417, approved 18/9/2014
082	Istanbul	Department of Anesthesiology and Reanimation, Marmara University School of Medicine, Istanbul, Turkey	Clinical Research Ethics Committee of the Istanbul Faculty of Medicine, Istanbul ID: 09.2015075
083	Antalya	Department of Anesthesiology and Reanimation, Akdeniz University Hospital, Antalya, Turkey	Ethics Committee of Akdeniz University Hospital, Antalya ID: 170, approved 17/04/2015
084	Izmir	Department of Anesthesiology and Reanimation, Dokuz Eylül University of Medicine, Izmir, Turkey	Ethics Committee of Dokuz Eylul University Medical Faculty ID: 66, approved 23/01/2015
085	Istanbul	Department of Anesthesiology and Reanimation, Fatih Sultan Mehmet Educational and Research Hospital, Istanbul, Turkey	Ethics committee of Fatih Sultan Mehmet Educational and Research Hospital, Istanbul ID: 17073117-050-99
091	Napoli	Department of Anaesthesiological, Surgical and Emergency Sciences, Second University of Naples, Italy	Ethics committee of Second University of Naples ID: 29.GEN.2015/128
092	Ferrara	Department of Morphology, Surgery and Experimental Medicine, Ospedale Sant’ Anna, Ferrara, Italy	Ethics Committee of Ferrara ID: 15097, approved 26/03/2015
093	Varese	Department of Environment, Health and Safety, University of Insubria, Varese, Italy	Ethics Committee – ASST Sette Laghi Ospedale di Circolo e Fondazione Macchi, Varese, Italy ID: 0034259
094	Genoa	Dept. of Surgical Sciences and Integrated Diagnostics, IRCCS AOU San Martino, IST, University of Genoa, Italy	Regional Ethics Committee Liguria, Italy ID: 121.REG.2015
095	Aosta	Department of Anesthesiology and Reanimation, Ospedale U. Parini, AUSL della Valle d’ Aosta, Italy	Ethics Committee of AUSL della Valle d’ Aosta, Aosta ID: 737, approved 19/06/2015
101	Geneva	Department of Anesthesiology, Pharmacology & Intensive Care, University Hospital Geneva, Switzerland	Ethics Committee of University Hospital Geneva ID: 14-238
102	Frauenfeld	Department of Anesthesiology and Intensive Care, Kantonsspital Frauenfeld, Switzerland	Ethics Committee of the Kanton Thurgau, Münsterlingen ID: A2015/33
103	Basel	Department of Anesthesia, Surgical Intensive Care, Prehospital Emergency Medicine and Pain Therapy, University Hospital Basel, Switzerland	Ethikkommission Nordwest- und Zentralschweiz ID: PB_2016-00313
111	Zagreb	University Department of Anesthesiology, Resuscitation and Intensive Care, University Hospital Sveti Duh, Zagreb, Croatia	Ethics Committee of the Clinical Hospital Sveti Duh ID: 01-12/4
112	Zagreb	Department of Anesthesiology, Resuscitation and Intensive Care, Clinical Hospital Dubrava, Zagreb, Croatia	Ethics Committee of the University Zagreb School of Medicine and Ethics Committee of Clinical Hospital Dubrava Zagreb ID: none provided, approved 23/06/2015
121	Ghent	Department of Anesthesiology, Ghent University Hospital, Belgium	Ethics Committee of Ghent University Hospital ID: B670201523757
123	Antwerpen	Department of Anesthesiology, UH Antwerpen, Belgium	Ethics Committee of Ghent University Hospital ID: B670201523757
124	Leuven	Department of Anesthesiology, UZ Leuven, Belgium	Ethics Committee of Ghent University Hospital ID: B670201523757
125	Bruges	Department of Anaesthesiology, Intensive care and Emergency, AZ Sint Jan Brugge, Bruges, Belgium	Ethics Committee of Ghent University Hospital ID: B670201523757
126	Brussels	Department of Anesthesiology, Cliniques Universitaires St Luc, Belgium	Ethics Committee of Ghent University Hospital ID: B670201523757
131	Bucharest	Department of Anesthesiology, Ponderas Hospital, Bucharest, Romania	Ethic Council of the Ponderas Hospital, Bucharest ID: 73, approved /23/06/2015
141	Dublin	Department of Critical Care and burns unit, SR James’s University Hospital, Dublin, Ireland	Ethics Committee of St. James’s University Hospital, Dublin ID: 2015/05/02
151	Zaporozhye	Department of Anaesthesiology and Intensive Care, Zaporozhye State Medical University, Zaporozhye, Ukraine	Ethics Committee of the Zaporozhye State Medical University, Zaporozhye ID: 5/2015
152	Lutsk	Department of Anaesthesiology and Intensive Care, Lutsk Clinical Hospital, Lutsk, Ukraine	Ethics Committee of the Lutsk Clinical Hospital, Lutsk ID: 354/1.3.7.15
161	Tel Aviv	Division of Anesthesiology, Critical Care and Pain Medicine, Tel Aviv Medical Center, Sackler School of Medicine, Tel Aviv, Israel	Tel Aviv Medical Center Helsinki Committee ID: 0509-14-TLV
171	Lodz	Department of Anaesthesiology and Intensive Therapy, Barlicki University Hospital, Medical University of Lodz, Poland	Ethics Committee of Medical University of Lodz, Poland ID: RNN/134/15/KE
181	Toronto	Department of Anesthesiology and Critical Care, Saint Michael’s Hospital, University of Toronto, Canada	St Michael’s Hospital Research Ethics Board ID: 15-134
191	Sheffield	Operating Services, Critical Care and Anaesthesia (OSCCA), Sheffield Teaching Hospitals, United Kingdom	NISCHR Research Ethics Service, Wales REC4,Wrexham ID: 15/WA/0106
192	Stoke-on-Trent	Department of Anaesthesia and Critical Care Medicine, University Hospital of North Midlands, Stoke-on-Trent, United Kingdom	NISCHR Research Ethics Service, Wales REC4,Wrexham ID: 15/WA/0106
193	Chertsey	Research Divisional Lead – Surgery and Oncology, Research and Development Department, Ashford and St Peter Hospitals NHS Trust, Chertsey, United Kingdom	NISCHR Research Ethics Service, Wales REC4,Wrexham ID: 15/WA/0106
194	Truro	Department of Anaesthesia, Royal Cornwall Hospital, Truro, United Kingdom	NISCHR Research Ethics Service, Wales REC4,Wrexham ID: 15/WA/0106
195	Hull	Department of Anaesthesia, Hull and East Yorkshire NHS Trust, Hull, United Kingdom	NISCHR Research Ethics Service, Wales REC4,Wrexham ID: 15/WA/0106
196	London	Department of Critical Care Medicine, Imperial College Healthcare NHS Trust, London, United Kingdom	NISCHR Research Ethics Service, Wales REC4,Wrexham ID: 15/WA/0106
197	Chichester	Department of Anaesthesia, Western Sussex Hospitals NHS Foundation Trust, Chichester, United Kingdom	NISCHR Research Ethics Service, Wales REC4,Wrexham ID: 15/WA/0106
198	York	Department of Anaesthesia, York Teaching Hospitals, York, United Kingdom	NISCHR Research Ethics Service, Wales REC4,Wrexham ID: 15/WA/0106
199	Homerton	Department of Anaesthesia, Homerton University Hospitals NHS Foundation Trust, Homerton, United Kingdom	NISCHR Research Ethics Service, Wales REC4,Wrexham ID: 15/WA/0106
190	Bristol	Department of Anaesthesia, Southmead Hospital, Bristol, United Kingdom	NISCHR Research Ethics Service, Wales REC4,Wrexham ID: 15/WA/0106
201	Skopje	Department of Anaesthesia and Critical Care Medicine, University Clinic of Surgery St. Naum Ohridski, Skopje, Macedonia	Ethic Committee of the University Hospital Skopje ID: 07/3183/1
211	St. Andre les Vergers	Department of Anaesthesia, Polyclinique Montier La Celle, St. Andre les Vergers, France	Ethic Committee of the Polyclinique Montier La Celle, St. Andre les Vergers ID: non provided; approved 16/03/2016
221	Athens	Department of Anaesthesia, Alexandra General Hospital, Athens, Greece	Ethic Committee of the Alexandra General Hospital, Athens ID: 18-8/21-06-2016
	Ostrava-Poruba	Department of Anaesthesiology and Intensive Medicine, University Hospital of Ostrava, Ostrava-Poruba, Czech Republic	Ethic Committee of the University Hospital of Ostrava, Ostrava-Poruba ID: 64/2015

## Abbreviations

AE: Adverse event
ARDS: Acute respiratory distress syndrome
ARISCAT: Assess Respiratory Risk in Surgical Patients in Catalonia
BMI: Body mass index
CONSORT: Consolidated Standards of Reporting Trials
COPD: Chronic obstructive pulmonary disease
CRF: Case report form
DIC: Disseminated intravascular coagulation
DSMB: Data and Safety Monitoring Board
FiO_2_: Fraction of inspired oxygen
GIF: Gastrointestinal failure
I:E: Inspiratory-to-expiratory ratio
ICU: Intensive care unit
INR: International normalized ratio
ITT: Intention-to-treat
OSA: Obstructive sleep apnea
PaO_2_: Partial pressure of arterial oxygen
PBW: Predicted body weight
PEEP: Positive end-expiratory pressure
PEPC: Postoperative extrapulmonary complication
POD: Postoperative day
PPC: Postoperative pulmonary complication
Ppeak: Peak airway pressure
Pplat: Plateau airway pressure
PROBESE trial: PRotective Ventilation with Higher versus Lower PEEP during General Anesthesia for Surgery in OBESE Patients
REDCap™: Research Electronic Data Capture (web-based system used during PROBESE as electronic case report form)
RIFLE classification system: Risk, injury, failure, loss, end-stage kidney disease
RM: Recruitment maneuver
RR: Respiratory rate
SPIRIT: Standard Protocol Items: Recommendations for Interventional Trials
SpO_2_: Peripheral oxyhemoglobin saturation measured by pulse oximetry
SSL: Secure Sockets Layer
VAS: Visual analogue scale
V_T_: Tidal volume
WHO: World Health Organization
