# Erratum to Protective intraoperative ventilation with higher versus lower levels of positive end-expiratory pressure in obese patients (PROBESE): study protocol for a randomized controlled trial

**DOI:** 10.1186/s13063-017-1987-3

**Published:** 2017-06-01

**Authors:** T. Bluth, R. Teichmann, T. Kiss, I. Bobek, J. Canet, G. Cinnella, L. De Baerdemaeker, C. Gregoretti, G. Hedenstierna, S. N. Hemmes, M. Hiesmayr, M. W. Hollmann, S. Jaber, J. G. Laffey, M. J. Licker, K. Markstaller, I. Matot, G. Müller, G. H. Mills, J. P. Mulier, C. Putensen, R. Rossaint, J. Schmitt, M. Senturk, A. Serpa Neto, P. Severgnini, J. Sprung, M. F. Vidal Melo, H. Wrigge, M. J. Schultz, P. Pelosi, Marcelo Gama de Abreu, for the PROBESE investigators, and the PROtective VEntilation Network (PROVEnet) and on behalf of the Clinical Trial Network of the European Society of Anaesthesiology (ESA)

**Affiliations:** 1 Department of Anesthesiology and Intensive Care Medicine, Pulmonary Engineering Group, University Hospital Carl Gustav Carus, Dresden, Germany; 2 Aneszteziológiai és Intenzív Terápiás Klinika, Semmelweis Egyetem, Budapest, Hungary; 3 Department of Anesthesiology, Hospital Universitari Germans Trias I Pujol, Badalona, Spain; 4 Department of Anesthesiology and Intensive Care Medicine, University of Foggia, Foggia, Italy; 5 Department of Anesthesiology, Ghent University Hospital, Ghent, Belgium; 6 Department of DIBIMED, Policlinico P. Giaccone, Palermo, Italy; 7 Department of Medical Sciences, Section of Clinical Physiology, University Hospital, Uppsala, Sweden; 8 Department of Anesthesiology & Laboratory of Experimental Intensive Care and Anesthesiology (L·E·I·C·A), Academic Medical Center, University of Amsterdam, Amsterdam, The Netherlands; 9 Division Cardiac-, Thoracic-, Vascular Anesthesia and Intensive Care, Medical University of Vienna, Vienna, Austria; 10 Department of Critical Care Medicine and Anesthesiology (SAR B), Saint Eloi University Hospital, Montpellier, France; 11 Department of Anesthesia and Critical Care Medicine, Saint Michael’s Hospital, and Departments of Anesthesia, Physiology and Interdepartmental division of Critical Care Medicine, University of Toronto, Toronto, Canada; 12 Department of Anesthesiology, Pharmacology & Intensive Care, University Hospital Geneva, Geneva, Switzerland; 13 Department of Anesthesiology and Intensive Care Medicine, Medical University, Vienna, Austria; 14 Department of Anesthesiology and Critical Care, Tel Aviv Medical Center, Sackler School of Medicine, Tel Aviv University, Tel Aviv, Israel; 15 Center for Evidence-based Healthcare, University Hospital and Medical Faculty Carl Gustav Carus, Dresden, TU Germany; 16 Operating Services, Critical Care and Anaesthesia (OSCCA), Sheffield Teaching Hospitals and University of Sheffield, Sheffield, UK; 17 Department of Anesthesiology, AZ Sint Jan Brugge-Oostende AV, Brugge, Belgium; 18 Department of Anesthesiology and Intensive Care Medicine, University of Bonn, Bonn, Germany; 19 Department of Anesthesiology, University of Aachen, Aachen, Germany; 20 Department of Anesthesiology and Intensive Care Medicine, Istanbul Medical Faculty, University of Istanbul, Istanbul, Turkey; 21 Department of Critical Care Medicine, Hospital Israelita Albert Einstein, and Program of Post-Graduation, Research and Innovation, Faculdade de Medicina do ABC, São Paulo, Brazil; 22 Department of Biotechnology and Sciences of Life, University of Insubria, ASST-settelaghi Ospedale di Cricolo e Fondazione Macchi, Varese, Italy; 23 Department of Anesthesiology, Mayo Clinic, Rochester, MN USA; 24 Department of Anesthesia, Critical Care and Pain Medicine, Massachusetts General Hospital -Harvard Medical School, Boston, MA USA; 25 Department of Anesthesiology and Intensive Care Medicine, University of Leipzig, Leipzig, Germany; 26 Department of Intensive Care & Laboratory of Experimental Intensive Care and Anesthesiology (L·E·I·C·A), Academic Medical Center, University of Amsterdam, Amsterdam, The Netherlands; 27 Department of Surgical Sciences and Integrated Diagnostics, IRCCS AOU San Martino, IST, University of Genoa, Genoa, Italy

## Erratum

In the original publication [1] one author name was not correct.

*Wrong:* Miro, Zupcic

*Correct:* Zupcic, Miro
