# Supplementary material for: Protective intraoperative ventilation with higher versus lower levels of positive end-expiratory pressure in obese patients (PROBESE): study protocol for a randomized controlled trial
Source: Trials. 2017 Apr 28;18:202. doi: 10.1186/s13063-017-1929-0 (PMC5410049; doi:10.1186/s13063-017-1929-0)
Supplement: Supplementary file 1 — PROBESE Study protocol version 2.5. This PDF file includes the most recent version of the PROBESE Study protocol with changes highlighted. (PDF 870 kb) [file 13063_2017_1929_MOESM1_ESM.pdf]

Changes in Version 2.5 (Feb 2016) compared to Version 2.4.2 (Oct 2014) of the research protocol

- changes in the text are marked in red -

Page 12: Since the overall sample size needed to be increased, we adapted both the number of centers and the number of patients per center to be randomized. We now expect to have 90 centers (instead of 60), each randomizing at least 24 (instead of 12 to 13) eligible patients.

Page 13: Sample size was re-calculated as newly described. The original description of the methods was kept in place and slightly modified. The following text was added: *[Accordingly, the previous Sample size was re-estimated based on recommendations of the DSMB after data on the first 618 patients revealed that the incidence of the collapsed composite outcome was considerably lower than expected. In the revised calculations, the control group incidence was assumed to be 0.20 (instead of 0.40), and sample size was calculated to have 80% power at the overall 0.05 significance level to detect a relative risk (as originally planned) of 0.75. This design requires a maximum of 1912 patients, adjusting for the interim monitoring for efficacy and futility as described in section 8.2. Assuming a dropout rate of 5%, a total of 2013 patients need to be included into the study. East 6.0 interim monitoring software (Cytel, Cambridge, MA, USA) was used.]*

Page 23: Interim analyses were planned. The following text, including one table and one figure was added: *[Interim analyses for efficacy and futility will be conducted at 50% (N=956), 75% (N=1434) and 100% (N=1912) of the planned enrollment, as needed, using a non-binding group sequential design with gamma spending functions (gamma = -4 for each of alpha and beta). The table below shows the alpha and beta spent over the trial, z-statistic boundaries for efficacy and futility, and boundary crossing probabilities under the alternative hypothesis (H1). The corresponding P-value boundaries for efficacy (futility in parentheses) at the 1st, 2nd and final looks, respectively, are P≤ 0.006 (P>0.82), P≤ 0.015 (P>0.35) and P ≤ 0.044 (P>0.044). The Figure below displays the z-statistic boundaries for efficacy/harm and futility as a function of accrued sample size.]*

Page 25: Prof. A. Hoeft, University Hospital of Bonn, Germany joined the Data Safety and Monitoring Board (DSMB). Now, the DSMB is composed of 5 individuals.

Changes in Version 2.4.2 (Oct 2014) compared to Version 2.4.1 (Jun 2013) of the research protocol

Page 12: *Section 6.5* [Interim Analyses: Planned interim analyses will be performed after 50% of the patients required (n=374 patients) have been randomized and treated in the study. Early termination of the study may be considered if very strong differences between the two treatment groups become apparent. Very strong differences are defined as risk ratio smaller than 0.5 (e.g. event rate in higher PEEP group < 20%, event rate in lower PEEP group 40%) or greater than 2.0 (e.g. event rate in higher PEEP group > 80%, event rate in lower PEEP group 40%). The decision to terminate the study early will be made by a data and safety monitoring board (DSMB). Except in the case of early study termination, the results of the interim analysis will not be disclosed to the participating study centers.] *is deleted. There will be no interim analyses during the study.*

Page 22: *The statistical analysis section (8.2) is actualized and extended. The primary endpoint and its analysis plan were clearly defined.*

Page 23: *Details on how to report Adverse Events (AE) are added by using the new Appendix v. (page 34-35)*

Page 23: [Prof. H. van Aken, University of Münster, Germany] *deleted. Since he is not a member, at present, the DSMB is composed of four individuals.*

Page 33: *The study sheet (Appendix iv) was overall actualized. Mainly, patient visits on postoperative days 2 and 4 are added. These additional visits were already described in the text (page 19) in the former versions of the protocol (2.4 and 2.4.1).*

Changes in Version 2.4.1 (Jun 2014) compared to Version 2.4 (Nov 2013) of the research protocol of

Protective Ventilation with Higher versus Lower PEEP during General Anesthesia for Surgery in OBESE Patients – The PROBESE Randomized Controlled Trial

Page 1: G. Mills, Sheffield Teaching Hospitals, United Kingdom, Operating Services, Critical Care and Anaesthesia (OSCCA) *added to the steering committee*

Page 6: [While anesthesiologists tend to use PEEP higher than in non-obese patients] *deleted*

Page 11: *Inclusion criteria* [Patient scheduled for open or laparoscopic surgery under general anesthesia] *changed to* [Patient scheduled for surgery under general anesthesia]

Page 32: *Definition of Hepatic failure:* [Serum bilirubin level > 2 mg/dL with elevation of the transaminase and lactic dehydrogenase levels above twice normal values] *changed to* [Hepatic failure during short term follow up (5 postoperative days) is considered as follows: Ratio of total bilirubin on postoperative day 5 to postoperative day 1 > 1,7 and ratio of international normalized ratio (INR) on postoperative day 5 to postoperative day 1 >1,0; during long term follow up (until postoperative day 90) at new presence of hepatic encephalopathy and coagulopathy (INR > 1,5) within 8 weeks after initial signs of liver injury (e.g. jaundice) without evidence for chronic liver disease]

PRotective Ventilation with Higher versus Lower PEEP during General Anesthesia for Surgery in OBESE Patients – The PROBESE Randomized Controlled Trial

RESEARCH PROTOCOL

M. Gama de Abreu[1], T. Bluth[1], H. Wrigge[2], A. Serpa Neto[3], S.N.T. Hemmes[4], P. Severgnini[5], G. Cinnella[6], S. Jaber[7], J. Canet[8], M. Hiesmayr[9], K. Markstaller[10], J.P. Mulier[11], L. de Baerdemaeker[12], J. Sprung[13], M.F. Vidal Melo[14], J. Laffey[15], G. Hedenstierna[16], I. Matot[17], C. Putensen[18], M.J. Licker[19], R. Rossaint[20], M. Senturk[21], C. Gregoretti[22], M.W. Hollmann[4], I. Bobek[23], G. Mills[24], J. Schmitt[25], M.J. Schultz[4], P. Pelosi[26]
for the PROBESE Investigators

University Hospital Dresden, Germany
[1]Department of Anesthesiology and Intensive Care Medicine
University of Leipzig, Germany
[2]Department of Anesthesiology and Intensive Care Medicine
ABC Medical School, São Paulo, Brazil
[3]Dept. of Intensive Care Medicine
Academic Medical Center, University of Amsterdam, The Netherlands
[4]Department of Intensive Care & Laboratory of Experimental Intensive Care and Anesthesiology
University of Insubria, Varese, Italy
[5]Department of Environment, Health and Safety
University of Foggia, Italy
[6]Department of Anesthesiology and Intensive Care Medicine
Saint Eloi University Hospital, Montpellier, France
[7]Department of Critical Care Medicine and Anesthesiology (SAR B)
Hospital Universitari Germans Trias I Pujol, Barcelona, Spain:
[8]Department of Anesthesiology
Medical University, Vienna, Austria
[9]Division Cardiac-, Thoracic-, Vascular Anesthesia and Intensive Care
[10]Department of Anesthesiology and Intensive Care Medicine
AZ Sint Jan Brugge-Oostende AV, Belgium
[11]Department of Anesthesiology
Ghent University Hospital, Belgium
[12]Department of Anesthesiology and Intensive Care Medicine
Mayo Clinic, USA

[13]Department of Anesthesiology
Massachusetts General Hospital, Harvard University, USA
[14]Department of Anesthesiology and Critical Care
Saint Michael's Hospital, University of Toronto, Canada
[15]Department of Anesthesiology and Critical Care
University Hospital, Uppsala, Sweden:
[16]Department of Medical Sciences, Section of Clinical Physiology
Tel Aviv Medical Center, Israel
[17]Department of Anesthesiology and Critical Care
University of Bonn, Germany
[18]Department of Anesthesiology and Intensive Care Medicine
Hôpitaux Universitaires de Genève, Switzerland
[19]Service d'Anesthésiologie
University of Aachen, Germany
[20]Department of Anesthesiology
University of Istanbul, Turkey
[21]Department of Anesthesiology and Intensive Care Medicine
Città della Salute e della Scienza, Turin, Italy
[22]Department of Anesthesiology
Semmelweis Egyetem, Hungary
[23]Aneszteziológiai és Intenzív Terápiás Klinika
Sheffield Teaching Hospitals, United Kingdom
[24] Operating Services, Critical Care and Anaesthesia (OSCCA)
University Hospital Dresden, Germany
[25]Center for Evidence-based Healthcare
University of Genoa, Genoa, Italy
[26]Dept. of Surgical Sciences and Integrated Diagnostics

Correspondence:
Marcelo Gama de Abreu, MSc MD PhD DESA
Principal Investigator
University Hospital Dresden
Department of Anesthesiology
and Intensive Care Medicine
Fetscherstr. 74
01307 Dresden
Germany
E–mail: mgabreu@uniklinikum-dresden.de

TABLE OF CONTENTS

1. LIST OF ABBREVIATIONS AND RELEVANT DEFINITIONS 6
2. SUMMARY 7
3. INTRODUCTION AND RATIONALE 8
3.1 Postoperative pulmonary complications 8
3.2 Ventilator–associated lung injury 8
3.3 Postoperative pulmonary complications and mechanical ventilation 8
3.4 Mechanical ventilation in obese patients 8
4. OBJECTIVES AND HYPOTHESIS 10
4.1 Objectives 10
4.2 Hypothesis 10
5. STUDY DESIGN 11
6. STUDY POPULATION 12
6.1 Population (base) 12
6.2 Inclusion criteria 12
6.3 Exclusion criteria 12
6.4 Sample size calculation 13
7. METHODS 14
7.1 Study parameters/endpoints 14
7.1.1 Main study parameter/endpoint 14
7.1.2 Secondary study parameters/endpoints 14
7.1.3 Other study parameters 14
7.2 Study procedures 14
7.2.1 Randomization 14
7.2.2 Mechanical ventilation and recruitment maneuvers 15
7.2.3 Standard procedures 17
7.2.4 Data to be collected 18
7.2.5 Blood and urine samples 22
8. STATISTICAL ANALYSIS 23
8.1 Descriptive statistics 23
8.2 Analysis 23
8.3 Data Safety Management Board (DSMB) 24
9. ETHICAL CONSIDERATIONS 26
9.1 Regulation statement 26
10. ADMINISTRATIVE ASPECTS AND PUBLICATION 27
10.1 Handling and storage of data and documents 27
11. REFERENCES 28
12. APPENDICES 30

1. LIST OF ABBREVIATIONS AND RELEVANT DEFINITIONS

AE	Adverse Event
ALI	Acute Lung Injury
AR	Adverse Reaction
ARDS	Acute Respiratory Distress Syndrome
BMI	Body Mass Index
CA	Competent Authority
COPD	Chronic Obstructive Pulmonary Disease
CPAP	Continuous Positive Airway Pressure
EU	European Union
ICU	Intensive Care Unit
NPPV	Noninvasive Positive Pressure Ventilation
OSA	Obstructive Sleep Apnea
PEEP	Positive end–expiratory pressure
PPC	Postoperative Pulmonary Complication
VALI	Ventilator–associated lung injury

2. SUMMARY

Rationale

Postoperative respiratory failure, particularly after surgery under general anesthesia, adds to the morbidity and mortality of surgical patients. Anesthesiologists inconsistently use positive end–expiratory pressure (PEEP) and recruitment maneuvers in the hope that this may improve oxygenation and protect against postoperative pulmonary complications (PPCs), especially in obese patients. While it is uncertain whether a strategy that uses higher levels of PEEP with recruitment maneuvers truly prevents PPCs in these patients, use of higher levels of PEEP with recruitment maneuvers could compromise intra–operative hemodynamics.

Objectives

To compare a ventilation strategy using higher levels of PEEP with recruitment maneuvers with one using lower levels of PEEP without recruitment maneuvers in obese patients at an intermediate–to–high risk for PPCs.

Hypotheses

An intra–operative ventilation strategy using higher levels of PEEP and recruitment maneuvers, as compared to ventilation with lower levels of PEEP without recruitment maneuvers, prevents PPCs in obese patients at an intermediate–to–high risk for PPC.

Study design

International multicenter randomized controlled trial.

Study population

Obese patients with BMI ≥ 35 kg/m^2 at intermediate–to–high risk for PPCs scheduled for surgery under general anesthesia.

Main study parameters/endpoints

The primary endpoint is the proportion of patients with PPCs. Secondary endpoints include intra–operative complications, need for postoperative ventilatory support (invasive and/or non–invasive ventilation), need for unexpected ICU admission or ICU readmission, the number of hospital–free days and 90-day survival/mortality.

Nature and extent of the burden and risks associated with participation, benefit and group relatedness

In the intra–operative period, patients will not experience discomfort from either strategy because of general anesthesia. However, systemic hypotension could occur in the higher PEEP group, which would be treated with intravascular volume therapy and/or vasoactive drugs. If the hypothesis proves to be true, patients in the higher PEEP group could benefit from a lower incidence of PPCs. Blood samples will be collected via an existing intravenous catheter or via direct vein puncture, but always in combination with blood sampling for routine care; the amount of additional blood samples can be considered minimal.

3. INTRODUCTION AND RATIONALE

3.1 Postoperative pulmonary complications

Postoperative pulmonary complications, especially postoperative respiratory failure, add to the morbidity and mortality of surgical patients[1,2]. An ARISCAT score ≥ 26 is associated with an intermediate–to–high risk of postoperative pulmonary complications (PPCs), independent of BMI[3].

3.2 Ventilator–associated lung injury

Even though mechanical ventilation is a life–saving strategy in patients with respiratory failure and frequently necessary during general anesthesia, both experimental[4-6] and clinical[7-9] studies show that mechanical ventilation has the potential to aggravate or even initiate lung injury (so–called ventilator–associated lung injury, VALI). Repetitive collapse/reopening of lung units (atelectrauma) and overdistension of lung units (volutrauma) are possible mechanisms underlying VALI[10-12]. While positive end–expiratory pressure (PEEP) can minimize atelectrauma, lower tidal volumes are thought to reduce volutrauma. One meta–analysis showed that use of lower tidal volumes is associated with a better outcome for patients with uninjured lungs[13]. This study included both surgery patients who underwent mechanical ventilation for general anesthesia as well as critically ill patients who required longer mechanical ventilation. Notably, a more recent meta-analysis showed a decrease in lung injury development, pulmonary infection and atelectasis in patients receiving intraoperative mechanical ventilation with both lower tidal volumes and higher levels of PEEP [14].

3.3 Postoperative pulmonary complications and mechanical ventilation

Mechanical ventilation is frequently required in patients undergoing surgery. Our group has shown that an intraoperative ventilation strategy with lower tidal volume and positive end–expiratory pressure (PEEP) may improve postoperative lung function[15] and even outcome[16] in patients undergoing open abdominal surgery. More recently, a similar mechanical ventilation strategy was shown to reduce the incidence of PPCs and health care utilization in intermediate–to–high risk patients with a body mass index (BMI) < 35 kg/m^2 undergoing open abdominal surgery[17]. A recently concluded multicenter, international, randomized controlled trial could not show a protective effect of higher PEEP and recruitment against PPCs in patients with a BMI < 40 kg/m^2 [18]. However, most of the patients included were not obese, i.e. had a BMI < 30 kg/m^2. Therefore, these results cannot be extrapolated to obese patients.

3.4 Mechanical ventilation in obese patients

According to Pelosi and Gregoretti[19], body mass is an important determinant of respiratory function before and during anesthesia not only in morbidly, but also in moderately obese patients. The impairment can manifest as (a) reduced lung volume with increased atelectasis and/or small airway closure; (b) derangements in respiratory system, lung and chest wall

compliance and increased resistance; and (c) moderate to severe hypoxemia. These physiological alterations are more marked in obese patients with hypercapnia or obstructive sleep apnea syndrome. In order to avoid or reduce such complications, PEEP levels should, theoretically, be set higher in obese than in non-obese patients. However, there is as of yet no clinical evidence supporting such an approach. In fact, an observational study conducted in 28 centers in France revealed that most patients undergoing general surgery, including obese ones, were ventilated with low (≤ 4 cmH_2O) or even without PEEP, even though average PEEP was higher in obese compared to non-obese patients [20]. While there is uncertainty about the lung protective effects of PEEP, there is considerable evidence that tidal volumes in the range of 6 to 8 ml/kg of predicted body weight (PBW) is protective also in non-injured lungs, independent of BMI[13,14,17].

4. OBJECTIVES AND HYPOTHESIS

4.1 Objectives

The proposed randomized controlled trial aims at comparing the effects of higher levels of PEEP with recruitment maneuvers versus lower levels of PEEP without recruitment maneuvers on pulmonary and extra-pulmonary outcome measures during general anesthesia for surgery, as well as determining the length of hospital stay for obese patients at intermediate–to–high risk for PPCs and undergoing mechanical ventilation with lower tidal volumes.

4.2 Hypothesis

We hypothesize that an intra–operative ventilation strategy using higher levels of PEEP and recruitment maneuvers, as compared to ventilation with lower levels of PEEP without recruitment maneuvers, prevents PPCs in obese patients at a intermediate–to–high risk for PPCs.

5. STUDY DESIGN

Multicenter, international, randomized controlled trial on obese patients with BMI ≥ 35 kg/m^2 at intermediate–to–high risk for PPCs and scheduled for general anesthesia because of surgery.

6. STUDY POPULATION

6.1 Population (base)

We intend to recruit obese patients with BMI ≥ 35 kg/m^2 consecutively scheduled for surgery in the participating hospitals during a period of 2 years. Currently, we expect about 90 centers to participate in the trial. Surgical patients in these centers will be screened daily. Demographic data on screened patients, regardless of enrollment criteria match, will be recorded (registry). We will randomize 2013 patients admitted to the participating centers and expect each participating center to randomize at least 24 patients who meet all inclusion criteria.

6.2 Inclusion criteria

- Patient scheduled for surgery under general anesthesia
- Intermediate-to-high risk for PPCs following surgery, according to the ARISCAT risk score (≥ 26) (see APPENDIX i)
- BMI ≥ 35 kg/m^2
- Expected duration of surgery ≥ 2 h

6.3 Exclusion criteria

- Age < 18 years
- Previous lung surgery (any)
- Persistent hemodynamic instability, intractable shock (considered hemodynamically unsuitable for the study by the patient’s managing physician)
- History of previous severe chronic obstructive pulmonary disease (COPD) (non–invasive ventilation and/or oxygen therapy at home, repeated systemic corticosteroid therapy for acute exacerbations of COPD)
- Recent immunosuppressive medication (patients receiving chemotherapy or radiation therapy up to two months prior to surgery)
- Severe cardiac disease (New York Heart Association class III or IV, acute coronary syndrome or persistent ventricular tachyarrhythmias)
- Invasive mechanical ventilation longer than 30 minutes (e.g., general anesthesia for surgery) within last 30 days
- Pregnancy (excluded by anamneses and/or laboratory analysis)
- Prevalent acute respiratory distress syndrome expected to require prolonged postoperative mechanical ventilation
- Severe pulmonary arterial hypertension, defined as systolic pulmonary artery pressure > 40 mmHg
- Intracranial injury or tumor
- Neuromuscular disease (any)

- Need for intraoperative prone or lateral decubitus position
- Need for one-lung ventilation
- Cardiac surgery
- Neurosurgery
- Planned reintubation following surgery
- Enrolled in other interventional study or refusal of informed consent

6.4 Sample size calculation

Initial sample size calculation was based on our primary hypothesis and primary study outcome, and was informed by data collected during a multicenter Spanish trial (ARISCAT) (see APPENDIX i) and a single-center, relatively small study reporting the effects of intraoperative higher PEEP and recruitment maneuvers on the incidence of postoperative desaturation, chest infection and bronchospasm in obese patients who underwent laparoscopic bariatric surgery [21].

These calculations indicated that 356 patients were required per group, assuming a two–sided significance level of 0.05 and a power of 80%, to detect the expected difference in postoperative pulmonary complications between the higher PEEP group of 30% and the lower PEEP group of 40% (risk ratio of 0.75). Assuming a dropout rate of 5%, **a total of 748 patients** (n=374 per group) were planned to be included into the study.

Sample size was re-estimated based on recommendations of the DSMB after data on the first 618 patients revealed that the incidence of the collapsed composite outcome was considerably lower than expected. In the revised calculations, the control group incidence was assumed to be 0.20 (instead of 0.40), and sample size was calculated to have 80% power at the overall 0.05 significance level to detect a relative risk (as originally planned) of 0.75. This design requires a maximum of 1912 patients, adjusting for the interim monitoring for efficacy and futility as described in section 8.2. Assuming a dropout rate of 5%, a total of 2013 patients need to be included into the study. East 6.0 interim monitoring software (Cytel, Cambridge, MA, USA) was used.

7. METHODS

7.1 Study parameters/endpoints

7.1.1 Main study parameter/endpoint

- Postoperative pulmonary complications

7.1.2 Secondary study parameters/endpoints

- Intra–operative complications, i.e., complications related to the ventilation strategy (for example: de–saturation, defined as $SpO_2 \leq 92\%$, for > 1 min; hypotension during recruitment maneuvers, as defined by systolic arterial pressure < 90 mmHg for > 2 min)
- Need for postoperative ventilatory support (invasive or non–invasive ventilation)
- Unexpected need for ICU admission (i.e., before surgery the patient is not scheduled for ICU admission, but is admitted eventually) or ICU readmission within 30 days
- Need for hospital readmission within 30 days
- Hospital–free days at day 90
- Mortality at day 90
- Postoperative extra–pulmonary complications
- Postoperative wound healing [29]

7.1.3 Other study parameters

- Systemic levels of markers of (pulmonary) inflammation
- Systemic levels of markers of lung injury
- Systemic levels of markers of distal organ injury

7.2 Study procedures

Surgical patients in participating centers will be considered eligible if they fulfill the entry criteria. Eligible patients will be screened, their demographic data recorded (registry: age, gender, type of surgery), and those without exclusion criteria will be randomized. In total, 748 patients will be included.

Patient Consent

All patients or legal guardians must provide written informed consent according to local regulations before inclusion in the study.

7.2.1 Randomization

Randomization procedure

Randomization will be performed using a dedicated website and will be balanced per center. Randomization must take into account the risk of developing pulmonary complications (ARISCAT, see APPENDIX i.) to assure a balance for both intermediate and high–risk subgroups.

Randomization arms

Central randomization with the use of a permutated-block randomization list (block length 6) will be used. Before surgery patients will be randomly assigned 1:1 to mechanical ventilation with PEEP of 4 cmH_2O without recruitment maneuvers (the "lower PEEP level") *or* mechanical ventilation with PEEP of 12 cmH_2O with the use of recruitment maneuvers (the "higher PEEP level"). If desaturation, defined as $SpO_2 \leq 92\%$ for > 1 min, occurs, rescue is performed according to the sub-section "Rescue Therapy". Both strategies were chosen taking into account a recent national survey in France[22], most recent randomized clinical trials on mechanical ventilation of obese patients undergoing surgery[23-25] and expert consensus, which was obtained during a meeting of the Respiration Subcommittee at Euroanaesthesia 2013 in Barcelona, Spain.

At each site at least two investigators will be involved: one who will be aware of the allocated intervention and collect intra-operative data; the other who will remain blinded to the intra-operative interventions and evaluate the outcomes, scoring postoperative pulmonary and extrapulmonary complications.

7.2.2 Mechanical ventilation and recruitment maneuvers

Mechanical ventilation

Mechanical ventilation will be administered through the anesthesia ventilators in use in each individual center participating in the study. Patients will undergo volume–controlled mechanical ventilation with the lowest possible oxygen fraction (but at least 0.4) to maintain an oxygen saturation of 93% or higher, an inspiratory to expiratory ratio (I:E) of 1:2 and a respiratory rate adjusted to normocapnia (end–tidal carbon dioxide partial pressure between 35 and 45 mmHg). It is left to the discretion of the attending anesthesiologist to use a higher fraction of inspired oxygen.

Tidal volume will be set to 7 ml/kg Ideal Body Weight (IBW). The IBW is calculated according to a predefined formula: 50 + 0.91 x (centimeters of height – 152.4) for males and 45.5 + 0.91 x (centimeters of height – 152.4) for females. Tidal volume throughout this protocol refers to the actual inspired tidal volume in the ventilator circuit.

The PEEP level is selected according to the randomization group, i.e. 4 cmH_2O with the lower PEEP level, and 12 cmH_2O with the higher PEEP level.

Recruitment maneuver

The recruitment maneuver, as part of the protective strategy, will be performed directly after induction of anesthesia, after any disconnection from the mechanical ventilator, every one hour during surgery, and before end of surgery, in a hemodynamically stable situation as

judged by the anesthesiologist. The recruitment maneuver is not easily applied with available anesthesia ventilators since not all machines have an inspiratory hold function and adequate facilities. To obtain standardization among centers, recruitment maneuvers will be performed in volume–controlled ventilation, as follows:

1. Set peak inspiratory pressure limit to 55 cmH_2O
2. Set tidal volume to 7 ml/kg IBW and respiratory rate to 6 or higher breaths/min, while PEEP is 12 cmH_2O (or higher if during rescue)
3. Set inspiratory to expiratory ratio (I:E) to 1:1
4. Increase tidal volume in steps of 4 ml/kg IBW until plateau pressure reaches 40 – 50 cmH_2O
5. If the maximum tidal volume allowed by the anesthesia ventilator is achieved and the plateau pressure is lower than 40 cmH_2O, increase the PEEP as needed, but maximum 20 cmH_2O
6. Allow three breaths while maintaining plateau pressure of 40 – 50 cmH_2O
7. Set respiratory rate, I:E, inspiratory pause and tidal volume back to pre-recruitment values, while maintaining PEEP at 12 cmH_2O (or higher if during rescue)
8.

Protocol deviation

Anesthesiologists may deviate from the ventilation protocol at any time if concerns about the patient's safety arise, or upon the surgeon's request.

If one of the following complications occurs and is unresponsive to specific conventional therapy, PEEP may be modified according to the anesthesiologist's judgment:

- Systolic arterial pressure lower than 90 mmHg for more than three minutes and unresponsive to fluids and/or vasoactive drugs
- New arrhythmias unresponsive to the treatment suggested by the Advanced Cardiac Life Support Guidelines [26]
- Need for a dosage of vasoactive drugs at the tolerance limit, as judged by the anesthesiologist
- Need of massive transfusion, defined as replacement of >100% blood volume in 24 hours or >50% of blood volume in 4 hours (adult blood volume is approximately 70 mL/kg), to maintain Hct > 21% (Hb > 7 mg/dl)
- Life-threatening surgical complication

Rescue therapy (with the lower PEEP level)

In case of oxyhemoglobin desaturation ($SpO_2 \leq 92\%$) of a patient in the lower PEEP level group, after exclusion airway problems, auto-PEEP hemodynamic impairment, and ventilator malfunction, a rescue strategy is provided according to the following table:

Step	FIO_2	PEEP
1	0.5	4 cmH_2O
2	0.6	4 cmH_2O
3	0.7	4 cmH_2O
4	0.8	4 cmH_2O
5	0.9	4 cmH_2O
6	1.0	4 cmH_2O
7	1.0	5 cmH_2O
8	1.0	6 cmH_2O
9	1.0	7 cmH_2O (+RM)
(+RM), recruitment maneuver optional		

Rescue therapy (with the higher PEEP level)

In case of desaturation ($SpO_2 \leq 92\%$) of a patient in the higher PEEP level group, **it is crucial to exclude hemodynamic impairment as a possible cause**. Also, airway problems, auto-PEEP, and ventilator malfunction must be ruled out as possible causes. Provided those factors are excluded, a rescue strategy is allowed according to the following table:

Step*	FIO_2	PEEP
1	0.4	14 cmH_2O (+RM)
2	0.4	16 cmH_2O (+RM)
3	0.4	18 cmH_2O (+RM)
4	0.5	18 cmH_2O
5	0.6	18 cmH_2O
6	0.7	18 cmH_2O
7	0.8	18 cmH_2O
8	0.9	18 cmH_2O
9	1.0	18 cmH_2O
10	1.0	20 cmH_2O (+RM)
(+RM), recruitment maneuver optional<br>**At any step: If SpO_2 deteriorates further in an otherwise hemodynamic stable patient, consider reducing the PEEP to 10 and then 8 cmH_2O*		

7.2.3 Standard procedures

Start of surgery will be defined as the moment of incision for open surgery or insertion of trocars for laparoscopic surgery. End of surgery is the moment of closure of the surgical wound.

Routine general anesthesia, post–operative pain management, physiotherapeutic procedures and fluid management will be performed in the intra–operative and/or post–

operative period according to each center's specific expertise and clinical routine. However, the investigators suggest:

- To use inhalational isoflurane, desflurane or sevoflurane, intravenous propofol, remifentanil or sufentanil, and cis-atracurium, atracurium, vecuronium, or rocuronium (as required)
- To use balanced solution of prostigmine, or neostigmine and atropine or glycopyrrolate for reversal of muscle relaxation, guided by neuromuscular function monitoring (for example train-of-our)
- To perform postoperative pain management in order to achieve a VAS pain score below 3. Regional or neuraxial analgesia should be used whenever indicated
- To use physiotherapy by early mobilization, deep breathing exercises with and without incentive spirometry and stimulation of cough in the postoperative period
- To avoid fluid under and overload (according to the discretion of the anesthesiologist)
- To use invasive measurement of arterial blood pressure whenever indicated
- To use appropriate prophylactic antibiotics whenever indicated

Routine intra–operative monitoring should include noninvasive blood pressure measurements, pulse oximetry, end–tidal carbon dioxide fraction and electrocardiography. Every patient should receive at least one peripheral venous line to allow adequate fluid resuscitation during the study period. Nasogastric tubes, urinary bladder catheters and/or other intravenous catheters, as well as other, more invasive monitoring may be used according to local practice and/or guidelines.
Other procedures should follow the Safe Surgery Checklist (see www.who.int/patientsafety/safesurgery/en/index.html).

7.2.4 Data to be collected

Pre–operative variables

Pre–operative variables will be collected at the pre-anesthetic visit or before induction of general anesthesia:

- Gender and age; male + years
- Height and weight; kg + cm
- Physical status; according to the American Society of Anesthesiologists (ASA)
- Cardiac status: heart failure, according to the New York Heart Association (NYHA), coronary heart disease, according to Canadian Cardiovascular Society Classification (CCS), atrial flutter/fibrillation
- In patients without known obstructive sleep apnea (OSA), STOP-Bang score

- In patients with known OSA, apnea-hypopnea index (AHI)
- COPD with inhalation therapy and/or steroids; if yes: specify
- Respiratory infection in the last month; if yes: specify upper *or* lower respiratory infection
- Smoking status; never, former (at least three months prior) *or* current
- History of active cancer; if yes, specify type of cancer, classification + therapy
- History of diabetes mellitus; if yes: dietary treatment, oral medication *or* insulin therapy
- History of hypertension
- History of gastroesophageal reflux disease
- Cumulated Ambulation Score [27] (CAS) to evaluate mobility, see Appendix ii
- Alcohol status in the past 2 weeks; 0–2 drinks/day *or* > 2 drinks/day
- Use of antibiotics in the last 3 months; if yes: specify indication + drug
- Use of statins; if yes: specify type and dose
- Use of aspirin; if yes: specify dose
- Use of oral anti–diabetics; if yes: specify type and dose
- Use of noninvasive respiratory support; if yes: specify if CPAP or NPPV, duration and intensity
- Priority of surgery; elective, urgent, emergency
- Surgical procedure; visceral (biliary, gastric, pancreatic, liver colonic, rectal, other - specify), thoracic (not requiring one lung ventilation - specify), vascular (specify), orthopedic (hip, knee, other - specify), gynecologic (breast, uterus, other - specify), urologic (bladder, kidney, prostate, other – specify), *or* other
- Transfusion, packed red blood cells, fresh frozen plasma, plasma frozen within 24 hours (FP24), fibrinogen, cryoprecipitate, PPSB and platelets in the preceding six hours; if yes: specify type and number of transfused units/amount
- Actual organ function evaluation
  - Respiratory rate
  - SpO_2 (10min in room air, beach chair position); %
  - Bedside spirometry (FVC, FEV1) – not mandatory
  - Visual Analogue Scale (10 cm): evaluation for dyspnea, thoracic and abdominal rest and incident pain
  - Airway secretion score: ask patient to cough and subjectively evaluate presence and consistency of sputum; if yes: purulent *or* not
  - Chest X–ray – not mandatory
  - Noninvasive mean arterial pressure; mmHg
  - Heart rate; BPM

- Body temperature; ^{o}C
- BUN, Creatinine, AST, ALT, Bilirubin, Hb, Platelets, PT, PTT, WBC count

Induction variables

During anesthesia induction, patient's position and use of CPAP or NPPV will be documented.

Intra–operative variables

During the intra–operative period, the following variables will be recorded (variables are to be measured after induction, hourly and immediately before and after recruitment maneuvers):

- Duration of anesthesia procedure; from tracheal intubation to extubation *or* exit from operation room (in case patient remains on mechanical ventilation); minutes
- Duration of surgical procedure from incision to closure; minutes
- Operation classification; clean, clean–contaminated, contaminated *or* dirty according to Berard and Gandon [28]
- Surgical approach: laparoscopic surgery (specify intraabdominal pressure); assisted laparoscopic (specify intraabdominal pressure), open, conversion from laparoscopic to open
- Patient position during surgery: supine, Trendelenburg, reverse Trendelenburg, lithotomy, sitting position
- Types and total doses of anesthetics; inhalational, intravenous or balanced + dose
- Neuraxial anesthesia; if yes: specify epidural, plexus, peripheral
- Antibiotic prophylaxis; if yes: specify regimen
- Ventilator settings, hourly:
    - Peak and plateau pressures; cmH_2O
    - PEEP; cmH_2O
    - Tidal volume; ml
    - Respiratory rate
    - Inspiration to expiration ratio
    - Inspired oxygen fraction; %
    - Peripheral oxygen saturation; %
    - End–tidal fractions of CO_2; mmHg
- Noninvasive systolic, diastolic and mean arterial pressure; mmHg
- Vasoactive drugs; if yes: specify type and dose
- Heart rate
- Temperature at end of surgery; ^{0}C

- Fluid requirements (crystalloids, artificial colloids, and albumin; specify type and amount)
- Transfusion of packed red blood cells, fresh frozen, plasma frozen within 24 hours (FP24), fibrinogen, cryoprecipitate, PPSB and platelets in the preceding six hours; if yes: specify type and number of transfused units/amount
- Blood loss; ml for whole duration of surgery
- Urine output; ml for whole duration of surgery

Protocol adherence, specify if any deviation from the protocol:

- Hypotension (BPsys < 90mmHg) unresponsive to fluids and/or vasoactive drugs
- New arrhythmias unresponsive to intervention (according to ACLS-Guidelines)
- Need for a dosage of vasoactive drugs at the tolerance limit
- Need of massive transfusion (replacement of >50% of blood volume in 4 hours to maintain Hct > 21% (Hb > 7mg/dl)
- Life-threatening surgical complication (injury to the hemodynamic and respiratory system and brain, including major bleeding, tension pneumothorax, intracranial injury)
- Other reason, specify

Intra–operative complications possibly related to recruitment maneuvers:

- New hypotension (BPsys < 90mmHg or BPsys drop > 10mmHg, if BPsys < 90 before RM)
- New bradycardia (HR <50bpm or HR drop > 20%, if HR < 50 before RM)
- New hypoxemia (SpO_2 ≤ 92% or SpO_2 drop > 5%, if SpO_2 < 92% before RM)
- Other event, specify:

Post–operative variables

The patients will be assessed daily between the first and the fifth day after surgery as well as on the last day before discharge from hospital. Clinical data and the presence of pulmonary and extra–pulmonary postoperative complications will be scored, the date of development of any complication documented (for definitions, see APPENDIX iii.).

The documentation will adhere to the timetable in APPENDIX iv.

- Continuation of non–invasive or invasive mechanical ventilation outside of the operation room directly after surgery; if yes: specify indication and duration, hours
- Any new requirement of non–invasive CPAP or NPPV; if yes: specify indication, duration and intensity
- Any new requirement of invasive mechanical ventilation; if yes: specify indication, duration and intensity
- ICU stay directly postoperative; if yes: specify reason

- Any new admission or readmission to the ICU at any time in the post–operative period; if yes: specify reason
- Postoperative nausea and vomiting (PONV)
- Physiotherapy and breathing exercises
- CAS [27] to evaluate mobility, see Appendix ii
- Wound healing: impaired wound healing can be defined as an interruption in the timely and predictable recovery of mechanical integrity in the injured tissue[29]
- Surgical wound infection; if yes: specify location (superficial *or* deep, abscess, empyema or phlegmon [30])
- Return of bowel function
- Any need for anti–arrhythmic and vasoactive medication; if yes: specify
- Post–operative fluid requirements (crystalloids, artificial colloids, or albumin; specify type and amount since last assessment)
- Post–operative transfusion of packed red blood cells, fresh frozen plasma , plasma frozen within 24 hours (FP24), fibrinogen, cryoprecipitate, PPSB and platelets in the preceding six hours; if yes: specify type and number of units transfused since last assessment
- Respiratory rate and peripheral oxygen saturation in room air, beach chair position
- Presence or absence of airway secretion (ask patient to cough and subjectively evaluate presence and consistency of sputum; if yes: purulent *or* not)
- Noninvasive mean arterial pressure
- Heart rate (bpm)
- Tympanic temperature; ^{o}C
- VAS evaluation for dyspnea, thoracic and abdominal rest and incident pain
- Chest X–ray – not mandatory
- Bedside spirometry (FVC, FEV1) – not mandatory
- BUN, Creatinine, AST, ALT, Bilirubin, Hb, Platelets, PT, PTT, white blood cell count
- Date of hospital discharge

7.2.5 Blood and urine samples

Before, at the end of, and five days after surgery, blood samples (2 x 5 ml in EDTA, citrate and heparin) and urine samples will be collected and stored at –80°C for measurement of:

- Inflammatory mediators (cytokines, chemokines, other inflammatory proteins)
- Markers of lung injury (angiopoietin-2, surfactant proteins A and D)
- Specific markers of distal organ injury (e.g., cystatin C, NGAL)

8. STATISTICAL ANALYSIS

8.1 Descriptive statistics

Patient characteristics will be compared and described by appropriate statistics.

8.2 Analysis

The primary endpoint is a collapsed composite of postoperative pulmonary complications occurring in the first five days after surgery. These complications include aspiration pneumonitis, bronchospasm, mild respiratory failure, moderate respiratory failure, severe respiratory failure, development of acute respiratory distress syndrome, pulmonary infection, atelectasis, pulmonary edema caused by cardiac failure, pleural effusion, pneumothorax and new pulmonary infiltrates (for definition, see Appendix iii). Patients who develop a least one of the above mentioned complications are considered as meeting the primary endpoint.

Interim analyses for efficacy and futility will be conducted at 50% (N=956), 75% (N=1434) and 100% (N=1912) of the planned enrollment, as needed, using a non-binding group sequential design with gamma spending functions (gamma = -4 for each of alpha and beta). The table below shows the alpha and beta spent over the trial, z-statistic boundaries for efficacy and futility, and boundary crossing probabilities under the alternative hypothesis (H1). The corresponding P-value boundaries for efficacy (futility in parentheses) at the 1st, 2nd and final looks, respectively, are P≤ 0.006 (P>0.82), P≤ 0.015 (P>0.35) and P ≤ 0.044 (P>0.044). The Figure below displays the z-statistic boundaries for efficacy/harm and futility as a function of accrued sample size.

Table: Z-statistic boundaries and boundary crossing probabilities (under H1);
Power=0.80, alpha=0.05, gamma spending functions (gamma = -4), p1=.20, p2=0.15

Look	Information Fraction	N	Cumulative Alpha spent	Cum. Beta spent	Z-efficacy	Z-futility	Boundary crossing probabilities under H1	
							Efficacy	Futility
1	0.5	956	0.006	0.024	≥2.75	<0.225	0.234	0.024
2	0.75	1434	0.018	0.071	≥2.432	<0.929	0.296	0.047
3	1	1912	0.05	0.2	≥2.012	<2.012	0.271	0.129

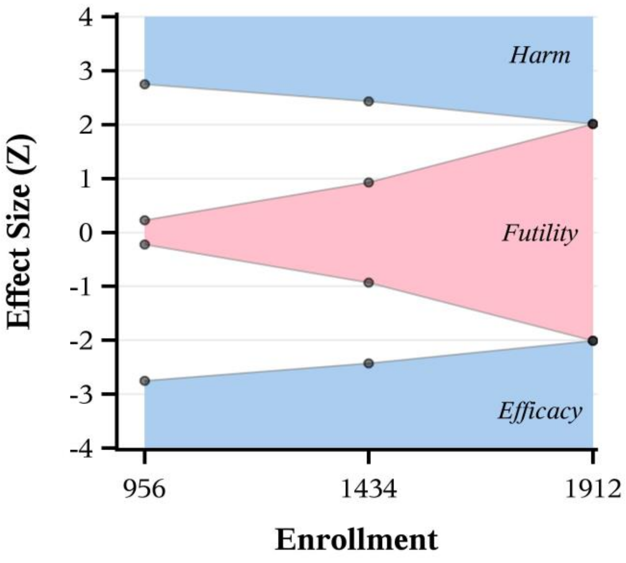

For the primary efficacy analysis rates of postoperative pulmonary complications will be compared between the two intervention groups and the odds ratio relative risks with corresponding 95% confidence levels interval will be calculated using logistic regression analysis.

With regard to the remaining parameters: Normally distributed variables will be expressed by their mean and standard deviation; non-normally distributed variables will be expressed by their medians and interquartile ranges. Categorical variables will be expressed as n (%). Student's *t*-test will be used to test groups of continuous normally distributed variables. Conversely, if continuous data is non-normally distributed, the Mann-Whitney-*U* test will be used. Categorical variables will be compared with the Chi–square test, Fisher's exact tests or, where appropriate, as relative risks. Time dependent data will be analyzed using a proportional hazard model adjusted for possible imbalances of patients' baseline characteristics.

Data analyses will follow an a priori documented statistical analysis plan which will be finalized before the end of data collection. By definition, only the analysis of the primary outcome is confirmatory, all other analyses are exploratory. The analysis strategy follows the intention-to-treat principle. The analysis will be undertaken blinded by a statistical expert. Statistical significance is considered to be at a *p*–value of 0.05. Where appropriate, statistical uncertainty will be expressed by 95% confidence levels.

The analysis will be performed with SPSS.

8.3 Data Safety Management Board (DSMB)

The DSMB will be composed of five individuals, one of whom will be the chairperson.

- The DSMB will first convene after the first 100 patients
- Subsequently, the DSMB will attend videoconferences every six months
- All adverse events will be reported to the DSMB for review. All serious events will be reported within 24 hours after being received by the coordinating center. Non-serious events will be reported within one week of reception by the coordinating center (Appendix V)
- All unexpected study-related or possibly study-related adverse events will be reported to the DSMB. Adverse events include but are not limited to unexpected death, inadvertent extubation, development of hemodynamic compromise during a recruitment maneuver or PEEP adjustment, sudden hypoxemia, hypercarbia or a pneumothorax during changes in ventilator setting in either the control or treatment group (Appendix V)
- The DSMB will monitor the overall status of the trial: number of patients enrolled overall and per each center, adherence to protocol overall and per center and results of the interim analysis.
- The DSMB include any of the following individuals:
  - Prof. D. Sessler, Outcomes Research/ P77 Cleveland Clinic 9500 Euclid Avenue Cleveland, OH, USA, (chair)
  - Prof. J. Wiener-Kronish, Massachusetts General Hospital, Boston, MA, USA
  - Prof. J. Hunter, University of Liverpool, Liverpool, UK
  - Prof. J. L. Vincent, University Erasme, Brussels, Belgium
  - Prof. A. Hoeft, University Hospital of Bonn, Germany

9. ETHICAL CONSIDERATIONS

9.1 Regulation statement

This study will be conducted in accordance to the principles of the Declaration of Helsinki.

10. ADMINISTRATIVE ASPECTS AND PUBLICATION

10.1 Handling and storage of data and documents

All enrolled patients will receive a random patient identification code. The codebook will be stored digitally, encrypted with a double password, and as a hard copy under lock and key. All data will be stored for the length of the study and afterwards as required by local law or for further publication. All handling of personal data will comply with local law.

11. REFERENCES

1. Arozullah AM, Daley J, Henderson WG, Khuri SF: Multifactorial risk index for predicting postoperative respiratory failure in men after major noncardiac surgery. The National Veterans Administration Surgical Quality Improvement Program. Ann Surg 2000; 232: 242-53

2. Smetana GW, Lawrence VA, Cornell JE: Preoperative pulmonary risk stratification for noncardiothoracic surgery: systematic review for the American College of Physicians. Ann Intern Med 2006; 144: 581-95

3. Canet J, Gallart L, Gomar C, Paluzie G, Valles J, Castillo J, Sabate S, Mazo V, Briones Z, Sanchis J: Prediction of postoperative pulmonary complications in a population-based surgical cohort. Anesthesiology 2010; 113: 1338-50

4. Tremblay LN, Valenza F, Ribeiro SP, Li J, Slutsky AS: Injurious ventilatory strategies increases cytokines and c-fos expression in an isolated rat lung. J Clin Invest 1997; 99: 944-952

5. Dreyfuss D, Saumon G: Ventilator-induced lung injury. Am J Respir Crit Care Med 1998; 157: 294-323

6. Imai Y, Parodo J, Kajikawa O, de Perrot M, Fischer S, Edwards V, Cutz E, Liu M, Keshavjee S, Martin TR, Marshall JC, Ranieri VM, Slutsky AS: Injurious mechanical ventilation and end-organ epithelial cell apoptosis and organ dysfunction in an experimental model of acute respiratory distress syndrome. JAMA 2003; 289: 2104-2112

7. Ranieri VM, Suter PM, Tortorella C: Effect of mechanical ventilation on inflammatory mediators in patients with acute respiratory distress syndrome: a randomized controlled trial. JAMA 1999; 282: 54-61

8. Terragni PP, Rosboch G, Tealdi A, Corno A, Menaldo E, Davini O, Gandini G, Herrmann P, Mascia L, Quintel M, Slutsky AS, Gattinoni L: Tidal hyperinflation during low tidal volume ventilation in acute respiratory distress syndrome. Am J Respir Crit Care Med 2007; 175: 160-166

9. Terragni PP, Del Sorbo L, Mascia L, Urbino R, Martin EL, Birocco A, Faggiano C, Quintel M, Gattinoni L, Ranieri VM: Tidal volume lower than 6 ml/kg enhances lung protection: role of extracorporeal carbon dioxide removal. Anesthesiology 2009; 111: 826-835

10. dos Santos CC, Slutsky AS: Invited review: mechanisms of ventilator-induced lung injury: a perspective. J Appl Physiol 2000; 89: 1645-1655

11. dos Santos CC, Slutsky A: The contribution of biophysical lung injury to the development of biotrauma. Annu Rev Physiol 2006; 68: 585-618

12. Duggan M, Kavanagh BP: Pulmonary atelectasis: a pathogenic perioperative entity. Anesthesiology 2005; 102: 838-54

13. Serpa Neto A, Cardoso SO, Manetta JA, Pereira VG, Esposito DC, Pasqualucci Mde O, Damasceno MC, Schultz MJ: Association between use of lung-protective ventilation with lower tidal volumes and clinical outcomes among patients without acute respiratory distress syndrome: a meta-analysis. JAMA 2012; 308: 1651-9

14. Hemmes SN, Neto AS, Schultz MJ: Intraoperative ventilatory strategies to prevent postoperative pulmonary complications: a meta-analysis. Curr Opin Anaesthesiol 2013; 26: 126-33

15. Severgnini P, Selmo G, Lanza C, Chiesa A, Frigerio A, Bacuzzi A, Dionigi G, Novario R, Gregoretti C, de Abreu MG, Schultz MJ, Jaber S, Futier E, Chiaranda M, Pelosi P: Protective Mechanical Ventilation during General Anesthesia for Open Abdominal Surgery Improves Postoperative Pulmonary Function. Anesthesiology 2013: 1307-1321

16. Hemmes SN, Serpa Neto A, Schultz MJ: Intraoperative ventilatory strategies to prevent postoperative pulmonary complications: a meta-analysis. Curr Opin Anaesthesiol 2013; 26: 126-33

17. Futier E, Constantin JM, Paugam-Burtz C, Pascal J, Eurin M, Neuschwander A, Marret E, Beaussier M, Gutton C, Lefrant JY, Allaouchiche B, Verzilli D, Leone M, De Jong A, Bazin JE, Pereira B, Jaber S, Group IS: A trial of intraoperative low-tidal-volume ventilation in abdominal surgery. N Engl J Med 2013; 369: 428-37

18. Hemmes SN, Severgnini P, Jaber S, Canet J, Wrigge H, Hiesmayr M, Tschernko EM, Hollmann MW, Binnekade JM, Hedenstierna G, Putensen C, de Abreu MG, Pelosi P, Schultz MJ: Rationale and study design of PROVHILO - a worldwide multicenter randomized controlled trial on protective ventilation during general anesthesia for open abdominal surgery. Trials 2011; 12: 111

19. Pelosi P, Gregoretti C: Perioperative management of obese patients. Best Pract Res Clin Anaesthesiol 2010; 24: 211-25

20. Jaber S, Coisel Y, Marret E, Malinovsky JM, Bouaziz H: Ventilatory management during general anesthesia: A multicenter observational study, Annual Meeting of the American Society of Anesthesiologists, 2006

21. Talab HF, Zabani IA, Abdelrahman HS, Bukhari WL, Mamoun I, Ashour MA, Sadeq BB, El Sayed SI: Intraoperative ventilatory strategies for prevention of pulmonary atelectasis in obese patients undergoing laparoscopic bariatric surgery. Anesth Analg 2009; 109: 1511-6

22. Jaber S, Tassaux D, Sebbane M, Pouzeratte Y, Battisti A, Capdevila X, Eledjam JJ, Jolliet P: Performance characteristics of five new anesthesia ventilators and four intensive care ventilators in pressure-support mode: a comparative bench study. Anesthesiology 2006; 105: 944-52

23. Schumann R: Anaesthesia for bariatric surgery. Best Pract Res Clin Anaesthesiol 2011; 25: 83-93

24. Bein B, Scholz J: Anaesthesia for adults undergoing non-bariatric surgery. Best Pract Res Clin Anaesthesiol 2011; 25: 37-51

25. Aldenkortt M, Lysakowski C, Elia N, Brochard L, Tramer MR: Ventilation strategies in obese patients undergoing surgery: a quantitative systematic review and meta-analysis. Br J Anaesth 2012; 109: 493-502

26. Morrison LJ, Deakin CD, Morley PT, Callaway CW, Kerber RE, Kronick SL, Lavonas EJ, Link MS, Neumar RW, Otto CW, Parr M, Shuster M, Sunde K, Peberdy MA, Tang W, Hoek TL, Bottiger BW, Drajer S, Lim SH, Nolan JP, Advanced Life Support Chapter C: Part 8: Advanced life support: 2010 International Consensus on Cardiopulmonary Resuscitation and Emergency Cardiovascular Care Science With Treatment Recommendations. Circulation 2010; 122: S345-421

27. Kristensen MT, Jakobsen TL, Nielsen JW, Jorgensen LM, Nienhuis RJ, Jonsson LR: Cumulated Ambulation Score to evaluate mobility is feasible in geriatric patients and in patients with hip fracture. Dan Med J 2012; 59: A4464

28. Berard F, Gandon J: Postoperative Wound Infections: The Influence of Ultraviolet Irradiation of the Operating Room and of Various Other Factors. Ann Surg 1964; 160: 1-192

29. Franz MG, Steed DL, Robson MC: Optimizing healing of the acute wound by minimizing complications. Curr Probl Surg 2007; 44: 691-763

30. Mangram AJ, Horan TC, Pearson ML, Silver LC, Jarvis WR: Guideline for Prevention of Surgical Site Infection, 1999. Centers for Disease Control and Prevention (CDC) Hospital Infection Control Practices Advisory Committee. Am J Infect Control 1999; 27: 97-132

12. APPENDICES

APPENDIX i.

Table: Risk for PPC of Variables Selected for the Logistic Regression Model

	Bivariate Analysis	Multivariate Analysis*	β Coefficients	Risk Score§
	**OR (95% CI)**	**OR (95% CI)**		
	n = 1627	N = 1624		
Age (yr)				
≤ 50	1	1		
51 – 80	3.1 (1.5 – 6.7)	1.4 (0.6 - 3.3)	0.331	3
> 80	8.8 (3.8 – 20.3)	5.1 (1.9 - 13.3)	1.619	16
Preoperative SpO_2, %				
≥ 96	1	1		
91 – 95	3.1 (1.8 - 5.3)	2.2 (1.2 - 4.2)	0.802	8
≤ 90	15.2 (7.2 – 32.5)	10.7 (4.1 - 28.1)	2.375	24
Respiratory infection in the last month	6.1 (3.4- 11.1)	5.5 (2.6 - 11.5)	1.698	17
Preoperative anemia (≤ 10 g/dL.)	4.4 (2.4 – 8.01)	3.0 (1.4 - 6.5)	1.105	11
Surgical incision				
Peripheral	1	1		
Upper abdominal	6.9 (4.0 – 11.9)	4.4 (2.3 - 8.5)	1.480	15
Intrathoracic	16.9 (8.4 - 34.1)	11.4 (4.9 - 26.0)	2.431	24
Duration of surgery, h				
≤ 2	1	1		
> 2 to 3	6.1 (3.2 – 11.3)	4.9 (2.4 - 10.1)	1.593	16
> 3	11.2 (6.3 – 20.1)	9.7 (4.7 - 19.9)	2.268	23
Emergency procedure	2.1 (1.2 - 3.7)	2.2 (1.04 - 4.5)	0.768	8

Abbreviations: CI, confidence interval; OR, odds ratio; SpO_2, oxyhemoglobin saturation by pulse

High or intermediate risk for postoperative pulmonary complications following surgery: ARISCAT risk score ≥ 26

APPENDIX ii.

Cumulated Ambulation Score (CAS)

The patient is assessed on the following functions:

. Transfer from supine-to-sitting-to-supine
. Transfer from sitting-to-standing-to-sitting (from armchair)
. Walking (with appropriate walking aid)

Each function is scored as follows:

. Able to perform function independently – 2
. Only able to perform function with assistance from one or two people – 1
. Unable to perform function despite assistance from two people – 0

The CAS is calculated as the sum of values on a given day.

APPENDIX iii.

DEFINITIONS of pulmonary post–operative complications

- Aspiration pneumonitis:

  Defined as respiratory failure after the inhalation of regurgitated gastric contents

- Bronchospasm:

  Defined as newly detected expiratory wheezing treated with bronchodilators

- Mild respiratory failure:

  PaO2 < 60 mmHg or SpO2 < 90% in room air during at least 10 min air but responding to supplemental oxygen (excluding hypoventilation)

- Moderate respiratory failure:

  PaO2 < 60 mmHg or SpO2 < 90% despite supplemental oxygen (excluding hypoventilation)

- Severe respiratory failure:

  Need for non–invasive or invasive mechanical ventilation (excluding hypoventilation)

- ARDS:

  Mild, moderate or severe according to the Berlin definition:

- Pulmonary infection:

  Defined as new or progressive radiographic infiltrate plus at least two of the following: antibiotic treatment, tympanic temperature > 38ªC, leukocytosis or leucopenia (WBC count < 4,000cells/mm3 or > 12,000cells/mm3) and/or purulent secretions

- Atelectasis:

  Suggested by lung opacification with shift of the mediastinum, hilum, or hemidiaphragm towards the affected area, and compensatory overinflation in the adjacent nonatelectatic lung

- Cardiopulmonary edema:

  Defined as clinical signs of congestion, including dyspnea, edema, rales and jugular venous distention, with the chest X–ray demonstrating increase in vascular markings and diffuse alveolar interstitial infiltrates

- Pleural effusion:

  Chest X–ray demonstrating blunting of the costophrenic angle, loss of the sharp silhouette of the ipsilateral hemidiaphragm in upright position, evidence of displacement of adjacent anatomical structures, or (in supine position) a hazy opacity in one hemithorax with preserved vascular shadows

- Pneumothorax:

  Defined as air in the pleural space with no vascular bed surrounding the visceral pleura

- New pulmonary infiltrates:

  Chest X–ray demonstrating new monolateral or bilateral infiltrate without other clinical signs

DEFINITIONS of extra–pulmonary post–operative complications

- Systemic inflammatory response syndrome (SIRS):

  Presence of two or more of the following findings: Body temperature < 36^0C or > 38^0C – Heart rate > 90 beats per minute – Respiratory rate > 20 breaths per minute or, on blood gas, a P_aCO_2 < 32 mmHg (4.3 kPa) – WBC count < 4,000 cells/mm3 or > 12,000 cells/mm3 or > 10% band forms

- Sepsis:

  SIRS in response to a confirmed infectious process; infection can be suspected or proven (by culture, stain, or polymerase chain reaction (PCR)), or a clinical syndrome pathognomonic for infection. Specific evidence for infection includes WBCs in normally sterile fluid (such as urine or cerebrospinal fluid (CSF), evidence of a perforated viscera (free air on abdominal x–ray or CT scan, signs of acute peritonitis), abnormal chest x–ray (CXR) consistent with pneumonia (with focal opacification), or petechiae, purpura, or purpura fulminans

- Severe sepsis:

  Sepsis with organ dysfunction, hypoperfusion, or hypotension

- Septic shock:

  Sepsis with refractory arterial hypotension or hypoperfusion abnormalities in spite of adequate fluid resuscitation; signs of systemic hypoperfusion may be either end-organ dysfunction or serum lactate greater than 4 mmol/dL. Other signs include oliguria and altered mental status. Patients are defined as having septic shock if they have sepsis plus hypotension after aggressive fluid resuscitation, typically upwards of 6 liters or 40 ml/kg of crystalloid

- Extra–pulmonary infection:

  Wound infection + any other infection

- Coma:

  Glasgow Coma Score $\leq$ 8 in the absence of therapeutic coma or sedation

- Acute myocardial infarction:

  Detection of rise and/or fall of cardiac markers (preferably troponin) with at least one value above the 99th percentile of the upper reference limit, together with: symptoms of ischemia, ECG changes indicative of new ischemia, development of pathological Q-waves, or imaging evidence of new loss of viable myocardium or new regional wall motion abnormality *Or:* sudden unexpected cardiac death, involving cardiac arrest with symptoms suggestive of cardiac ischemia (but death occurring before the appearance of cardiac markers in blood)

- Acute renal failure:

  Renal failure documented as follows: Risk: increased creatinine x1.5 or GFR decrease > 25% *or* urine output (UO) < 0.5 ml/kg/h x 6 hr – Injury: increased creatinine x2 or GFR decrease > 50% *or* UO < 0.5 ml/kg/h x 12 hr – Failure: increase creatinine x3 or GFR decrease > 75% *or* UO < 0.3 ml/kg/h x 24 hr or anuria x 12 hrs – Loss: persistent ARF = complete loss of kidney function > 4 weeks

- Disseminated intravascular coagulation:

  DIC score documented as follows: Platelet count < 50 (2 points), < 100 (1 point), or ≥ 100 (0 points) – D–dimer > 4 µg/ml (2 points), > 0.39 µg/ml (1 point) or ≤ 0.39 µg/ml (0 points) – prothrombin time > 20.5 seconds (2 points), > 17.5 seconds (1 point) or ≤ 17.5 seconds (0 points); if ≥ 5 points: overt DIC

- Gastro–intestinal failure:

  Gastro–intestinal bleeding

  Gastro–intestinal failure (GIF) score documented as follows: 0 = normal gastrointestinal function; 1 = enteral feeding with under 50% of calculated needs or no feeding 3 days after abdominal surgery; 2 = food intolerance (FI) *or* intra–abdominal hypertension (IAH); 3 = FI and IAH; and 4 = abdominal compartment syndrome (ACS)

- Hepatic failure:

  Hepatic failure during short term follow up (5 postoperative days) is considered as follows: Ratio of total bilirubin on postoperative day 5 to postoperative day 1 > 1,7 and ratio of international normalized ratio (INR) on postoperative day 5 to postoperative day 1 >1,0; during long term follow up (until postoperative day 90) at new presence of hepatic encephalopathy and coagulopathy (INR > 1,5) within 8 weeks after initial signs of liver injury (e.g. jaundice) without evidence for chronic liver disease

APPENDIX iv.

	Before surgery	During surgery (every hour)	End of surgery	Day 1	Day 2	Day 3	Day 4	Day 5	Day of hospital discharge	postop Day 90 (Phone call)
All patients eligible for the study										
**Screening and Randomization**										
Daily screening	X									
Demographic data (registry)	X									
Inclusion/Exclusion criteria	X									
ARISCAT Score	X									
Informed consent	X									
Randomization	X									
Randomized patients										
**Before Surgery**										
Demographic data	X									
History of previous disease	X									
Physical examination	X									
SpO_2 in room air	X									
Spirometry (facultative)	X									
Chest X-ray (facultative)	X									
Routine laboratory tests (facultative)	X									
Blood sampling (special markers, facultative)	X									
**During Surgery**										
Respiratory variables		X								
Hemodynamic variables		X								
**End of Surgery**										
Protocol dropout?			X							
Rescue therapy?			X							
Anesthesia variables			X							
Adverse Events (possibly related to RMs)			X							
Surgery variables			X							
Intraoperative fluid balance/transfusion requirement			X							
Continuation of mechanical ventilation after surgery			X							
Blood sampling (special markers, facultative)			X							
**Follow Up**										
Physical examination				X	X	X	X	X	X	
Actual recovery status (ICU stay, ventilatory support, mobility, bowel function, wound healing)				X	X	X	X	X	X	
SpO_2 in room air				X	X	X	X	X	X	
Spirometry (facultative)				X	X	X	X	X	X	
Chest X-ray (facultative)				X	X	X	X	X	X	
Routine laboratory tests (facultative)				X	X	X	X	X	X	
Blood sampling (special markers, facultative)								X		
Post–operative fluids/transfusion requirement				X	X	X	X	X		
Pulmonary complications (APPENDIX iii)				X	X	X	X	X	X	
Extra–pulmonary organ failure (APPENDIX iii)				X	X	X	X	X	X	
Adverse Events				X	X	X	X	X	X	
Date of hospital discharge									X	
Alive on day 90										X
Hospital-free days on day 90										X

APPENDIX v.

Adverse Events

1. Definitions

An adverse event (AE) is generally defined as any unfavorable and unintended diagnosis, symptom, sign (including an abnormal laboratory finding) syndrome or disease which either occurs during the study, having been absent at baseline, or if present at baseline, appears to worsen. Adverse events are to be recorded regardless of their relationship to the study intervention. With respect to intensity, adverse events are classified as follows:

- Mild some awareness of symptoms, but easily tolerated;
- Moderate symptoms causing enough discomfort to interfere with usual activity;
- Severe incapacitating event causing inability to work or to perform usual activity.

A Serious Adverse Event (SAE) is defined as any experience that suggests a significant hazard or side effect with respect to participants participating in a clinical study. This includes any experience which:

- is fatal or life threatening,
- is permanently disabling, i.e. incapacitating or interfering with the ability to resume normal life patterns,
- requires hospitalisation or prolongation of hospitalisation,
- requires other medically important circumstance (requires medical treatment to avoid one of the above mentioned conditions).

A special form of the SAE is the Suspected Unexpected Serious Adverse Reaction (SUSAR).

Adverse events possibly occurring during this study include but are not limited to unexpected death, inadvertent extubation, development of hemodynamic compromise during a recruitment maneuver or PEEP adjustment, sudden hypoxemia, hypercarbia or a pneumothorax during changes in ventilator setting in either the control or treatment group

2. Documentation of AEs and SAEs

All adverse events that occur between start of randomized intervention and 90-days follow up have to be immediately documented in the participant’s **electronic CRF**. AEs are classified as either serious or non-serious. Details about the clinical symptoms, clinical course and if medical treated, about therapies used should be provided as well as

information about relationship to study intervention, overall intensity and outcome of the adverse event.

3. Reporting of Adverse Events

Reporting responsibilities of the local coordinator

Any AE occurring after start of randomized study intervention will be reported. The participant will be followed until remission of the symptoms. When reporting an AE/SAE, a clinical narrative on each AE/SAE should be added, which gives the clinical context of the event and allows the DSMB to carefully review the AE/SAEs.

AEs are reported via electronic CRF within one week of reception by the coordinating center. SAEs as well as all related or possibly related events and all unexpected events are reported via electronic CRF within 24 hours after being received by the coordinating center. In case of a SUSAR (suspected unexpected serious adverse reaction) as well as death of study patients the SAE manager has to be informed additionally by email.

Management of AE/SAE

SAE manager: Ary Serpa Neto, MD, MSc, Amsterdam, The Netherlands

The SAE manager collects and judges the reports within predefined time frames. All related or possibly related events and all unexpected events are forwarded to the DSMB for further review. The DSMB members assess forwarded events and review all SAE and AE from all centers every 6 months or sooner if requested by them. If the DSMB rates an event to be related to the study therapy, the DSMB should inform the principle investigator.

In case of a SUSAR (suspected unexpected serious adverse reaction) as well as death of study patients the ethics committee of Dresden University will be informed within 7 days. Additionally a summary of all SAE will be provided twice per year.
