# Supplementary material for: Protective intraoperative ventilation with higher versus lower levels of positive end-expiratory pressure in obese patients (PROBESE): study protocol for a randomized controlled trial
Source: Trials. 2017 Apr 28;18:202. doi: 10.1186/s13063-017-1929-0 (PMC5410049; doi:10.1186/s13063-017-1929-0)
Supplement: Supplementary file 2 — Standard Protocol Items: Recommendations for Interventional Trials (SPIRIT) 2013 checklist: recommended items to address in a clinical trial protocol and related documents. (PDF 122 kb). [file 13063_2017_1929_MOESM2_ESM.pdf]

CONFIDENTIAL

The PROBESE Randomized Controlled Trial

Case Report Form
version 1.2.2

Protective Ventilation with Higher versus Lower PEEP during General Anesthesia for Surgery in Obese Patients

Patient Serial Number |__|__|__|__|__|__|
center patient

Local investigator 1 (intraoperative) ______________________________

Local investigator 2 (postoperative) ______________________________

Principal Investigator: Prof. Marcelo Gama de Abreu, Department of Anesthesiology and Intensive Care Medicine, University of Dresden

Contact: Thomas Bluth, Department of Anesthesiology and Intensive Care Medicine, University of Dresden, probese@peg-dresden.de

1. Inclusion Criteria

	yes	no
Patient scheduled for surgery under general anesthesia	☐	☐
Intermediate-to-high risk for PPCs following surgery, **ARISCAT risk score ≥ 26**	☐	☐
BMI ≥ 35 kg/m^2	☐	☐
Expected duration of surgery ≥ 2 h	☐	☐

2. Exclusion Criteria

	yes	no
Previous lung surgery (any)	☐	☐
Persistent hemodynamic instability, intractable shock (considered hemodynamically unsuitable for the study by the patient's managing physician)	☐	☐
History of previous severe chronic obstructive pulmonary disease (COPD) (non–invasive ventilation and/or oxygen therapy at home, repeated systemic corticosteroid therapy for acute exacerbations of COPD)	☐	☐
Recent immunosuppressive medication (patients receiving chemotherapy or radiation therapy up to two months prior to surgery)	☐	☐
Severe cardiac disease (New York Heart Association class III or IV, acute coronary syndrome or persistent ventricular tachyarrhythmia)	☐	☐
Invasive mechanical ventilation longer than 30 minutes (e.g., general anesthesia for surgery) within last 30 days	☐	☐
Pregnancy (excluded by anamneses and/or laboratory analysis)	☐	☐
Prevalent acute respiratory distress syndrome expected to require prolonged postoperative mechanical ventilation	☐	☐
Severe pulmonary arterial hypertension, defined as systolic pulmonary artery pressure > 40 mmHg	☐	☐
Intracranial injury or tumor	☐	☐
Neuromuscular disease (any)	☐	☐
Need for intraoperative prone or lateral decubitus position	☐	☐
Need for one-lung ventilation	☐	☐
Cardiac surgery or neurosurgery	☐	☐
Planned reintubation following surgery	☐	☐
Enrolled in other interventional study or refusal of informed consent	☐	☐
**Patient excluded from the study?**	☐	☐

Investigator _______________ Signature _____________

3. ARISCAT Score (modified according to study design)

			Points			Points			Points
Age	≤ 50	☐	0	51-80	☐	3	> 80	☐	16
Preoperative SpO_2 [%]<br>10 min in room air, beach chair position	≥ 96	☐	0	91-95	☐	8	≤ 90	☐	24
Respiratory Infection (last month)	No	☐	0	Yes	☐	17			
Preoperative Anemia<br>(Hb ≤ 6,2 mmol/l or ≤10 g/dl)	No	☐	0	Yes	☐	11			
Emergency procedure	No	☐	0	Yes	☐	8			
Surgical Incision	peripheral	☐	0	upper abdominal	☐	15			
Planned duration of surgery [hr]				> 2-3	☐	16	> 3	☐	23
**Total Risk Score**		☐		+	☐		+	☐	= ☐

Investigator _______________ Signature _____________

4 Patient details

Written informed consent	yes ☐ no ☐	Date informed consent signed	/ / 20
Age [yrs]		Gender	male ☐ female ☐
Height [cm]		Weight [kg]	
Waist/Hip Ratio according to WHO (definition page 34)			

5 History of previous disease

ASA Score [1-5]			
Cumulated Ambulation Score (page 34) [0-6]:			
Heart failure	yes ☐ no ☐	if yes	NYHA Score [1-4]:
Coronary heart disease	yes ☐ no ☐	if yes	CCS Score [0-4]:
Atrial flutter / fibrillation	yes ☐ no ☐	if yes	acute ☐ paroxysmal ☐ chronic ☐
Obstructive sleep apnea	yes ☐ no ☐	if yes	Apnea/Hypopnea Index [events/hr]:
		if no	STOP-Bang Score (page 34) [0-8]:
COPD	yes ☐ no ☐	if yes	steroids use yes ☐ no ☐
			inhalation therapy yes ☐ no ☐
Respiratory infection within last month	yes ☐ no ☐	if yes	upper ☐ lower ☐ respiratory infection
Smoking status	never ☐ former (cessation >3months) ☐ current ☐		
Use of noninvasive ventilatory support	yes ☐ no ☐	if yes	CPAP ☐ NPPV ☐
			duration [hrs/day]: intensity [pressure level]:
Active cancer	yes ☐ no ☐	if yes	cancer type:
			actual cancer classification: T___M___N___
Diabetes mellitus	yes ☐ no ☐	if yes	dietary ☐ oral medication ☐ insulin ☐
	if oral medication, specify		type: dose [mg/day]:
Arterial hypertension	yes ☐ no ☐		
Gastroesophageal reflux	yes ☐ no ☐	if yes	events ≥1/day ☐ ≥1/week ☐ ≥1/month ☐
Alcohol status (past 2 weeks)	0-2 drinks/day ☐ >2 drinks/day ☐		
Use of antibiotics (last 3 months)	yes ☐ no ☐	if yes	indication:
Use of statins	yes ☐ no ☐	if yes	type: dose [mg/day]:
Use of aspirin	yes ☐ no ☐	if yes	dose [mg/day]:

Investigator _______________ Signature ______________

6.1 Actual organ function – mandatory measurements

SpO_2 beach chair position + 10 min in room air possible?	yes ☐ no ☐	if yes	SpO_2 [%]:
		if no	SpO_2 [%]: **and** FiO_2 [%] (page 35):
RR [/min]			
HR [/min]		ABP mean [mmHg]	
Temperature [°C]		tympanic ☐ axillar ☐ inguinal ☐ oral ☐ rectal ☐ other ☐	if other specify:
Airway secretion	yes ☐ no ☐	if yes	purulent ☐ not purulent ☐
VAS dyspnea [1-10cm]		VAS thoracic pain [1-10cm]	
VAS abdominal rest pain [1-10cm]		VAS abdominal incident pain [1-10cm]	

6.2 Non-mandatory measurements

**Spirometry**		**Laboratory tests**		
FVC [L]	FVC[% predicted]	Hb	mmol/l ☐	g/dl ☐
FEV_1 [L/1sec]	FEV_1 [% predicted]	WBC	GPt/L	
		Platelets	GPt/L	
**Chest X-ray** obtained	yes ☐ no ☐	PT	INR	
if yes		PTT	sec	
infiltrates	yes ☐ no ☐	Creatinine	µmol/l ☐	mg/dl ☐
pleural effusion	yes ☐ no ☐	BUN	mmol/l ☐	mg/dl ☐
atelectasis	yes ☐ no ☐	ALT	µmol/s*l ☐	U/L ☐
pneumothorax	yes ☐ no ☐	AST	µmol/s*l ☐	U/L ☐
cardiopulmonary edema	yes ☐ no ☐	Bilirubin	µmol/l ☐	mg/dl ☐

Randomization	Low PEEP without RM ☐	High PEEP with RM ☐

1 Anesthetic Overview
1.1 Induction

Duration of anesthesia [min]
from intubation to extubation (or exit from OR if on mechanical ventilation)

Antibiotic prophylaxis	yes ☐ no ☐	Central venous line	yes ☐ no ☐
Arterial line	yes ☐ no ☐	Cardiac output measurements	yes ☐ no ☐

Regional anesthesia yes ☐ no ☐ if yes
- epidural: thoracic ☐ lumbar ☐
- plexus: cervical ☐ brachial ☐ lumbar ☐
- peripheral nerve: upper ☐ lower ☐ extremity

other:

Use of NIV during induction yes ☐ no ☐ if yes CPAP ☐ NPPV ☐

Patient's position during induction — angle of head elevation 0-15° ☐ 15-30° ☐ 30-45° ☐ >45° ☐

1.2 Drugs, Fluids, Transfusion

			cumulative dose				*cumulative dose*
Analgetics	Alfentanyl	yes ☐		Anesthetics	Dexmedetomidine	yes ☐	
[mg]	Fentanyl	yes ☐		[mg]	Etomidate	yes ☐	
	Lidocaine	yes ☐			Ketamine	yes ☐	
	Morphine	yes ☐			Midazolam	yes ☐	
	NSAIDs	yes ☐			Propofol	yes ☐	
	Piritramide	yes ☐			Thiopental	yes ☐	
	Procaine	yes ☐			other	yes ☐	
	Remifentanil	yes ☐		if other	type:		
	Sufentanil	yes ☐			type:		
	other	yes ☐		Muscle	Atracurium	yes ☐	
if other	type:			Relaxants	Cis-Atracurium	yes ☐	
	type			[mg]	Mivacurium	yes ☐	
Vapors	Desflurane	yes ☐			Pancuronium	yes ☐	
[vol%*min]	Enflurane	yes ☐			Rocuronium	yes ☐	
	Halothane	yes ☐			Succinylcholine	yes ☐	
	Isoflurane	yes ☐			Vecuronium	yes ☐	
	Sevoflurane	yes ☐			other	yes ☐	
	other	yes ☐		if other	type:		
if other	type:				type:		

Investigator _______________ Signature _____________

			cumulative dose				*cumulative dose*
Artificial	HES	yes ☐	______	Crystalloids [ml]		yes ☐	
Colloids	Gelatine	yes ☐	______	Albumin [ml]		yes ☐	______
[ml]	Dextran	yes ☐					
Transfusion	PRBC	yes ☐	______	Vaso-	Dobutamine	yes ☐	______
[ml]	FFP	yes ☐	______	active	Dopamine	yes ☐	______
	FP24	yes ☐	______	Drugs	Epinephrine	yes ☐	______
	Fibrinogen [g]	yes ☐	______	[mg]	Norepinephrine	yes ☐	______
	Cryoprecipitate	yes ☐	______		Phenylephrine	yes ☐	______
	PPSB [IU]	yes ☐	______		other	yes ☐	______
	Platelets	yes ☐	______	if other	type:		______
	other	yes ☐	______		type:		______
if other	type:						

1.3 End of anesthesia

∑ Blood loss [ml]	∑ Urine output [ml]
Temperature [°C] at end of surgery	tympanic ☐ axillar ☐ inguinal ☐ oral ☐ rectal ☐<br>other ☐ if other specify:
Neuromuscular function monitored? yes ☐ no ☐ if yes	Residual curarization at extubation yes ☐ no ☐
Curarization antagonized? yes ☐ no ☐ if yes	sugammadex ☐ cholinesterase inhibitor ☐

Investigator _______________ Signature _____________

2 Surgical overview

Duration of surgery [min]
from incision to closure

			cumulative dose				cumulative dose
Transfusion	PRBC	yes ☐	______		FFP	yes ☐	______
**before**	FP24	yes ☐	______		Fibrinogen [g]	yes ☐	______
surgery	Cryoprecipitate	yes ☐	______		PPSB [IU]	yes ☐	______
(last 6hrs)	Platelets	yes ☐	______		other	yes ☐	______
[ml]				if other	type:		

Priority of surgery (definition see below) — elective ☐ urgent ☐ emergency ☐

Surgical wound classification (definition see below) — clean ☐ clean-contaminated ☐ contaminated ☐ dirty ☐

Surgical procedure — visceral ☐ thoracic ☐ vascular ☐ orthopedic ☐ gynecologic ☐ urologic ☐ other ☐
specify procedure:

Patient's position during surgery — supine ☐ trendelenburg ☐ reverse trendelenburg ☐ lithotomy ☐ seated ☐

Surgical approach —
laparoscopic ☐ if abdominal intraabdominal pressure [mmHg]
assisted laparoscopic ☐ if abdominal intraabdominal pressure [mmHg]
open ☐
conversion from laparoscopic to open ☐

3 Definitions

Surgical wound classification

Clean	Elective, not emergency, non-traumatic, primarily closed; no acute inflammation; no break in technique; respiratory, gastrointestinal, biliary and genitourinary tracts not entered.
Clean-contaminated	Urgent or emergency case that is otherwise clean; elective opening of respiratory, gastrointestinal, biliary or genitourinary tract with minimal spillage (e.g. appendectomy) not encountering infected urine or bile; minor technique break.
Contaminated	Non-purulent inflammation; gross spillage from gastrointestinal tract; entry into biliary or genitourinary tract in the presence of infected bile or urine; major break in technique; penetrating trauma <4 hours old; chronic open wounds to be grafted or covered.
Dirty	Purulent inflammation (e.g. abscess); preoperative perforation of respiratory, gastrointestinal, biliary or genitourinary tract; penetrating trauma >4 hours old.

Priority of surgery

Elective	Surgery that is scheduled in advance because it does not involve a medical emergency
Urgent	Surgery required within < 48 hrs
Emergency	Non-elective surgery performed when the patient's life or well-being is in direct jeopardy

Investigator _______________ Signature _____________

4 Protocol adherence

Any deviation from the protocol? yes ☐ no ☐ if yes

1) Hypotension (BPsys < 90mmHg) unresponsive to fluids and/or vasoactive drugs yes ☐
2) New arrhythmias unresponsive to intervention (according to ACLS-Guidelines) yes ☐
3) Need for a dosage of vasoactive drugs at the tolerance limit yes ☐
4) Need of massive transfusion (replacement of >50% of blood volume in 4 hours to maintain Hct ≥ 21% (Hb > 4,2 mmol/l or 7 g/dl) yes ☐
5) Life-threatening surgical complication (injury to the hemodynamic and respiratory system and brain, including major bleeding, tension pneumothorax, intracranial injury) yes ☐
6) Other reason, specify: yes ☐

Specify protocol deviation:

Could the protocol be continued? yes ☐ no ☐

5 Adverse events (AE) / severe adverse events (SAE)

Any adverse events yes ☐ no ☐ if yes specify according to table:

Event (details, including treatment)	Serious	Intervention	Recovery	Outcome
	yes ☐<br>no ☐	unrelated ☐<br>possible ☐<br>probable ☐<br>unassessable ☐	mild ☐<br>moderate ☐<br>severe ☐<br>unassessable ☐	resolved - no sequelae ☐<br>resolved - sequelae ☐<br>unresolved ☐<br>death ☐<br>unknown ☐
	yes ☐<br>no ☐	unrelated ☐<br>possible ☐<br>probable ☐<br>unassessable ☐	mild ☐<br>moderate ☐<br>severe ☐<br>unassessable ☐	resolved - no sequelae ☐<br>resolved - sequelae ☐<br>unresolved ☐<br>death ☐<br>unknown ☐
	yes ☐<br>no ☐	unrelated ☐<br>possible ☐<br>probable ☐<br>unassessable ☐	mild ☐<br>moderate ☐<br>severe ☐<br>unassessable ☐	resolved - no sequelae ☐<br>resolved - sequelae ☐<br>unresolved ☐<br>death ☐<br>unknown ☐

Investigator _______________ Signature _____________

6 Mechanical ventilation protocol

Patient's height [cm]	IBW [kg]<br>M: 50+0.91*(height-152.4), F: 45.5+0.91*(height-152.4)

Modus	Volume controlled ventilation
FiO_2	≥40%, adjust to maintain SpO_2 ≥93%
I:E ratio	1:2
RR	adjust to normocapnia ($ETCO_2$ 35-45mmHg or 4,6-6kPa)
PEEP	according to randomization: 4 vs. 12 cmH_2O
Inspiratory V_T	7 ml/kg IBW = ________ml

Recruitment maneuver *(perform directly* ***after*** *induction or hourly recording or disconnection)*

1. Peak inspiratory pressure limit = 55 cmH_2O
2. V_T = 7 ml/kg IBW and RR ≥ 6/min, while PEEP = 12 cmH_2O (or higher during rescue)
3. I:E = 1:1
4. Increase V_T in steps of 4 ml/kg IBW until Pplat reaches 40 – 50 cmH_2O
5. If Pplat <40 cmH_2O with highest possible V_T, increase PEEP to maximum 20 cmH_2O
6. Allow three breaths while maintaining Pplat = 40 – 50 cmH_2O
7. Set RR, I:E, inspiratory pause and V_T back to pre-recruitment values, while maintaining PEEP at 12 cmH_2O (or higher if during rescue)

7 Rescue strategy (if SpO_2 ≤ 92%)
First exclude airway problems, auto-PEEP, hemodynamic impairment and ventilator malfunction!

Conventional group

Step	FiO_2	PEEP
1	0.5	4 cmH_2O
2	0.6	4 cmH_2O
3	0.7	4 cmH_2O
4	0.8	4 cmH_2O
5	0.9	4 cmH_2O
6	1.0	4 cmH_2O
7	1.0	5 cmH_2O
8	1.0	6 cmH_2O
9	1.0	7 cmH_2O (+RM)
(+RM), recruitment maneuver optional		

Protective Group

Step*	FiO_2	PEEP
1 Exclude any hemodynamic impairment		
2	0.4	14 cmH_2O (+RM)
3	0.4	16 cmH_2O (+RM)
4	0.4	18 cmH_2O (+RM)
5	0.5	18 cmH_2O
6	0.6	18 cmH_2O
7	0.7	18 cmH_2O
8	0.8	18 cmH_2O
9	0.9	18 cmH_2O
10	1.0	18 cmH_2O
11	1.0	20 cmH_2O (+RM)
(+RM), recruitment maneuver optional<br><br>**At any step: If SpO_2 deteriorates further in an otherwise hemodynamic stable patient, consider reducing the PEEP to 10 and then 8 cmH_2O*		

Investigator _______________ Signature _____________

8 Intraoperative variables

- Record variables *within 5 min* after anesthesia induction and hourly thereafter (Induction, Hr 1, Hr 2…)
- Record recruitment variables *during* peak phase of recruitment maneuver (RM 1, RM 2…)

	Induc-tion	RM 1	Hr1	RM 2	Hr 2	RM 3	Hr 3	RM 4	Hr 4	RM 5	Hr 5
Time [hh:mm]											
Ppeak [cmH_2O]											
Pplat [cmH_2O]											
PEEP [cmH_2O]											
V_T insp [ml]											
RR [/min]											
I:E [x:x]											
FiO_2 [%]											
SpO_2 [%]											
$ETCO_2$ [mmHg / kPa]											
MAP [mmHg]											
HR [bpm]											

AE/SAE

New hypotension (BPsys < 90mmHg or BPsys drop > 10mmHg, if BPsys < 90 before)											
	yes / no	yes / no	yes / no	yes / no	yes / no	yes / no	yes / no	yes / no	yes / no	yes / no	yes / no
New bradycardia (HR <50bpm or HR drop > 20%, if HR < 50 before)											
	yes / no	yes / no	yes / no	yes / no	yes / no	yes / no	yes / no	yes / no	yes / no	yes / no	yes / no
New hypoxemia (SpO_2 ≤ 92% or SpO_2 drop > 5%, if SpO_2 < 92% before)											
	yes / no	yes / no	yes / no	yes / no	yes / no	yes / no	yes / no	yes / no	yes / no	yes / no	yes / no
Other event (please specify on page 10)											
	yes / no	yes / no	yes / no	yes / no	yes / no	yes / no	yes / no	yes / no	yes / no	yes / no	yes / no
Disconnection from the ventilator											
	—	—	yes / no	—	yes / no	—	yes / no	—	yes / no	—	yes / no
Rescue according to page 11 (if SpO_2 ≤ 92%)											
	yes / no	yes / no	yes / no	yes / no	yes / no	yes / no	yes / no	yes / no	yes / no	yes / no	yes / no

Investigator _______________ Signature _____________

9 Intraoperative variables continuation

	RM 6	Hr 6	RM 7	Hr 7	RM 8	Hr 8	RM 9	Hr 9	RM 10	Hr 10	RM 11
Time [hh:mm]											
Ppeak [cmH_2O]											
Pplat [cmH_2O]											
PEEP [cmH_2O]											
V_T insp [ml]											
RR [/min]											
I:E [x:x]											
FiO_2 [%]											
SpO_2 [%]											
$ETCO_2$ [mmHg / kPa]											
MAP [mmHg]											
HR [bpm]											

AE/SAE

New hypotension (BPsys < 90mmHg or BPsys drop > 10mmHg, if BPsys < 90 before)											
	yes / no	yes / no	yes / no	yes / no	yes / no	yes / no	yes / no	yes / no	yes / no	yes / no	yes / no
New bradycardia (HR <50bpm or HR drop > 20%, if HR < 50 before)											
	yes / no	yes / no	yes / no	yes / no	yes / no	yes / no	yes / no	yes / no	yes / no	yes / no	yes / no
New hypoxemia ($SpO_2 \leq 92\%$ or SpO_2 drop > 5%, if $SpO_2 < 92\%$ before)											
	yes / no	yes / no	yes / no	yes / no	yes / no	yes / no	yes / no	yes / no	yes / no	yes / no	yes / no
Other event (please specify on page 10)											
	yes / no	yes / no	yes / no	yes / no	yes / no	yes / no	yes / no	yes / no	yes / no	yes / no	yes / no
Disconnection from the ventilator											
	———	yes / no	———	yes / no	———	yes / no	———	yes / no	———	yes / no	———
Rescue according to page 11 (if $SpO_2 \leq 92\%$)											
	yes / no	yes / no	yes / no	yes / no	yes / no	yes / no	yes / no	yes / no	yes / no	yes / no	yes / no

Investigator _______________ Signature _____________

10 Intraoperative variables continuation

	Hr 11	RM 12	Hr 12	RM 13	Hr 13	RM 14	Hr 14	RM 15	Hr 15	RM 16	Hr 16
Time [hh:mm]											
Ppeak [cmH_2O]											
Pplat [cmH_2O]											
PEEP [cmH_2O]											
V_T insp [ml]											
RR [/min]											
I:E [x:x]											
FiO_2 [%]											
SpO_2 [%]											
$ETCO_2$ [mmHg / kPa]											
MAP [mmHg]											
HR [bpm]											

AE/SAE

New hypotension (BPsys < 90mmHg or BPsys drop > 10mmHg, if BPsys < 90 before)											
	yes / no	yes / no	yes / no	yes / no	yes / no	yes / no	yes / no	yes / no	yes / no	yes / no	yes / no
New bradycardia (HR <50bpm or HR drop > 20%, if HR < 50 before)											
	yes / no	yes / no	yes / no	yes / no	yes / no	yes / no	yes / no	yes / no	yes / no	yes / no	yes / no
New hypoxemia (SpO_2 ≤ 92% or SpO_2 drop > 5%, if SpO_2 < 92% before)											
	yes / no	yes / no	yes / no	yes / no	yes / no	yes / no	yes / no	yes / no	yes / no	yes / no	yes / no
Other event (please specify on page 10)											
	yes / no	yes / no	yes / no	yes / no	yes / no	yes / no	yes / no	yes / no	yes / no	yes / no	yes / no
Disconnection from the ventilator											
	yes / no	—	yes / no	—	yes / no	—	yes / no	—	yes / no	—	yes / no
Rescue according to page 11 (if SpO_2 ≤ 92%)											
	yes / no	yes / no	yes / no	yes / no	yes / no	yes / no	yes / no	yes / no	yes / no	yes / no	yes / no

Investigator ________________ Signature ______________

POSTOPERATIVE DAY 1 (first 24hrs period)

report events within this period if not stated otherwise

1 Recovery

Lost to follow up	yes ☐	no ☐	if yes	reason	
Continuation of MV directly after surgery	yes ☐	no ☐	if yes	duration [hrs]	indication:
New requirement of NIV	yes ☐	no ☐	if yes	CPAP ☐ NPPV ☐	duration [hrs]
				maximum intensity [pressure level]:	
			indication	standard of care ☐ resp. failure ☐	
New requirement of invasive MV	yes ☐	no ☐	if yes	duration [hrs]	
			indication	resurgery ☐ resp. failure ☐ other ☐	
ICU stay	yes ☐	no ☐	if yes	preop scheduled ☐ unscheduled ☐	
PONV	yes ☐	no ☐			
Physiotherapy	yes ☐	no ☐			
Breathing exercises	yes ☐	no ☐	if yes	incentive spirometry	yes ☐ no ☐
Cumulated Ambulation Score (page 34) [0-6]:					
Impairment of wound healing	yes ☐	no ☐	if yes	superficial ☐ deep ☐	
Surgical wound infection	yes ☐	no ☐	if yes	superficial ☐ deep ☐	
			if yes	abscess ☐ empyema ☐ phlegmon ☐	
Return of bowel function	yes ☐	no ☐			

2 Fluids/ Drugs

			cumulative dose			cumulative dose
Artificial Colloids [ml]	HES	yes ☐	______	Crystalloids [ml]	yes ☐	______
	Gelatine	yes ☐	______	Albumin [ml]	yes ☐	______
	Dextran	yes ☐				
Transfusion [ml]	PRBC	yes ☐	______	FFP	yes ☐	______
	FP24	yes ☐	______	Fibrinogen [g]	yes ☐	______
	Cryoprecipitate	yes ☐	______	PPSB [IU]	yes ☐	______
	Platelets	yes ☐	______	other	yes ☐	______
			if other	type:		

Antibiotics	yes ☐	no ☐	if yes	prophylaxis ☐ therapy ☐
Vasoactive drugs	yes ☐	no ☐	if yes	Dobutamine ☐ Dopamine ☐ Epinephrine ☐
				Norepinephrine ☐ Phenylephrine ☐ other ☐
			if other	type

Investigator ______________ Signature ______________

3.1 Actual organ function – mandatory measurements (status at visit, 12-24hrs after end of surgery)

SpO_2 beach chair position + 10 min in room air possible?	yes ☐ no ☐	if yes	SpO_2 [%]:
		if no	SpO_2 [%]: **and** FiO_2 [%] (page 35):
RR [/min]			
HR [/min]		ABP mean [mmHg]	
Temperature [°C]		tympanic ☐ axillar ☐ inguinal ☐ oral ☐ rectal ☐ other ☐	if other specify:
Airway secretion	yes ☐ no ☐	if yes	purulent ☐ not purulent ☐
VAS dyspnea [1-10cm]		VAS thoracic pain [1-10cm]	
VAS abdominal rest pain [1-10cm]		VAS abdominal incident pain [1-10cm]	

3.2 Non-mandatory measurements

**Spirometry**			**Laboratory tests**		
FVC [L]	FVC[% predicted]		Hb	mmol/l ☐	g/dl ☐
FEV_1 [L/1sec]	FEV_1 [% predicted]		WBC	GPt/L	
			Platelets	GPt/L	
**Chest X-ray** obtained	yes ☐	no ☐	PT	INR	
if yes			PTT	sec	
infiltrates	yes ☐	no ☐	Creatinine	µmol/l ☐	mg/dl ☐
pleural effusion	yes ☐	no ☐	BUN	mmol/l ☐	mg/dl ☐
atelectasis	yes ☐	no ☐	ALT	µmol/s*l ☐	U/L ☐
pneumothorax	yes ☐	no ☐	AST	µmol/s*l ☐	U/L ☐
cardiopulmonary edema	yes ☐	no ☐	Bilirubin	µmol/l ☐	mg/dl ☐

Investigator _______________ Signature _____________

4 Pulmonary complications (see also detailed definitions, page 36)

Aspiration pneumonitis<br>resp. failure after inhalation of gastric contents	yes ☐ no ☐
Bronchospasm<br>newly expiratory wheezing treated with bronchodilators	yes ☐ no ☐
Mild respiratory failure<br>SpO_2<90% or PaO_2<60mmHg for 10min in room air, responding to oxygen ≤ 2l/min	yes ☐ no ☐
Moderate respiratory failure<br>SpO_2<90% or PaO_2<60mmHg for 10min in room air, responding to oxygen > 2l/min	yes ☐ no ☐
Severe respiratory failure<br>need for non-invasive or invasive mechanical ventilation	yes ☐ no ☐
ARDS<br>according to Berlin definition	yes ☐ no ☐ if yes<br>mild ☐ moderate ☐ severe ☐
Pulmonary infection<br>new/ progressive infiltrates + 2: antibiotics, fever, leukocytosis/ leucopenia and/or purulent secretions	yes ☐ no ☐ no CXR ☐
Atelectasis<br>lung opacification with shift of surrounding tissue/ organ towards the affected area	yes ☐ no ☐ no CXR ☐
Cardiopulmonary edema<br>clinical signs of congestion + interstitial infiltrates/ increased vascular markings on chest X-ray	yes ☐ no ☐ no CXR ☐
Pleural effusion<br>blunting of costophrenic angle (standing)/ hazy opacity in one hemithorax (supine) on chest X-ray	yes ☐ no ☐ no CXR ☐
Pneumothorax<br>free air in the pleural space on chest X-ray/ ultrasonic imaging	yes ☐ no ☐ no CXR ☐
New pulmonary infiltrates<br>monolateral/ bilateral infiltrates without other clinical signs	yes ☐ no ☐ no CXR ☐

5 Extrapulmonary complications (see also detailed definitions, page 37)

SIRS<br>≥2 findings: Temp < 36 ^{0}C or > 38 ^{0}C; HR > 90 bpm, RR > 20 bpm; WBC < 4.000 or > 12.000/µl	yes ☐ no ☐
Sepsis<br>SIRS in response to a confirmed infective process	yes ☐ no ☐
Severe Sepsis<br>Sepsis with organ dysfunction, hypoperfusion or hypotension	yes ☐ no ☐
Septic shock<br>Sepsis with refractory hypoperfusion or hypotension despite adequate fluid resuscitation	yes ☐ no ☐
Extrapulmonary infection<br>wound infection + any other (extrapulmonary) infection	yes ☐ no ☐
Coma<br>Glasgow-Coma-Scale ≤ 8 without therapeutic coma/ sedatives	yes ☐ no ☐
Acute myocardial infarction<br>rise/ fall of cardiac markers + symptoms/ ECG changes/ /imaging of cardiac ischemia/sudden death	yes ☐ no ☐
Acute renal failure<br>**R**isk: increased Crea x1.5/ GFR decrease > 25% *or* urine output (UO) < 0.5 ml/kg/h x 6 hr<br>**I**njury: increased Crea x2 or GFR decrease > 50% *or* UO < 0.5 ml/kg/h x 12 hr<br>**F**ailure: increase Crea x3 or GFR decrease > 75% *or* UO < 0.3 ml/kg/h x 24 hr or anuria x 12 hrs<br>**L**oss: complete loss of kidney function > 4 weeks	yes ☐ no ☐ if yes<br>R ☐ I ☐ F ☐ L ☐
Disseminated intravascular coagulation<br>according to DIC score > 5	yes ☐ no ☐
Hepatic failure<br>bilirubin on postop day5/day1 > 1,7 + INR on postop day5/day1 > 1,0	yes ☐ no ☐
Gastrointestinal failure<br>1 = enteral feeding with under 50% of calculated needs or no feeding 3 days after surgery<br>2 = food intolerance (FI) *or* intra–abdominal hypertension (IAH)<br>3 = FI and IAH<br>4 = abdominal compartment syndrome (ACS)	yes ☐ no ☐ if yes<br>1 ☐ 2 ☐ 3 ☐ 4 ☐

Investigator _______________ Signature _____________

POSTOPERATIVE DAY 2 (last 24hrs period)

report events within this period of not stated otherwise

1 Recovery

Lost to follow up	yes ☐	no ☐	if yes	reason
New requirement of NIV	yes ☐	no ☐	if yes	CPAP ☐ NPPV ☐ duration [hrs]<br>intensity [pressure level]:<br>standard of care ☐ treatment of resp. failure ☐
New requirement of invasive MV	yes ☐	no ☐	if yes<br>indication	duration [hrs]<br>resurgery ☐ resp. failure ☐ other ☐
ICU stay	yes ☐	no ☐	if yes	preop scheduled ☐ unscheduled ☐<br>indication:
Physiotherapy	yes ☐	no ☐		
Breathing exercises	yes ☐	no ☐	if yes	incentive spirometry yes ☐ no ☐
Cumulated Ambulation Score (page 34) [0-6]:				
Impairment of wound healing	yes ☐	no ☐	if yes	superficial ☐ deep ☐
Surgical wound infection	yes ☐	no ☐	if yes<br>if yes	superficial ☐ deep ☐<br>abscess ☐ empyema ☐ phlegmon ☐
Return of bowel function	yes ☐	no ☐		

2 Fluids/ Drugs

			cumulative dose			*cumulative dose*
Transfusion	PRBC	yes ☐	______	FFP	yes ☐	______
[ml]	FP24	yes ☐	______	Fibrinogen [g]	yes ☐	______
	Cryoprecipitate	yes ☐	______	PPSB [IU]	yes ☐	______
	Platelets	yes ☐	______	other	yes ☐	______
			if other	type:		

Antibiotics	yes ☐	no ☐	if yes	prophylaxis ☐ therapy ☐
Vasoactive drugs	yes ☐	no ☐	if yes<br><br>if yes	Dobutamine ☐ Dopamine ☐ Epinephrine ☐<br>Norepinephrine ☐ Phenylephrine ☐ other ☐<br>type

Investigator ______________ Signature _____________

3.1 Actual organ function – mandatory measurements (status at visit)

SpO_2 beach chair position + 10 min in room air possible?	yes ☐ no ☐	if yes	SpO_2 [%]:
		if no	SpO_2 [%]: **and** FiO_2 [%] (page 35):
RR [/min]			
HR [/min]		ABP mean [mmHg]	
Temperature [°C]		tympanic ☐ axillar ☐ inguinal ☐ oral ☐ rectal ☐ other ☐	if other specify:
Airway secretion	yes ☐ no ☐	if yes	purulent ☐ not purulent ☐
VAS dyspnea [1-10cm]		VAS thoracic pain [1-10cm]	
VAS abdominal rest pain [1-10cm]		VAS abdominal incident pain [1-10cm]	

3.2 Not mandatory measurements

**Spirometry**		**Laboratory tests**		
FVC [L]	FVC[% predicted]	Hb	mmol/l ☐	g/dl ☐
FEV_1 [L/1sec]	FEV_1 [% predicted]	WBC	GPt/L	
		Platelets	GPt/L	
**Chest X-ray** obtained	yes ☐ no ☐	PT	INR	
if yes		PTT	sec	
infiltrates	yes ☐ no ☐	Creatinine	µmol/l ☐	mg/dl ☐
pleural effusion	yes ☐ no ☐	BUN	mmol/l ☐	mg/dl ☐
atelectasis	yes ☐ no ☐	ALT	µmol/s*l ☐	U/L ☐
pneumothorax	yes ☐ no ☐	AST	µmol/s*l ☐	U/L ☐
cardiopulmonary edema	yes ☐ no ☐	Bilirubin	µmol/l ☐	mg/dl ☐

Investigator _______________ Signature _____________

4 Pulmonary complications (see also detailed definitions, page 36)

Aspiration pneumonitis<br>resp. failure after inhalation of gastric contents	yes ☐ no ☐
Bronchospasm<br>newly expiratory wheezing treated with bronchodilators	yes ☐ no ☐
Mild respiratory failure<br>SpO_2<90% or PaO_2<60mmHg for 10min in room air, responding to oxygen ≤ 2l/min	yes ☐ no ☐
Moderate respiratory failure<br>SpO_2<90% or PaO_2<60mmHg for 10min in room air, responding to oxygen > 2l/min	yes ☐ no ☐
Severe respiratory failure<br>need for non-invasive or invasive mechanical ventilation	yes ☐ no ☐
ARDS<br>according to Berlin definition	yes ☐ no ☐ if yes<br>mild ☐ moderate ☐ severe ☐
Pulmonary infection<br>new/ progressive infiltrates + 2: antibiotics, fever, leukocytosis/ leucopenia and/or purulent secretions	yes ☐ no ☐ no CXR ☐
Atelectasis<br>lung opacification with shift of surrounding tissue/ organ towards the affected area	yes ☐ no ☐ no CXR ☐
Cardiopulmonary edema<br>clinical signs of congestion + interstitial infiltrates/ increased vascular markings on chest X-ray	yes ☐ no ☐ no CXR ☐
Pleural effusion<br>blunting of costophrenic angle (standing)/ hazy opacity in one hemithorax (supine) on chest X-ray	yes ☐ no ☐ no CXR ☐
Pneumothorax<br>free air in the pleural space on chest X-ray/ ultrasonic imaging	yes ☐ no ☐ no CXR ☐
New pulmonary infiltrates<br>monolateral/ bilateral infiltrates without other clinical signs	yes ☐ no ☐ no CXR ☐

5 Extrapulmonary complications (see also detailed definitions, page 37)

SIRS<br>≥2 findings: Temp < 36 ^{0}C or > 38 ^{0}C; HR > 90 bpm, RR > 20 bpm; WBC < 4.000 or > 12.000/µl	yes ☐ no ☐
Sepsis<br>SIRS in response to a confirmed infective process	yes ☐ no ☐
Severe Sepsis<br>Sepsis with organ dysfunction, hypoperfusion or hypotension	yes ☐ no ☐
Septic shock<br>Sepsis with refractory hypoperfusion or hypotension despite adequate fluid resuscitation	yes ☐ no ☐
Extrapulmonary infection<br>wound infection + any other (extrapulmonary) infection	yes ☐ no ☐
Coma<br>Glasgow-Coma-Scale ≤ 8 without therapeutic coma/ sedatives	yes ☐ no ☐
Acute myocardial infarction<br>rise/ fall of cardiac markers + symptoms/ ECG changes/ /imaging of cardiac ischemia/sudden death	yes ☐ no ☐
Acute renal failure<br>**R**isk: increased Crea x1.5/ GFR decrease > 25% *or* urine output (UO) < 0.5 ml/kg/h x 6 hr<br>**I**njury: increased Crea x2 or GFR decrease > 50% *or* UO < 0.5 ml/kg/h x 12 hr<br>**F**ailure: increase Crea x3 or GFR decrease > 75% *or* UO < 0.3 ml/kg/h x 24 hr or anuria x 12 hrs<br>**L**oss: complete loss of kidney function > 4 weeks	yes ☐ no ☐ if yes<br>R ☐ I ☐ F ☐ L ☐
Disseminated intravascular coagulation<br>according to DIC score > 5	yes ☐ no ☐
Hepatic failure<br>bilirubin on postop day5/day1 > 1,7 + INR on postop day5/day1 > 1,0	yes ☐ no ☐
Gastrointestinal failure<br>1 = enteral feeding with under 50% of calculated needs or no feeding 3 days after surgery<br>2 = food intolerance (FI) *or* intra–abdominal hypertension (IAH)<br>3 = FI and IAH<br>4 = abdominal compartment syndrome (ACS)	yes ☐ no ☐ if yes<br>1 ☐ 2 ☐ 3 ☐ 4 ☐

Investigator _______________ Signature _____________

POSTOPERATIVE DAY 3 (last 24hrs period)

report events within this period of not stated otherwise

1 Recovery

Lost to follow up	yes ☐	no ☐	if yes	reason
New requirement of NIV	yes ☐	no ☐	if yes	CPAP ☐ NPPV ☐ duration [hrs]<br>intensity [pressure level]:<br>standard of care ☐ treatment of resp. failure ☐
New requirement of invasive MV	yes ☐	no ☐	if yes<br>indication	duration [hrs]<br>resurgery ☐ resp. failure ☐ other ☐
ICU stay	yes ☐	no ☐	if yes	preop scheduled ☐ unscheduled ☐<br>indication:
Physiotherapy	yes ☐	no ☐		
Breathing exercises	yes ☐	no ☐	if yes	incentive spirometry yes ☐ no ☐
Cumulated Ambulation Score (page 34) [0-6]:				
Impairment of wound healing	yes ☐	no ☐	if yes	superficial ☐ deep ☐
Surgical wound infection	yes ☐	no ☐	if yes<br>if yes	superficial ☐ deep ☐<br>abscess ☐ empyema ☐ phlegmon ☐
Return of bowel function	yes ☐	no ☐		

2 Fluids/ Drugs

			cumulative dose			*cumulative dose*
Transfusion	PRBC	yes ☐	______	FFP	yes ☐	______
[ml]	FP24	yes ☐	______	Fibrinogen [g]	yes ☐	______
	Cryoprecipitate	yes ☐	______	PPSB [IU]	yes ☐	______
	Platelets	yes ☐	______	other	yes ☐	______
			if other	type:		

Antibiotics	yes ☐	no ☐	if yes	prophylaxis ☐ therapy ☐
Vasoactive drugs	yes ☐	no ☐	if yes	Dobutamine ☐ Dopamine ☐ Epinephrine ☐<br>Norepinephrine ☐ Phenylephrine ☐ other ☐
			if yes	type

Investigator ______________ Signature _____________

3.1 Actual organ function – mandatory measurements (status at visit)

SpO_2 beach chair position + 10 min in room air possible?	yes ☐ no ☐	if yes	SpO_2 [%]:	
		if no	SpO_2 [%]:	**and** FiO_2 [%] (page 35):
RR [/min]				
HR [/min]		ABP mean [mmHg]		
Temperature [°C]		tympanic ☐ axillar ☐ inguinal ☐ oral ☐ rectal ☐		
		other ☐	if other specify:	
Airway secretion	yes ☐ no ☐	if yes	purulent ☐ not purulent ☐	
VAS dyspnea [1-10cm]		VAS thoracic pain [1-10cm]		
VAS abdominal rest pain [1-10cm]		VAS abdominal incident pain [1-10cm]		

3.2 Non-mandatory measurements

**Spirometry**			**Laboratory tests**		
FVC [L]	FVC[% predicted]		Hb	mmol/l ☐	g/dl ☐
FEV_1 [L/1sec]	FEV_1 [% predicted]		WBC	GPt/L	
			Platelets	GPt/L	
**Chest X-ray** obtained	yes ☐	no ☐	PT	INR	
if yes			PTT	sec	
infiltrates	yes ☐	no ☐	Creatinine	µmol/l ☐	mg/dl ☐
pleural effusion	yes ☐	no ☐	BUN	mmol/l ☐	mg/dl ☐
atelectasis	yes ☐	no ☐	ALT	µmol/s*l ☐	U/L ☐
pneumothorax	yes ☐	no ☐	AST	µmol/s*l ☐	U/L ☐
cardiopulmonary edema	yes ☐	no ☐	Bilirubin	µmol/l ☐	mg/dl ☐

4 Pulmonary complications (see also detailed definitions, page 36)

Aspiration pneumonitis<br>resp. failure after inhalation of gastric contents	yes ☐ no ☐
Bronchospasm<br>newly expiratory wheezing treated with bronchodilators	yes ☐ no ☐
Mild respiratory failure<br>SpO_2<90% or PaO_2<60mmHg for 10min in room air, responding to oxygen ≤ 2l/min	yes ☐ no ☐
Moderate respiratory failure<br>SpO_2<90% or PaO_2<60mmHg for 10min in room air, responding to oxygen > 2l/min	yes ☐ no ☐
Severe respiratory failure<br>need for non-invasive or invasive mechanical ventilation	yes ☐ no ☐
ARDS<br>according to Berlin definition	yes ☐ no ☐ if yes<br>mild ☐ moderate ☐ severe ☐
Pulmonary infection<br>new/ progressive infiltrates + 2: antibiotics, fever, leukocytosis/ leucopenia and/or purulent secretions	yes ☐ no ☐ no CXR ☐
Atelectasis<br>lung opacification with shift of surrounding tissue/ organ towards the affected area	yes ☐ no ☐ no CXR ☐
Cardiopulmonary edema<br>clinical signs of congestion + interstitial infiltrates/ increased vascular markings on chest X-ray	yes ☐ no ☐ no CXR ☐
Pleural effusion<br>blunting of costophrenic angle (standing)/ hazy opacity in one hemithorax (supine) on chest X-ray	yes ☐ no ☐ no CXR ☐
Pneumothorax<br>free air in the pleural space on chest X-ray/ ultrasonic imaging	yes ☐ no ☐ no CXR ☐
New pulmonary infiltrates<br>monolateral/ bilateral infiltrates without other clinical signs	yes ☐ no ☐ no CXR ☐

5 Extrapulmonary complications (see also detailed definitions, page 37)

SIRS<br>≥2 findings: Temp < 36 ^{0}C or > 38 ^{0}C; HR > 90 bpm, RR > 20 bpm; WBC < 4.000 or > 12.000/µl	yes ☐ no ☐
Sepsis<br>SIRS in response to a confirmed infective process	yes ☐ no ☐
Severe Sepsis<br>Sepsis with organ dysfunction, hypoperfusion or hypotension	yes ☐ no ☐
Septic shock<br>Sepsis with refractory hypoperfusion or hypotension despite adequate fluid resuscitation	yes ☐ no ☐
Extrapulmonary infection<br>wound infection + any other (extrapulmonary) infection	yes ☐ no ☐
Coma<br>Glasgow-Coma-Scale ≤ 8 without therapeutic coma/ sedatives	yes ☐ no ☐
Acute myocardial infarction<br>rise/ fall of cardiac markers + symptoms/ ECG changes/ /imaging of cardiac ischemia/sudden death	yes ☐ no ☐
Acute renal failure<br>**R**isk: increased Crea x1.5/ GFR decrease > 25% *or* urine output (UO) < 0.5 ml/kg/h x 6 hr<br>**I**njury: increased Crea x2 or GFR decrease > 50% *or* UO < 0.5 ml/kg/h x 12 hr<br>**F**ailure: increase Crea x3 or GFR decrease > 75% *or* UO < 0.3 ml/kg/h x 24 hr or anuria x 12 hrs<br>**L**oss: complete loss of kidney function > 4 weeks	yes ☐ no ☐ if yes<br>R ☐ I ☐ F ☐ L ☐
Disseminated intravascular coagulation<br>according to DIC score > 5	yes ☐ no ☐
Hepatic failure<br>bilirubin on postop day5/day1 > 1,7 + INR on postop day5/day1 > 1,0	yes ☐ no ☐
Gastrointestinal failure<br>1 = enteral feeding with under 50% of calculated needs or no feeding 3 days after surgery<br>2 = food intolerance (FI) *or* intra–abdominal hypertension (IAH)<br>3 = FI and IAH<br>4 = abdominal compartment syndrome (ACS)	yes ☐ no ☐ if yes<br>1 ☐ 2 ☐ 3 ☐ 4 ☐

Investigator _______________ Signature _____________

POSTOPERATIVE DAY 4 (last 24hrs period)

report events within this period of not stated otherwise

1 Recovery

Lost to follow up	yes ☐	no ☐	if yes	reason
New requirement of NIV	yes ☐	no ☐	if yes	CPAP ☐ NPPV ☐ duration [hrs]<br>intensity [pressure level]:<br>standard of care ☐ treatment of resp. failure ☐
New requirement of invasive MV	yes ☐	no ☐	if yes<br>indication	duration [hrs]<br>resurgery ☐ resp. failure ☐ other ☐
ICU stay	yes ☐	no ☐	if yes	preop scheduled ☐ unscheduled ☐<br>indication:
Physiotherapy	yes ☐	no ☐		
Breathing exercises	yes ☐	no ☐	if yes	incentive spirometry yes ☐ no ☐
Cumulated Ambulation Score (page 34) [0-6]:				
Impairment of wound healing	yes ☐	no ☐	if yes	superficial ☐ deep ☐
Surgical wound infection	yes ☐	no ☐	if yes<br>if yes	superficial ☐ deep ☐<br>abscess ☐ empyema ☐ phlegmon ☐
Return of bowel function	yes ☐	no ☐		

2 Fluids/ Drugs

			cumulative dose			*cumulative dose*
Transfusion	PRBC	yes ☐	______	FFP	yes ☐	______
[ml]	FP24	yes ☐	______	Fibrinogen [g]	yes ☐	______
	Cryoprecipitate	yes ☐	______	PPSB [IU]	yes ☐	______
	Platelets	yes ☐	______	other	yes ☐	______
			if other	type:		

Antibiotics	yes ☐	no ☐	if yes	prophylaxis ☐ therapy ☐
Vasoactive drugs	yes ☐	no ☐	if yes<br>if yes	Dobutamine ☐ Dopamine ☐ Epinephrine ☐<br>Norepinephrine ☐ Phenylephrine ☐ other ☐<br>type

Investigator ______________ Signature ______________

3.1 Actual organ function – mandatory measurements (status at visit)

SpO_2 beach chair position + 10 min in room air possible?	yes ☐ no ☐	if yes	SpO_2 [%]:
		if no	SpO_2 [%]: **and** FiO_2 [%] (page 35):
RR [/min]			
HR [/min]		ABP mean [mmHg]	
Temperature [°C]		tympanic ☐ axillar ☐ inguinal ☐ oral ☐ rectal ☐	other ☐ if other specify:
Airway secretion	yes ☐ no ☐	if yes	purulent ☐ not purulent ☐
VAS dyspnea [1-10cm]		VAS thoracic pain [1-10cm]	
VAS abdominal rest pain [1-10cm]		VAS abdominal incident pain [1-10cm]	

3.2 Non-mandatory measurements

**Spirometry**		**Laboratory tests**	
FVC [L]	FVC[% predicted]	Hb	mmol/l ☐ g/dl ☐
FEV_1 [L/1sec]	FEV_1 [% predicted]	WBC	GPt/L
		Platelets	GPt/L
**Chest X-ray** obtained	yes ☐ no ☐	PT	INR
if yes		PTT	sec
infiltrates	yes ☐ no ☐	Creatinine	µmol/l ☐ mg/dl ☐
pleural effusion	yes ☐ no ☐	BUN	mmol/l ☐ mg/dl ☐
atelectasis	yes ☐ no ☐	ALT	µmol/s*l ☐ U/L ☐
pneumothorax	yes ☐ no ☐	AST	µmol/s*l ☐ U/L ☐
cardiopulmonary edema	yes ☐ no ☐	Bilirubin	µmol/l ☐ mg/dl ☐

Investigator _______________ Signature _____________

4 Pulmonary complications (see also detailed definitions, page 36)

Aspiration pneumonitis<br>resp. failure after inhalation of gastric contents	yes ☐ no ☐
Bronchospasm<br>newly expiratory wheezing treated with bronchodilators	yes ☐ no ☐
Mild respiratory failure<br>SpO_2<90% or PaO_2<60mmHg for 10min in room air, responding to oxygen ≤ 2l/min	yes ☐ no ☐
Moderate respiratory failure<br>SpO_2<90% or PaO_2<60mmHg for 10min in room air, responding to oxygen > 2l/min	yes ☐ no ☐
Severe respiratory failure<br>need for non-invasive or invasive mechanical ventilation	yes ☐ no ☐
ARDS<br>according to Berlin definition	yes ☐ no ☐ if yes<br>mild ☐ moderate ☐ severe ☐
Pulmonary infection<br>new/ progressive infiltrates + 2: antibiotics, fever, leukocytosis/ leucopenia and/or purulent secretions	yes ☐ no ☐ no CXR ☐
Atelectasis<br>lung opacification with shift of surrounding tissue/ organ towards the affected area	yes ☐ no ☐ no CXR ☐
Cardiopulmonary edema<br>clinical signs of congestion + interstitial infiltrates/ increased vascular markings on chest X-ray	yes ☐ no ☐ no CXR ☐
Pleural effusion<br>blunting of costophrenic angle (standing)/ hazy opacity in one hemithorax (supine) on chest X-ray	yes ☐ no ☐ no CXR ☐
Pneumothorax<br>free air in the pleural space on chest X-ray/ ultrasonic imaging	yes ☐ no ☐ no CXR ☐
New pulmonary infiltrates<br>monolateral/ bilateral infiltrates without other clinical signs	yes ☐ no ☐ no CXR ☐

5 Extrapulmonary complications (see also detailed definitions, page 37)

SIRS<br>≥2 findings: Temp < 36 ^{0}C or > 38 ^{0}C; HR > 90 bpm, RR > 20 bpm; WBC < 4.000 or > 12.000/µl	yes ☐ no ☐
Sepsis<br>SIRS in response to a confirmed infective process	yes ☐ no ☐
Severe Sepsis<br>Sepsis with organ dysfunction, hypoperfusion or hypotension	yes ☐ no ☐
Septic shock<br>Sepsis with refractory hypoperfusion or hypotension despite adequate fluid resuscitation	yes ☐ no ☐
Extrapulmonary infection<br>wound infection + any other (extrapulmonary) infection	yes ☐ no ☐
Coma<br>Glasgow-Coma-Scale ≤ 8 without therapeutic coma/ sedatives	yes ☐ no ☐
Acute myocardial infarction<br>rise/ fall of cardiac markers + symptoms/ ECG changes/ /imaging of cardiac ischemia/sudden death	yes ☐ no ☐
Acute renal failure<br>**R**isk: increased Crea x1.5/ GFR decrease > 25% *or* urine output (UO) < 0.5 ml/kg/h x 6 hr<br>**I**njury: increased Crea x2 or GFR decrease > 50% *or* UO < 0.5 ml/kg/h x 12 hr<br>**F**ailure: increase Crea x3 or GFR decrease > 75% *or* UO < 0.3 ml/kg/h x 24 hr or anuria x 12 hrs<br>**L**oss: complete loss of kidney function > 4 weeks	yes ☐ no ☐ if yes<br>R ☐ I ☐ F ☐ L ☐
Disseminated intravascular coagulation<br>according to DIC score > 5	yes ☐ no ☐
Hepatic failure<br>bilirubin on postop day5/day1 > 1,7 + INR on postop day5/day1 > 1,0	yes ☐ no ☐
Gastrointestinal failure<br>1 = enteral feeding with under 50% of calculated needs or no feeding 3 days after surgery<br>2 = food intolerance (FI) *or* intra–abdominal hypertension (IAH)<br>3 = FI and IAH<br>4 = abdominal compartment syndrome (ACS)	yes ☐ no ☐ if yes<br>1 ☐ 2 ☐ 3 ☐ 4 ☐

Investigator _______________ Signature _____________

POSTOPERATIVE DAY 5 (last 24hrs period)

report events within this period of not stated otherwise

1 Recovery

Lost to follow up	yes ☐	no ☐	if yes	reason
New requirement of NIV	yes ☐	no ☐	if yes	CPAP ☐ NPPV ☐ duration [hrs]<br>intensity [pressure level]:<br>standard of care ☐ treatment of resp. failure ☐
New requirement of invasive MV	yes ☐	no ☐	if yes<br>indication	duration [hrs]<br>resurgery ☐ resp. failure ☐ other ☐
ICU stay	yes ☐	no ☐	if yes	preop scheduled ☐ unscheduled ☐<br>indication:
Physiotherapy	yes ☐	no ☐		
Breathing exercises	yes ☐	no ☐	if yes	incentive spirometry yes ☐ no ☐
Cumulated Ambulation Score (page 34) [0-6]:				
Impairment of wound healing	yes ☐	no ☐	if yes	superficial ☐ deep ☐
Surgical wound infection	yes ☐	no ☐	if yes<br>if yes	superficial ☐ deep ☐<br>abscess ☐ empyema ☐ phlegmon ☐
Return of bowel function	yes ☐	no ☐		

2 Fluids/ Drugs

			cumulative dose				*cumulative dose*
Transfusion	PRBC	yes ☐	________		FFP	yes ☐	________
[ml]	FP24	yes ☐	________		Fibrinogen [g]	yes ☐	________
	Cryoprecipitate	yes ☐	________		PPSB [IU]	yes ☐	________
	Platelets	yes ☐	________		other	yes ☐	________
				if other	type:		

Antibiotics	yes ☐	no ☐	if yes	prophylaxis ☐ therapy ☐
Vasoactive drugs	yes ☐	no ☐	if yes<br><br>if yes	Dobutamine ☐ Dopamine ☐ Epinephrine ☐<br>Norepinephrine ☐ Phenylephrine ☐ other ☐<br>type

Investigator ______________ Signature _____________

3.1 Actual organ function – mandatory measurements (status at visit)

SpO_2 beach chair position + 10 min in room air possible?	yes ☐ no ☐	if yes	SpO_2 [%]:
		if no	SpO_2 [%]: **and** FiO_2 [%] (page 35):

RR [/min]	
HR [/min]	ABP mean [mmHg]
Temperature [°C]	tympanic ☐ axillar ☐ inguinal ☐ oral ☐ rectal ☐ other ☐ if other specify:
Airway secretion yes ☐ no ☐	if yes purulent ☐ not purulent ☐
VAS dyspnea [1-10cm]	VAS thoracic pain [1-10cm]
VAS abdominal rest pain [1-10cm]	VAS abdominal incident pain [1-10cm]

3.2 Non-mandatory measurements

**Spirometry**		**Laboratory tests**		
FVC [L]	FVC[% predicted]	Hb	mmol/l ☐	g/dl ☐
FEV_1 [L/1sec]	FEV_1 [% predicted]	WBC	GPt/L	
		Platelets	GPt/L	
**Chest X-ray** obtained	yes ☐ no ☐	PT	INR	
if yes		PTT	sec	
infiltrates	yes ☐ no ☐	Creatinine	µmol/l ☐	mg/dl ☐
pleural effusion	yes ☐ no ☐	BUN	mmol/l ☐	mg/dl ☐
atelectasis	yes ☐ no ☐	ALT	µmol/s*l ☐	U/L ☐
pneumothorax	yes ☐ no ☐	AST	µmol/s*l ☐	U/L ☐
cardiopulmonary edema	yes ☐ no ☐	Bilirubin	µmol/l ☐	mg/dl ☐

4 Pulmonary complications (see also detailed definitions, page 36)

Aspiration pneumonitis<br>resp. failure after inhalation of gastric contents	yes ☐	no ☐	
Bronchospasm<br>newly expiratory wheezing treated with bronchodilators	yes ☐	no ☐	
Mild respiratory failure<br>SpO_2<90% or PaO_2<60mmHg for 10min in room air, responding to oxygen ≤ 2l/min	yes ☐	no ☐	
Moderate respiratory failure<br>SpO_2<90% or PaO_2<60mmHg for 10min in room air, responding to oxygen > 2l/min	yes ☐	no ☐	
Severe respiratory failure<br>need for non-invasive or invasive mechanical ventilation	yes ☐	no ☐	
ARDS<br>according to Berlin definition	yes ☐<br>mild ☐	no ☐<br>moderate ☐	if yes<br>severe ☐
Pulmonary infection<br>new/ progressive infiltrates + 2: antibiotics, fever, leukocytosis/ leucopenia and/or purulent secretions	yes ☐	no ☐	no CXR ☐
Atelectasis<br>lung opacification with shift of surrounding tissue/ organ towards the affected area	yes ☐	no ☐	no CXR ☐
Cardiopulmonary edema<br>clinical signs of congestion + interstitial infiltrates/ increased vascular markings on chest X-ray	yes ☐	no ☐	no CXR ☐
Pleural effusion<br>blunting of costophrenic angle (standing)/ hazy opacity in one hemithorax (supine) on chest X-ray	yes ☐	no ☐	no CXR ☐
Pneumothorax<br>free air in the pleural space on chest X-ray/ ultrasonic imaging	yes ☐	no ☐	no CXR ☐
New pulmonary infiltrates<br>monolateral/ bilateral infiltrates without other clinical signs	yes ☐	no ☐	no CXR ☐

5 Extrapulmonary complications (see also detailed definitions, page 37)

SIRS<br>≥2 findings: Temp < 36 ^{0}C or > 38 ^{0}C; HR > 90 bpm, RR > 20 bpm; WBC < 4.000 or > 12.000/µl	yes ☐	no ☐	
Sepsis<br>SIRS in response to a confirmed infective process	yes ☐	no ☐	
Severe Sepsis<br>Sepsis with organ dysfunction, hypoperfusion or hypotension	yes ☐	no ☐	
Septic shock<br>Sepsis with refractory hypoperfusion or hypotension despite adequate fluid resuscitation	yes ☐	no ☐	
Extrapulmonary infection<br>wound infection + any other (extrapulmonary) infection	yes ☐	no ☐	
Coma<br>Glasgow-Coma-Scale ≤ 8 without therapeutic coma/ sedatives	yes ☐	no ☐	
Acute myocardial infarction<br>rise/ fall of cardiac markers + symptoms/ ECG changes/ /imaging of cardiac ischemia/sudden death	yes ☐	no ☐	
Acute renal failure<br>**R**isk: increased Crea x1.5/ GFR decrease > 25% *or* urine output (UO) < 0.5 ml/kg/h x 6 hr<br>**I**njury: increased Crea x2 or GFR decrease > 50% *or* UO < 0.5 ml/kg/h x 12 hr<br>**F**ailure: increase Crea x3 or GFR decrease > 75% *or* UO < 0.3 ml/kg/h x 24 hr or anuria x 12 hrs<br>**L**oss: complete loss of kidney function > 4 weeks	yes ☐<br>R ☐ I ☐	no ☐<br>F ☐	if yes<br>L ☐
Disseminated intravascular coagulation<br>according to DIC score > 5	yes ☐	no ☐	
Hepatic failure<br>bilirubin on postop day5/day1 > 1,7 + INR on postop day5/day1 > 1,0	yes ☐	no ☐	
Gastrointestinal failure<br>1 = enteral feeding with under 50% of calculated needs or no feeding 3 days after surgery<br>2 = food intolerance (FI) *or* intra–abdominal hypertension (IAH)<br>3 = FI and IAH<br>4 = abdominal compartment syndrome (ACS)	yes ☐<br>1 ☐ 2 ☐	no ☐<br>3 ☐	if yes<br>4 ☐

Investigator _______________ Signature _____________

DISCHARGE (period from last visit to discharge) + POSTOPERATIVE DAY 90

report events within this period of not stated otherwise

1 Recovery

Lost to follow up	yes ☐	no ☐	if yes	reason
Date of discharge	/ / 20			Postop day of discharge [1-90]
Hospital free days on day 90				
New requirement of NIV	yes ☐	no ☐	if yes	CPAP ☐ NPPV ☐ duration [hrs]<br>intensity [pressure level]:<br>standard of care ☐ treatment of resp. failure ☐
New requirement of invasive MV	yes ☐	no ☐	if yes<br>indication	duration [hrs]<br>resurgery ☐ resp. failure ☐ other ☐
ICU stay	yes ☐	no ☐	if yes	preop scheduled ☐ unscheduled ☐<br>indication:
Cumulated Ambulation Score (actual state, page 34) [0-6]:				
Impairment of wound healing	yes ☐	no ☐	if yes	superficial ☐ deep ☐
Surgical wound infection	yes ☐	no ☐	if yes<br>if yes	superficial ☐ deep ☐<br>abscess ☐ empyema ☐ phlegmon ☐
Antibiotics	yes ☐	no ☐	if yes	prophylaxis ☐ therapy ☐

Investigator _______________ Signature _____________

2.1 Actual organ function – mandatory measurements (status at visit)

SpO_2 beach chair position + 10 min in room air possible?	yes ☐ no ☐	if yes	SpO_2 [%]:	
		if no	SpO_2 [%]:	**and** FiO_2 [%] (page 35):
RR [/min]				
HR [/min]		ABP mean [mmHg]		
Temperature [°C]		tympanic ☐ axillar ☐ inguinal ☐ oral ☐ rectal ☐ other ☐	if other specify:	
Airway secretion	yes ☐ no ☐	if yes	purulent ☐ not purulent ☐	
VAS dyspnea [1-10cm]		VAS thoracic pain [1-10cm]		
VAS abdominal rest pain [1-10cm]		VAS abdominal incident pain [1-10cm]		

2.2 Non-mandatory measurements

**Spirometry**		**Laboratory tests**			
FVC [L]	FVC[% predicted]	Hb		mmol/l ☐	g/dl ☐
FEV_1 [L/1sec]	FEV_1 [% predicted]	WBC		GPt/L	
		Platelets		GPt/L	
**Chest X-ray** obtained	yes ☐ no ☐	PT		INR	
if yes		PTT		sec	
infiltrates	yes ☐ no ☐	Creatinine		µmol/l ☐	mg/dl ☐
pleural effusion	yes ☐ no ☐	BUN		mmol/l ☐	mg/dl ☐
atelectasis	yes ☐ no ☐	ALT		µmol/s*l ☐	U/L ☐
pneumothorax	yes ☐ no ☐	AST		µmol/s*l ☐	U/L ☐
cardiopulmonary edema	yes ☐ no ☐	Bilirubin		µmol/l ☐	mg/dl ☐

3 Pulmonary complications (see also detailed definitions, page 36)

Aspiration pneumonitis<br>resp. failure after inhalation of gastric contents	yes ☐ no ☐
Bronchospasm<br>newly expiratory wheezing treated with bronchodilators	yes ☐ no ☐
Mild respiratory failure<br>SpO_2<90% or PaO_2<60mmHg for 10min in room air, responding to oxygen ≤ 2l/min	yes ☐ no ☐
Moderate respiratory failure<br>SpO_2<90% or PaO_2<60mmHg for 10min in room air, responding to oxygen > 2l/min	yes ☐ no ☐
Severe respiratory failure<br>need for non-invasive or invasive mechanical ventilation	yes ☐ no ☐
ARDS<br>according to Berlin definition	yes ☐ no ☐ if yes<br>mild ☐ moderate ☐ severe ☐
Pulmonary infection<br>new/ progressive infiltrates + 2: antibiotics, fever, leukocytosis/ leucopenia and/or purulent secretions	yes ☐ no ☐ no CXR ☐
Atelectasis<br>lung opacification with shift of surrounding tissue/ organ towards the affected area	yes ☐ no ☐ no CXR ☐
Cardiopulmonary edema<br>clinical signs of congestion + interstitial infiltrates/ increased vascular markings on chest X-ray	yes ☐ no ☐ no CXR ☐
Pleural effusion<br>blunting of costophrenic angle (standing)/ hazy opacity in one hemithorax (supine) on chest X-ray	yes ☐ no ☐ no CXR ☐
Pneumothorax<br>free air in the pleural space on chest X-ray/ ultrasonic imaging	yes ☐ no ☐ no CXR ☐
New pulmonary infiltrates<br>monolateral/ bilateral infiltrates without other clinical signs	yes ☐ no ☐ no CXR ☐

4 Extrapulmonary complications (see also detailed definitions, page 37)

SIRS<br>≥2 findings: Temp < 36 ^{0}C or > 38 ^{0}C; HR > 90 bpm, RR > 20 bpm; WBC < 4.000 or > 12.000/µl	yes ☐ no ☐
Sepsis<br>SIRS in response to a confirmed infective process	yes ☐ no ☐
Severe Sepsis<br>Sepsis with organ dysfunction, hypoperfusion or hypotension	yes ☐ no ☐
Septic shock<br>Sepsis with refractory hypoperfusion or hypotension despite adequate fluid resuscitation	yes ☐ no ☐
Extrapulmonary infection<br>wound infection + any other (extrapulmonary) infection	yes ☐ no ☐
Coma<br>Glasgow-Coma-Scale ≤ 8 without therapeutic coma/ sedatives	yes ☐ no ☐
Acute myocardial infarction<br>rise/ fall of cardiac markers + symptoms/ ECG changes/ /imaging of cardiac ischemia/sudden death	yes ☐ no ☐
Acute renal failure<br>**R**isk: increased Crea x1.5/ GFR decrease > 25% *or* urine output (UO) < 0.5 ml/kg/h x 6 hr<br>**I**njury: increased Crea x2 or GFR decrease > 50% *or* UO < 0.5 ml/kg/h x 12 hr<br>**F**ailure: increase Crea x3 or GFR decrease > 75% *or* UO < 0.3 ml/kg/h x 24 hr or anuria x 12 hrs<br>**L**oss: complete loss of kidney function > 4 weeks	yes ☐ no ☐ if yes<br>R ☐ I ☐ F ☐ L ☐
Disseminated intravascular coagulation<br>according to DIC score > 5	yes ☐ no ☐
Hepatic failure<br>bilirubin on postop day5/day1 > 1,7 + INR on postop day5/day1 > 1,0	yes ☐ no ☐
Gastrointestinal failure<br>1 = enteral feeding with under 50% of calculated needs or no feeding 3 days after surgery<br>2 = food intolerance (FI) *or* intra–abdominal hypertension (IAH)<br>3 = FI and IAH<br>4 = abdominal compartment syndrome (ACS)	yes ☐ no ☐ if yes<br>1 ☐ 2 ☐ 3 ☐ 4 ☐

Investigator _______________ Signature _____________

DEFINITIONS and SCORES

1 Waist-Hip-Ratio measurement according to WHO protocol

Waist circumference should be measured at the midpoint between the lower margin of the least palpable rib and the top of the iliac crest, using a stretch - resistant tape that provides a constant 100 g tension. Hip circumference should be measured around the widest portion of the buttocks, with the tape parallel to the floor.

For both measurements, the subject should stand with feet close together, arms at the side and body weight evenly distributed, and should wear little clothing. The subject should be relaxed, and the measurements should be taken at the end of a normal expiration. Each measurement should be repeated twice; if the measurements are within 1 cm of one another, the average should be calculated. If the difference between the two measurements exceeds 1 cm, the two measurements should be repeated

(WHO. Waist Circumference and Waist–Hip Ratio: Report of a WHO Expert Consultation. Geneva, World Health Organization (WHO), 2008)

2 STOP-BANG Score

1. Snoring	Do you snore loudly (loud enough to be heard through closed doors)?	yes ☐	no ☐
2. Tired	Do you often feel tired, fatigued, or sleepy during daytime?	yes ☐	no ☐
3. Observed	Has anyone observed you stop breathing during your sleep?	yes ☐	no ☐
4. Blood pressure	Do you have or are you being treated for high blood pressure?	yes ☐	no ☐
5. BMI	BMI more than 35 kg m^{-2}?	yes ☐	no ☐
6. Age:	Age over 50 years old?	yes ☐	no ☐
7. Neck circumference	Neck circumference >40 cm?	yes ☐	no ☐
8. Gender	Male?	yes ☐	no ☐
**Total score**	**Yes to _____ questions**		

3 Cumulated Ambulation Score (CAS)

The patient is assessed on the following functions:

	Able to perform function independently	Only able to perform function with assistance from one or two people	Unable to perform function despite assistance from two people
Transfer from supine-to-sitting-to-supine	2	1	0
Transfer from sitting-to-standing-to-sitting (from armchair)	2	1	0
Walking (with appropriate walking aid)	2	1	0

Total Score [Sum of all values on a given day]: _______

Investigator _______________ Signature _____________

4 Converting oxygen therapy from O_2 to FiO_2

Method	O_2 flow (l/min)	Estimated FiO_2 (%)
Nasal cannula	1	24
	2	28
	3	32
	4	35
	5	40
	6	44
Nasopharyngeal catheter	4	40
	5	50
	6	60
Face mask	5	40
	6-7	50
	7-8	60
Face mask with reservoir	6	60
	7	70
	8	80
	9	90
	10	95

Investigator ________________ Signature ______________

6 DEFINITIONS of pulmonary post–operative complications

- Aspiration pneumonitis:
  Defined as respiratory failure after the inhalation of regurgitated gastric contents
- Bronchospasm:
  Defined as newly detected expiratory wheezing treated with bronchodilators
- Mild respiratory failure:
  PaO_2 < 60 mmHg or SpO_2 < 90% in room air during at least 10 min air *but responding* to supplemental oxygen (excluding hypoventilation)
- Moderate respiratory failure:
  PaO_2 < 60 mmHg or SpO_2 < 90% *despite* supplemental oxygen (excluding hypoventilation)
- Severe respiratory failure:
  Need for non–invasive or invasive mechanical ventilation (excluding hypoventilation)
- ARDS:
  Mild, moderate or severe according to the Berlin definition:

**Time**	Within one week of a known clinical insult, or new/worsening respiratory symptoms		
**Chest imaging***	Bilateral opacities not fully explained by effusions, lobar/lung collapse or nodules		
**Origin of edema**	Respiratory failure not fully explained by cardiac failure or fluid overload; need objective assessment to exclude hydrostatic edema if no risk factor present (e.g., echocardiography)		
	**Mild**	**Moderate**	**Severe**
**Oxygenation****	200 < PaO_2 / FiO_2 < 300	100 < PaO_2 / FiO_2 < 200	PaO_2 / FiO_2 ≤ 100
	PEEP or CPAP ≥ 5 cmH_2O***	PEEP ≥ 5 cmH_2O	PEEP ≥ 5 cmH_2O

ARDS: acute respiratory distress syndrome; PaO_2: partial pressure of arterial oxygen; FiO_2: inspired fraction of oxygen; PEEP: positive end-expiratory pressure; CPAP: continuous positive airway pressure

*: chest X-ray or CT scan

**: if altitude higher than 1,000 meters, correction factor should be made as follows: PaO_2 / FiO_2 9 (barometric pressure/760)

***: this may be delivered non-invasively in the mild ARDS group

- Pulmonary infection:
  Defined as new or progressive radiographic infiltrate plus at least two of the following: antibiotic treatment, tympanic temperature > 38ªC, leukocytosis or leucopenia (WBC count < 4,000cells/mm3 or > 12,000cells/mm3) and/or purulent secretions

Investigator _______________ Signature _____________

- Atelectasis:

  Suggested by lung opacification with shift of the mediastinum, hilum, or hemidiaphragm towards the affected area, and compensatory overinflation in the adjacent nonatelectatic lung
- Cardiopulmonary edema:

  Defined as clinical signs of congestion, including dyspnea, edema, rales and jugular venous distention, with the chest X–ray demonstrating increase in vascular markings and diffuse alveolar interstitial infiltrates
- Pleural effusion:

  Chest X–ray demonstrating blunting of the costophrenic angle, loss of the sharp silhouette of the ipsilateral hemidiaphragm in upright position, evidence of displacement of adjacent anatomical structures, or (in supine position) a hazy opacity in one hemithorax with preserved vascular shadows
- Pneumothorax:

  Defined as air in the pleural space with no vascular bed surrounding the visceral pleura
- New pulmonary infiltrates:

  Chest X–ray demonstrating new monolateral or bilateral infiltrate without other clinical signs

7 DEFINITIONS of extra–pulmonary post–operative complications

- Systemic inflammatory response syndrome (SIRS):

  Presence of two or more of the following findings: Body temperature < 36^0C or > 38^0C – Heart rate > 90 beats per minute – Respiratory rate > 20 breaths per minute or, on blood gas, a P_aCO_2 < 32 mmHg (4.3 kPa) – WBC count < 4,000 cells/mm3 or > 12,000 cells/mm3 or > 10% band forms
- Sepsis:

  SIRS in response to a confirmed infectious process; infection can be suspected or proven (by culture, stain, or polymerase chain reaction (PCR)), or a clinical syndrome pathognomonic for infection. Specific evidence for infection includes WBCs in normally sterile fluid (such as urine or cerebrospinal fluid (CSF), evidence of a perforated viscera (free air on abdominal x–ray or CT scan, signs of acute peritonitis), abnormal chest x–ray (CXR) consistent with pneumonia (with focal opacification), or petechiae, purpura, or purpura fulminans
- Severe sepsis:

  Sepsis with organ dysfunction, hypoperfusion, or hypotension
- Septic shock:

  Sepsis with refractory arterial hypotension or hypoperfusion abnormalities in spite of adequate fluid resuscitation; signs of systemic hypoperfusion may be either end-organ dysfunction or serum lactate greater than 4 mmol/dL. Other signs include oliguria and altered mental status. Patients are defined as having septic shock if they have sepsis plus hypotension after aggressive fluid resuscitation, typically upwards of 6 liters or 40 ml/kg of crystalloid
- Extra–pulmonary infection:

  Wound infection + any other infection

Investigator _______________ Signature _____________

- Coma:

  Glasgow Coma Score ≤ 8 in the absence of therapeutic coma or sedation

- Acute myocardial infarction:

  Detection of rise and/or fall of cardiac markers (preferably troponin) with at least one value above the 99th percentile of the upper reference limit, together with: symptoms of ischemia, ECG changes indicative of new ischemia, development of pathological Q-waves, or imaging evidence of new loss of viable myocardium or new regional wall motion abnormality *Or:* sudden unexpected cardiac death, involving cardiac arrest with symptoms suggestive of cardiac ischemia (but death occurring before the appearance of cardiac markers in blood)

- Acute renal failure:

  Renal failure documented as follows: Risk: increased creatinine x1.5 or GFR decrease > 25% *or* urine output (UO) < 0.5 ml/kg/h x 6 hr – Injury: increased creatinine x2 or GFR decrease > 50% *or* UO < 0.5 ml/kg/h x 12 hr – Failure: increase creatinine x3 or GFR decrease > 75% *or* UO < 0.3 ml/kg/h x 24 hr or anuria x 12 hrs – Loss: persistent ARF = complete loss of kidney function > 4 weeks

- Disseminated intravascular coagulation:

  DIC score documented as follows: Platelet count < 50 (2 points), < 100 (1 point), or ≥ 100 (0 points) – D–dimer > 4 µg/ml (2 points), > 0.39 µg/ml (1 point) or ≤ 0.39 µg/ml (0 points) – prothrombin time > 20.5 seconds (2 points), > 17.5 seconds (1 point) or ≤ 17.5 seconds (0 points); if ≥ 5 points: overt DIC

- Hepatic failure:

  Hepatic failure during short term follow up (5 postoperative days) is considered as follows: Ratio of total bilirubin on postoperative day 5 to postoperative day 1 > 1,7 and ratio of international normalized ratio (INR) on postoperative day 5 to postoperative day 1 >1,0; during long term follow up (until postoperative day 90) at new presence of hepatic encephalopathy and coagulopathy (INR > 1,5) within 8 weeks after initial signs of liver injury (e.g. jaundice) without evidence for chronic liver disease

- Gastro–intestinal failure:

  Gastro–intestinal bleeding

  Gastro–intestinal failure (GIF) score documented as follows: 0 = normal gastrointestinal function; 1 = enteral feeding with under 50% of calculated needs or no feeding 3 days after abdominal surgery; 2 = food intolerance (FI) *or* intra–abdominal hypertension (IAH); 3 = FI and IAH; and 4 = abdominal compartment syndrome (ACS)

Investigator ________________ Signature ______________

Adverse events (AE) / severe adverse events (SAE)

Any adverse events yes ☐ no ☐ if yes specify according to table:

Event (details, including treatment)	Serious	Intervention	Recovery	Outcome
	yes ☐<br>no ☐	unrelated ☐<br>possible ☐<br>probable ☐<br>unassessable ☐	mild ☐<br>moderate ☐<br>severe ☐<br>unassessable ☐	resolved - no sequelae ☐<br>resolved - sequelae ☐<br>unresolved ☐<br>death ☐<br>unknown ☐
	yes ☐<br>no ☐	unrelated ☐<br>possible ☐<br>probable ☐<br>unassessable ☐	mild ☐<br>moderate ☐<br>severe ☐<br>unassessable ☐	resolved - no sequelae ☐<br>resolved - sequelae ☐<br>unresolved ☐<br>death ☐<br>unknown ☐
	yes ☐<br>no ☐	unrelated ☐<br>possible ☐<br>probable ☐<br>unassessable ☐	mild ☐<br>moderate ☐<br>severe ☐<br>unassessable ☐	resolved - no sequelae ☐<br>resolved - sequelae ☐<br>unresolved ☐<br>death ☐<br>unknown ☐
	yes ☐<br>no ☐	unrelated ☐<br>possible ☐<br>probable ☐<br>unassessable ☐	mild ☐<br>moderate ☐<br>severe ☐<br>unassessable ☐	resolved - no sequelae ☐<br>resolved - sequelae ☐<br>unresolved ☐<br>death ☐<br>unknown ☐
	yes ☐<br>no ☐	unrelated ☐<br>possible ☐<br>probable ☐<br>unassessable ☐	mild ☐<br>moderate ☐<br>severe ☐<br>unassessable ☐	resolved - no sequelae ☐<br>resolved - sequelae ☐<br>unresolved ☐<br>death ☐<br>unknown ☐

Investigator _______________ Signature _____________
