# Supplementary material for: Protective intraoperative ventilation with higher versus lower levels of positive end-expiratory pressure in obese patients (PROBESE): study protocol for a randomized controlled trial
Source: Trials. 2017 Apr 28;18:202. doi: 10.1186/s13063-017-1929-0 (PMC5410049; doi:10.1186/s13063-017-1929-0)
Supplement: Supplementary file 4 — Standard operating procedures (SOP) for plasma Sampling. (PDF 115 kb) [file 13063_2017_1929_MOESM4_ESM.pdf]

PROBESE
Standard Operating Procedures (SOP)
Biomarkers

Collection biomarkers

- Pre-operative = time-point 0
- Directly post-operative = time-point 1
- Day 5 post-operative = time-point 5

1. Blood withdrawal

Via venous canule (venflon):

- Apply tourniquet
- Introduce the venous canule (at least, 20G, 32 mm, pink. Preferably larger)
- Loosen the tourniquet, but do not remove completely (gentle pressure)
- Withdraw blood directly out of the venflon, without the aid of a long plastic extension piece
- Withdraw 1 vacuum “Citrate tube” (blue, plastic, 2.7 ml, BD 0.109 M citrate);
- Withdraw 1 vacuum “EDTA tube” (purple, plastic, 4 ml)
- Withdraw 1 vacuum “Heparine tube” (green, plastic, 4.5 ml)
- N.B. Take *one* vacuum precursor tube in case the venflon was installed more than 5 minutes ago or when the venflon was washed with saline (0.9% NaCl).
- Mix gently without shaking.
- <u>Bring to the laboratory within 1 hour</u>
- Date of birth and the date and time of blood withdrawal, as well as any peculiarities during blood withdrawal (e.g. difficult to obtain blood etc)

Via vein puncture (in case a venous canule will not be installed):

- Apply tourniquet
- Introduce needle (21 G green)
- Loosen the tourniquet, but keep gentle pressure
- Withdraw 1 vacuum “citrate tube” (blue, plastic, 2.7 ml, BD 0.109 M citrate);
- Withdraw 1 vacuum “EDTA tube” (purple, plastic, 4 ml)
- Withdraw 1 vacuum “Heparine tube” (green, plastic, 4.5 ml)
- Mix gently without shaking
- <u>Bring to the laboratory within 1 hour</u>
- Date of birth and the date and time of blood withdrawal, as well as any peculiarities during blood withdrawal (e.g. difficult to obtain blood etc)

2. Laboratory procedures

A. Preparation of platelet poor plasma (PPP)

- Bring freshly collected, citrate-anticoagulated (0,109 M BD) whole blood as soon as possible (without shaking, etc) to the lab within 1 hour after blood withdrawal
- Centrifuge the blood for 15 minutes at 1500 - 2000 x g without brake in a swing-out rotor, at room temperature
- Register the time at which the centrifugation of the blood is started.
- When (part of) the samples are haemolytic, please note this on the sample log.
- Carefully collect the upper 2/3 of the (platelet-poor) plasma

B. Preparation of EDTA and Heparine

- Bring freshly collected, EDTA and Heparine whole blood as soon as possible (without shaking, etc) to the lab within 1 hour after blood withdrawal
- Centrifuge each tube of blood for 15 minutes at 1500 - 2000 x g without brake in a swing-out rotor, at room temperature
- Register the time at which the centrifugation of the blood is started.
- When (part of) the samples are haemolytic, please note this on the sample log.
- Carefully collect the upper 2/3 of the plasma of both tubes

C. Storage of material

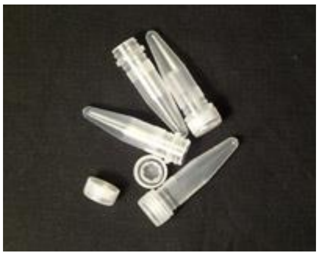

- Aliquot the plasma aliquots in conical centrifuge micro tubes of 2.0 ml
    - Citrate ≥ 0.5 ml of PPP in each tube – devided in 2 tubes
    - EDTA ≥ 1.5 ml in each tube (total 2 tubes)
    - Heparine ≥ 1.5 ml in each tube (total 2 tubes)
- Store the microtubes at -80 °C
- Mark the tubes with a sticker, which includes information on the **Institute**, **Patient Identification Number** = PIN (ask Local Investigator), and **time-point of blood collection**, to be one of the following:
  - time-point 0 (= pre-operative)
  - time-point 1 (= direct post-operative)
  - time-point 5 (= day 5 post-operative)
