# Supplementary material for: Protective intraoperative ventilation with higher versus lower levels of positive end-expiratory pressure in obese patients (PROBESE): study protocol for a randomized controlled trial
Source: Trials. 2017 Apr 28;18:202. doi: 10.1186/s13063-017-1929-0 (PMC5410049; doi:10.1186/s13063-017-1929-0)

PROBESE

Standard Operating Procedures (SOP)

Urine

Collection Urine

- Pre-operative = time-point 0
- Directly post-operative = time-point 1
- Day 5 post-operative = time-point 5

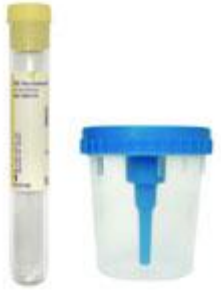

1. Urine sample collection

Spot urine:

- Let the patient collect 1 appropriate container (e.g. 100 ml) of spot urine
- Withdraw 1 vacuum “urine tube” (e.g. yellow, plastic, 10 ml)
- <u>Bring to the laboratory within 1 hour</u>
- Note date of birth and the date and time of urine withdrawal, as well as any peculiarities during collection (e.g. difficult to obtain urine etc)

Catheter urine:

- Make sure, the catheter bag was <u>emptied out < 1hr before collection</u>
- Collect 1 appropriate container (e.g. 100 ml) of catheter urine
- Withdraw 1 vacuum “urine tube” (e.g. yellow, plastic, 10 ml)
- <u>Bring to the laboratory within 1 hour</u>
- Note date of birth and the date and time of urine withdrawal, as well as any peculiarities during collection (e.g. difficult to obtain urine etc)

2. Laboratory procedures

A. Preparation

- Bring freshly collected urine <u>as soon as possible to the lab within 1 hour after collection</u>
- Centrifuge the urine for 15 minutes at 1500 - 2000 x g in a swing-out rotor, at room temperature
- Register the time at which the centrifugation of the urine is started.
- If samples are discoloured (e.g. hemolytic), please note this on the sample log.
- Carefully collect 6 ml of the upper 1/3 urine (the supernatant)

C. Storage of material

- Split the urine supernatant, 2 ml each in 3 microtubes of 2.0 ml
- Store the microtubes at -80 °C
- Mark the tubes with a sticker, which includes information on the **Institute**, **Patient Identification Number** = PIN (ask Local Investigator),
  and **time-point of urine collection**, to be one of the following:
  - time-point 0 (= pre-operative)
  - time-point 1 (= direct post-operative)
  - time-point 5 (= day 5 post-operative)

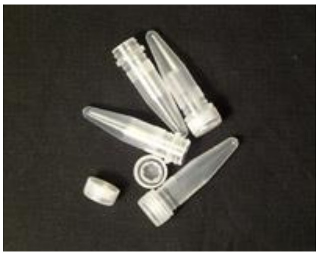

Supplement: Supplementary file 5 — Standard operating procedures (SOP) for plasma Sampling. (PDF 110 kb) [file 13063_2017_1929_MOESM5_ESM.pdf]
